# Correction: Binding blockade between TLN1 and integrin β1 represses triple-negative breast cancer

**DOI:** 10.7554/eLife.107584

**Published:** 2025-05-08

**Authors:** Yixiao Zhang, Lisha Sun, Haonan Li, Liping Ai, Qingtian Ma, Xinbo Qiao, Jie Yang, Hao Zhang, Xunyan Ou, Yining Wang, Guanglei Chen, Jinqi Xue, Xudong Zhu, Yu Zhao, Yongliang Yang, Caigang Liu

**Keywords:** Human, Mouse

Zhang Y, Sun L, Li H, Ai L, Ma Q, Qiao X, Yang J, Zhang H, Ou X, Wang Y, Chen G, Xue J, Zhu X, Zhao Y, Yang Y, Liu C. 2022. Binding blockade between TLN1 and integrin β1 represses triple-negative breast cancer. *eLife* **11**:e68481. doi: 10.7554/eLife.68481.

Published 14 March 2022

The authors regretfully acknowledge that incorrect image panels were inadvertently included in Figure 1B (“TLN1 high”) and Figure 3C (“CK18”) of the originally published article. These errors occurred due to improper data management during figure preparation, in which images from a parallel project were mistakenly saved in the same folder using similar file names. As a result, images from unrelated samples were unintentionally included in the manuscript.

For Figure 1B (“TLN1 high”), during the preparation of the immunohistochemistry panel, we inadvertently selected an image derived from a different project involving comparable histological techniques, which were not part of the TNBC study cohort. This issue was identified during a recent internal review, when inconsistencies were found between the published panel and the original pathology records. Further inspection of file naming and metadata confirmed that the image did not correspond to the clinical samples used in this study.

In Figure 3C (“CK18”), an error occurred due to the inadvertent inclusion of a Western blot image from a parallel project that was conducted under different experimental conditions. Specifically, the inserted image was from a separate experiment involving modified protein analysis parameters and did not correspond to the CK18 protein described in the figure legend. The error remained undetected initially because the image had been cropped during figure assembly, obscuring identifiable markers. Importantly, this incorrect image was not used in the quantitative analysis shown in Figure 3D and therefore does not affect the accuracy or conclusions of the data presented.

To address these issues and uphold the integrity of the publication, the authors have thoroughly reviewed all original image data and experimental records. The incorrect images have been replaced with the appropriate ones that accurately reflect the described experimental conditions.

We sincerely regret these errors and have since implemented more rigorous quality control protocols, including standardized file naming and cross-verification procedures, to prevent such mistakes in the future.

The corrected Figure 1 with updated panel B is shown here:

**Figure 1:**
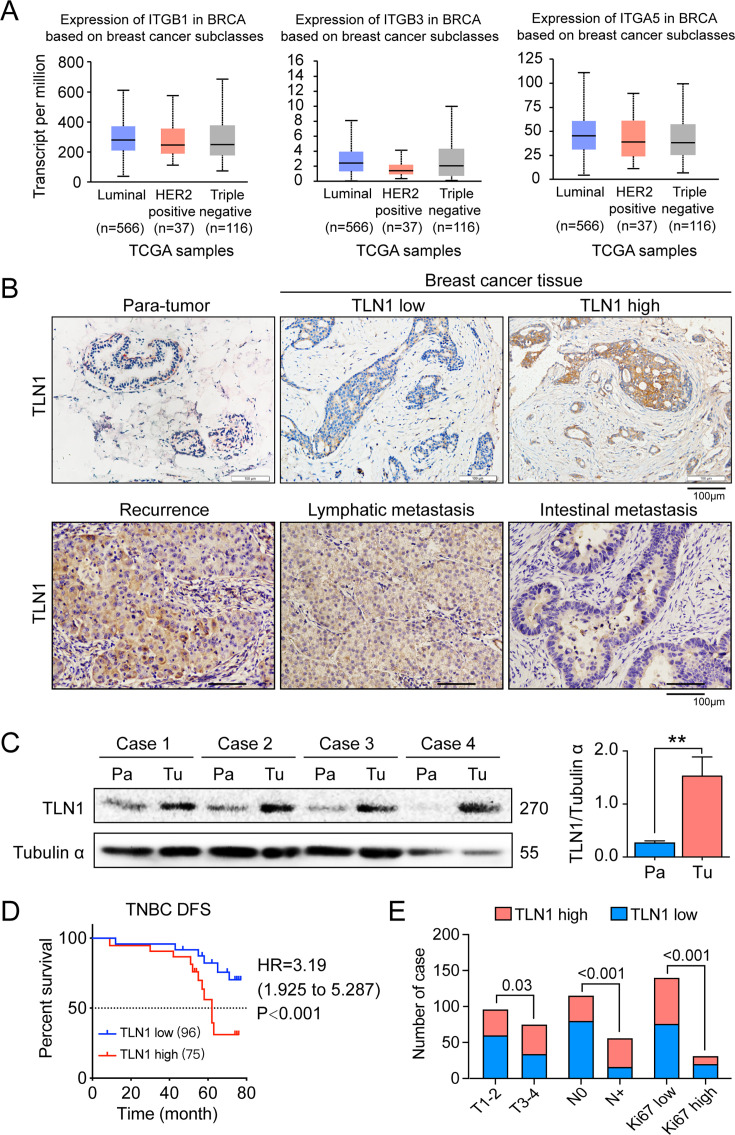


The legend associated with panel B is shown here:

Figure 1. TLN1 upregulation is associated with poor disease- free survival (DFS) in triple- negative breast cancer (TNBC).

(B) Representative immunohistochemistry images of TLN1 expression in TNBC tissue, chest wall recurrence, lymphatic metastasis, and intestinal metastasis (scale bar, 100 µm).

The originally published Figure 1 is shown for reference:

**Figure 2:**
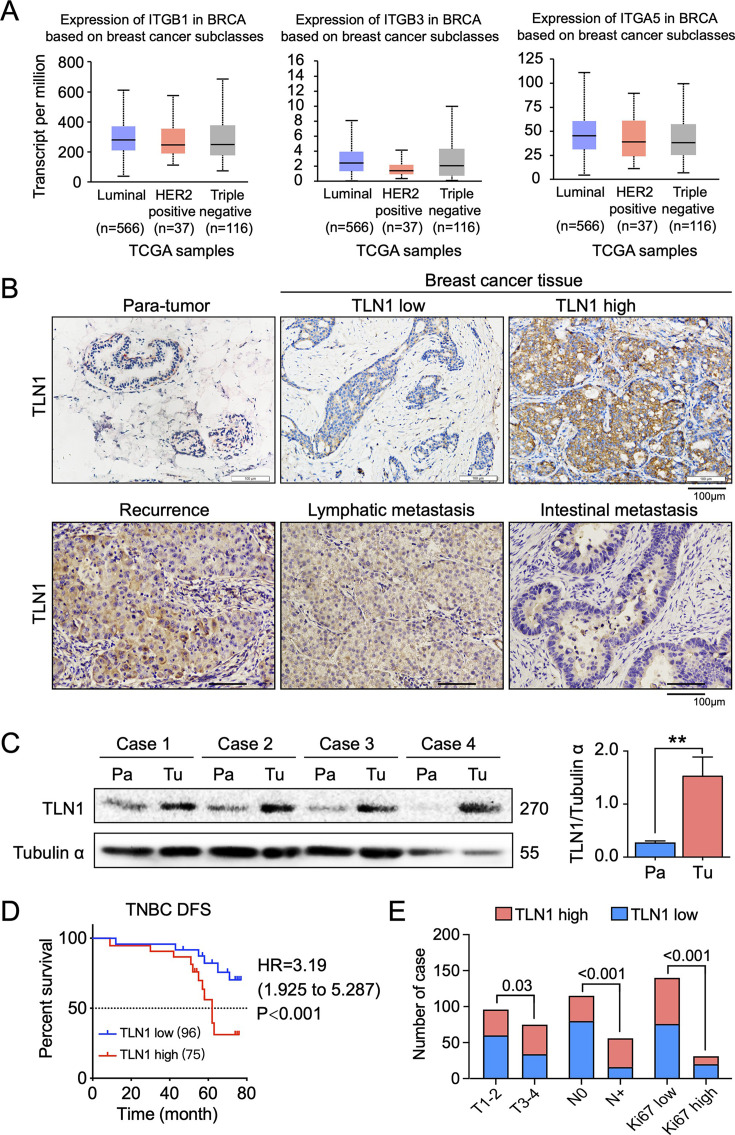


The corrected Figure 3 with updated panel C is shown here:

**Figure 3:**
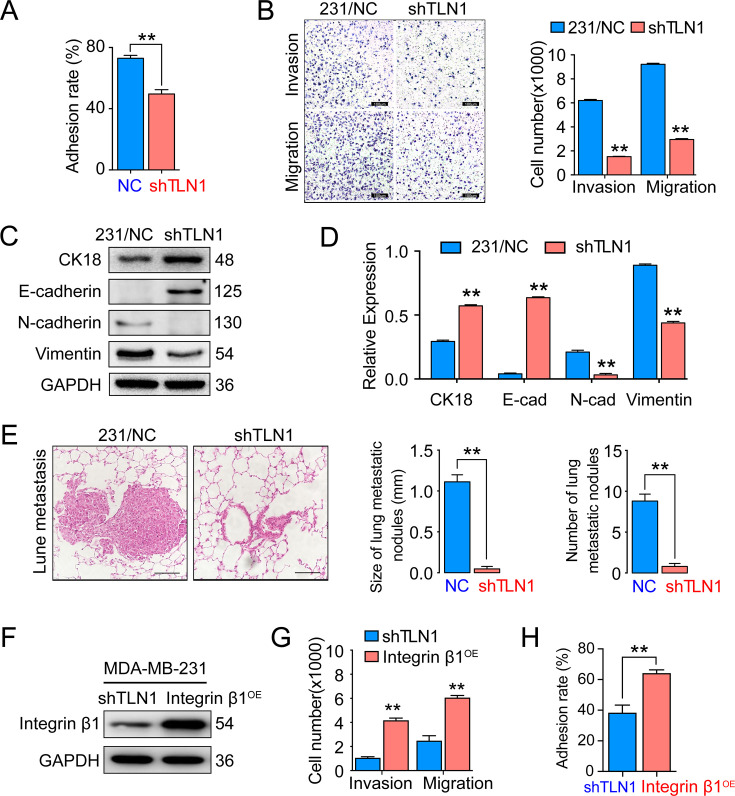


The legend associated with panel C is shown here:

Figure 3. Silencing TLN1 reduces triple- negative breast cancer (TNBC) cell motility by blocking epithelial- mesenchymal transformation (EMT).

(C) The relative expression level of CK18, E-cadherin, N-cadherin, and vimentin relative to those of GAPDH in MDA-MB- 231/NC and shTLN1 cells, using western blotting (n = 3, p < 0.01, respectively).

The originally published Figure 3 is shown for reference:

**Figure 4:**
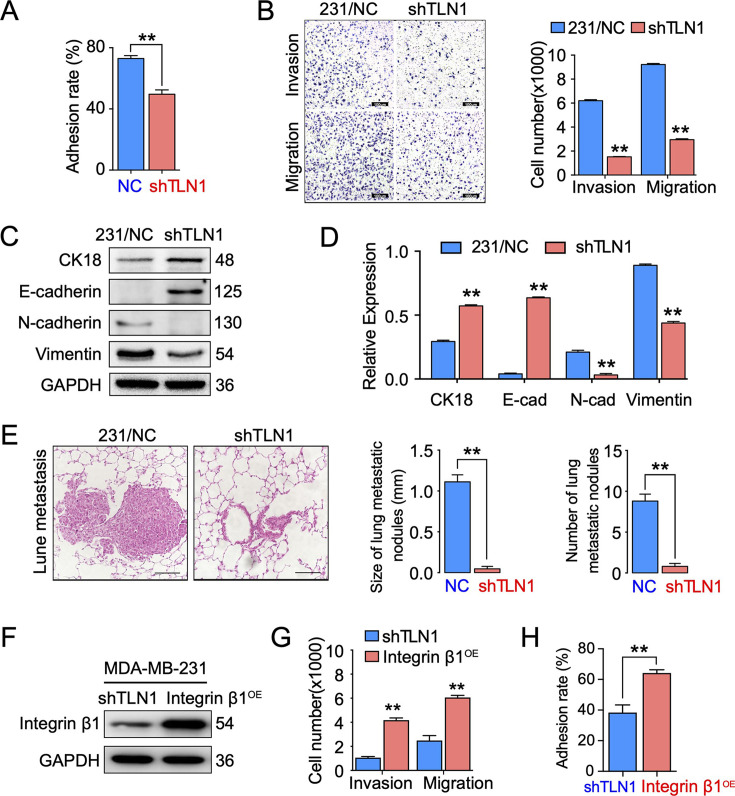


The article has been corrected accordingly.

