# Supplementary material for: Binding blockade between TLN1 and integrin β1 represses triple-negative breast cancer
Source: eLife. 2022 Mar 14;11:e68481. doi: 10.7554/eLife.68481 (PMC8937232; doi:10.7554/eLife.68481)
Supplement: Figure 1—source data 1. [file elife-68481-fig1-data1.zip › Figure 1-source data 1/1A source data.docx]

This part of the data is based on TCGA dataset analysis on the UALCAN website (http://ualcan.path.uab.edu/).
