# Supplementary figures and images for: Binding blockade between TLN1 and integrin β1 represses triple-negative breast cancer (part 1 of 2)
Source: eLife. 2022 Mar 14;11:e68481. doi: 10.7554/eLife.68481 (PMC8937232; doi:10.7554/eLife.68481)

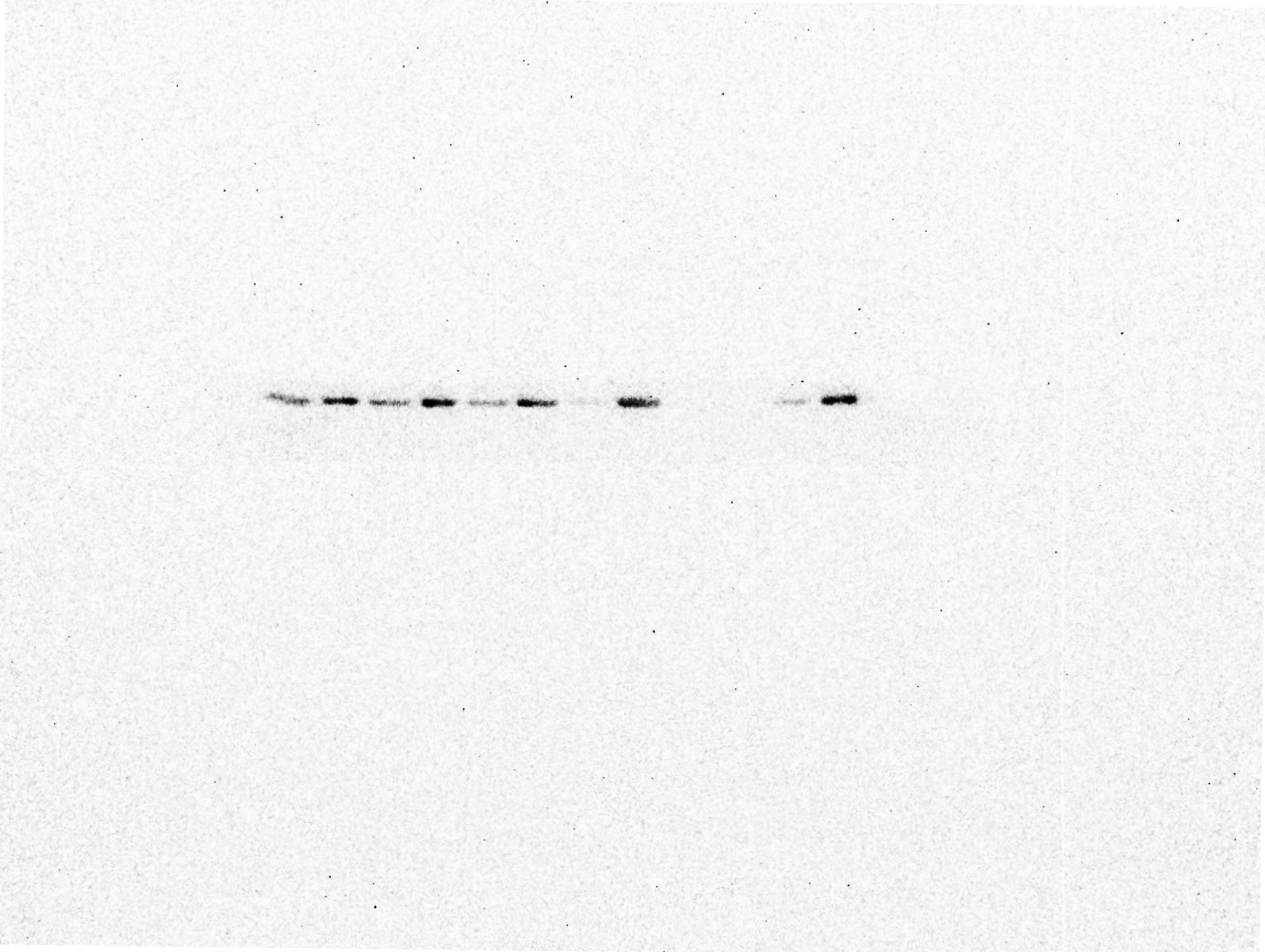

Supplement: Figure 1—source data 1. [file elife-68481-fig1-data1.zip › Figure 1-source data 1/1C source data/TLN1.jpg]

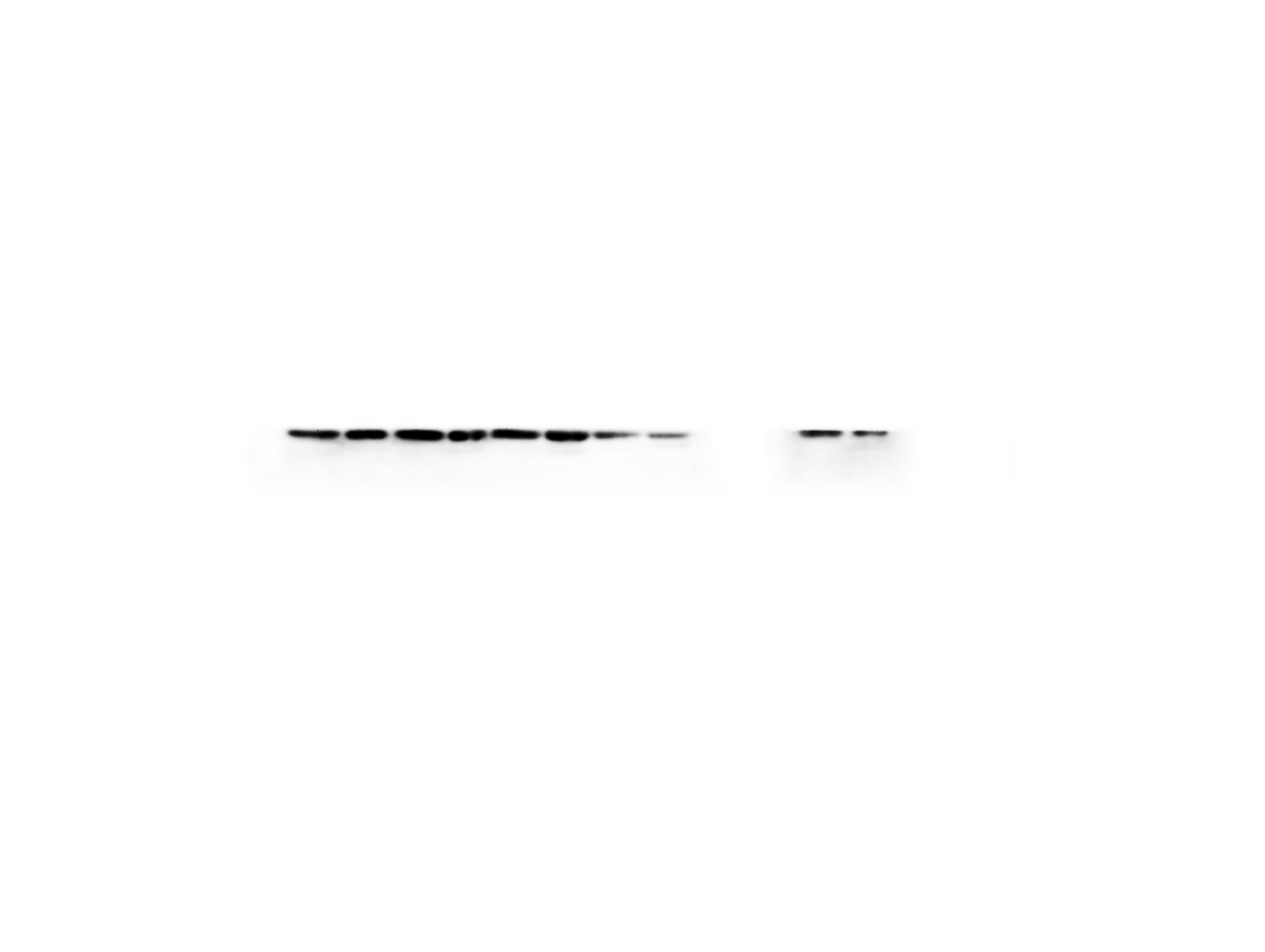

Supplement: Figure 1—source data 1. [file elife-68481-fig1-data1.zip › Figure 1-source data 1/1C source data/tubulin a.jpg]

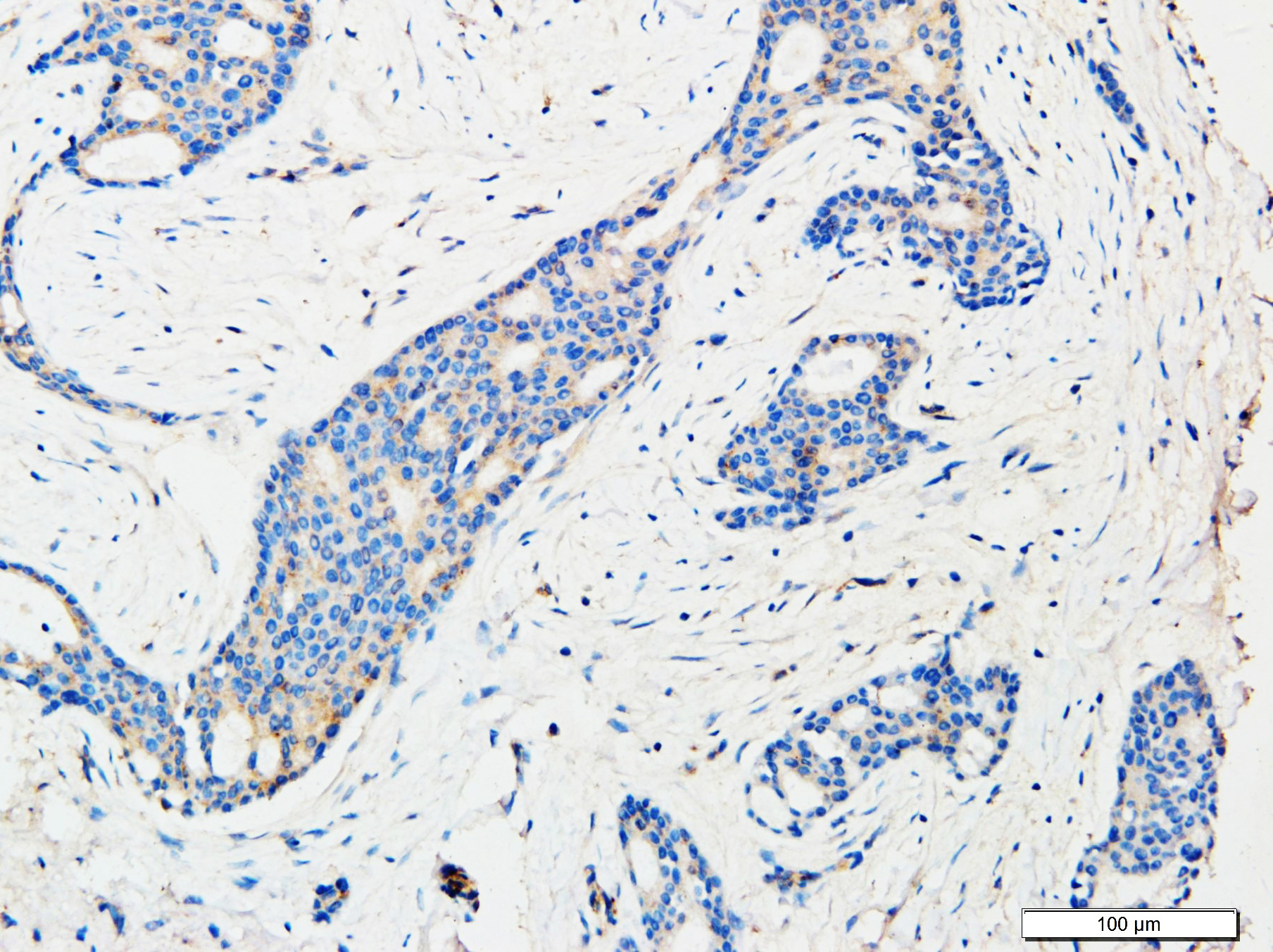

Supplement: Figure 1—source data 1. [file elife-68481-fig1-data1.zip › Figure 1-source data 1/1B source data/TLN1 low.jpg]

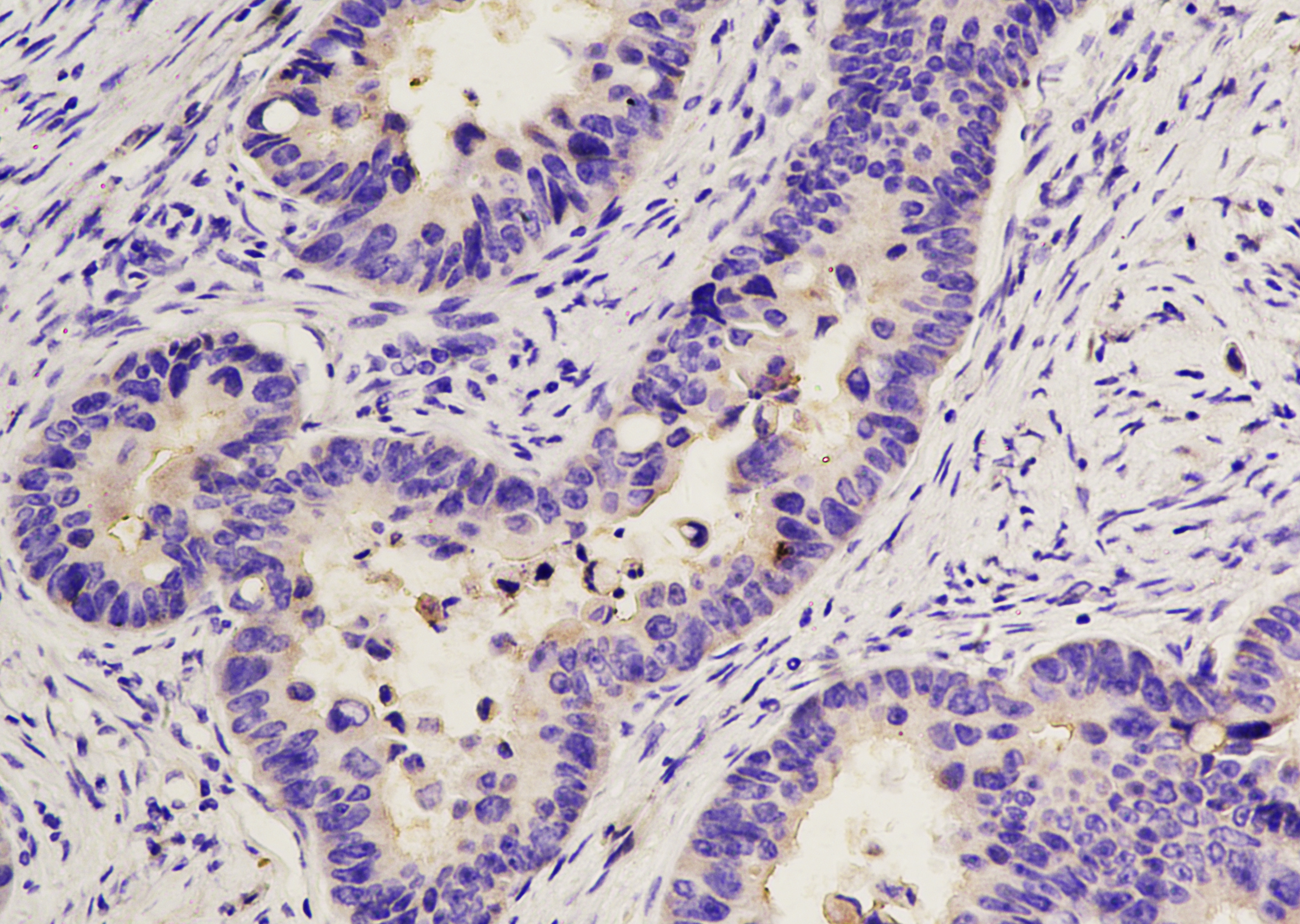

Supplement: Figure 1—source data 1. [file elife-68481-fig1-data1.zip › Figure 1-source data 1/1B source data/intestinal metastasis.tif]

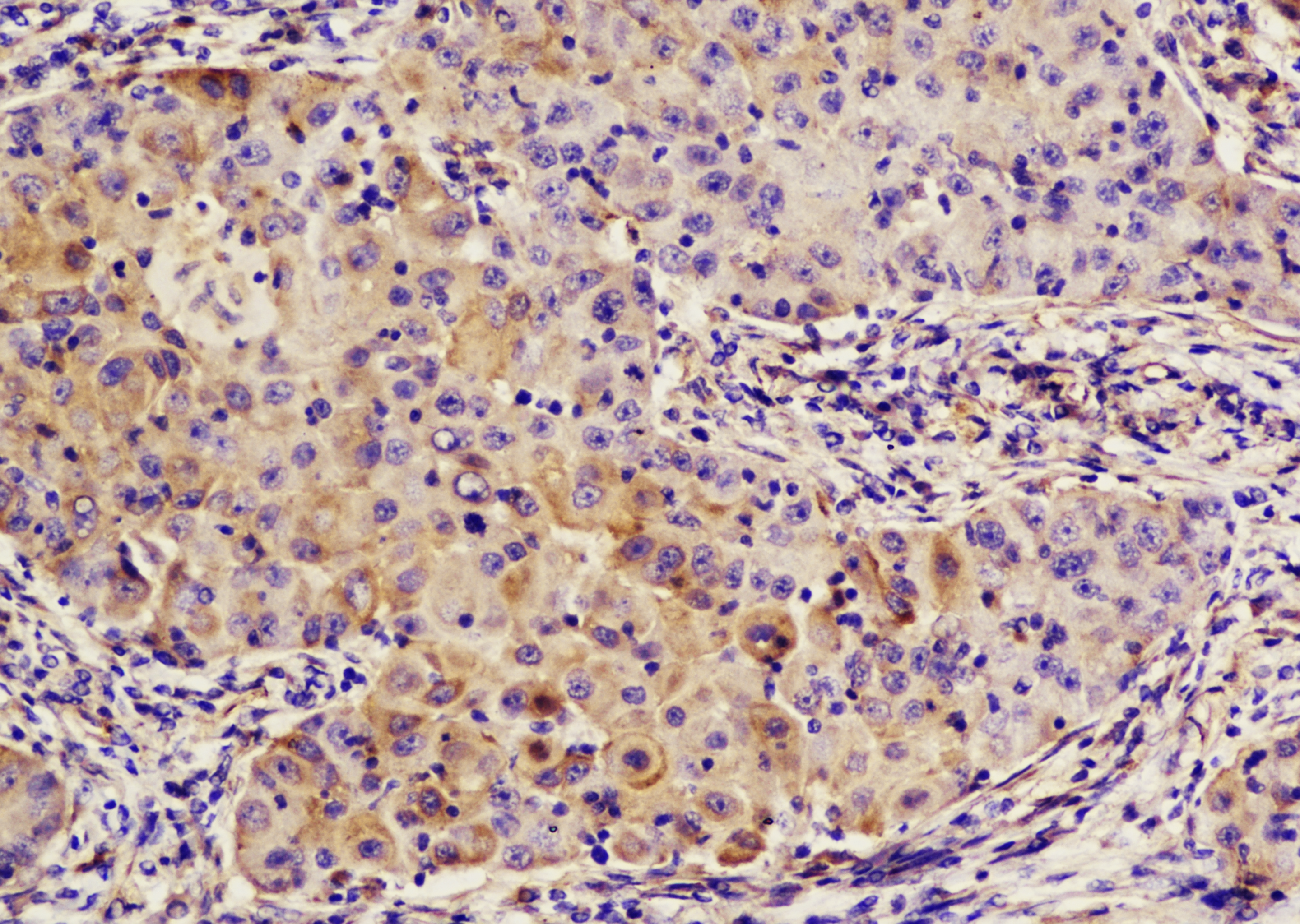

Supplement: Figure 1—source data 1. [file elife-68481-fig1-data1.zip › Figure 1-source data 1/1B source data/recurrence.tif]

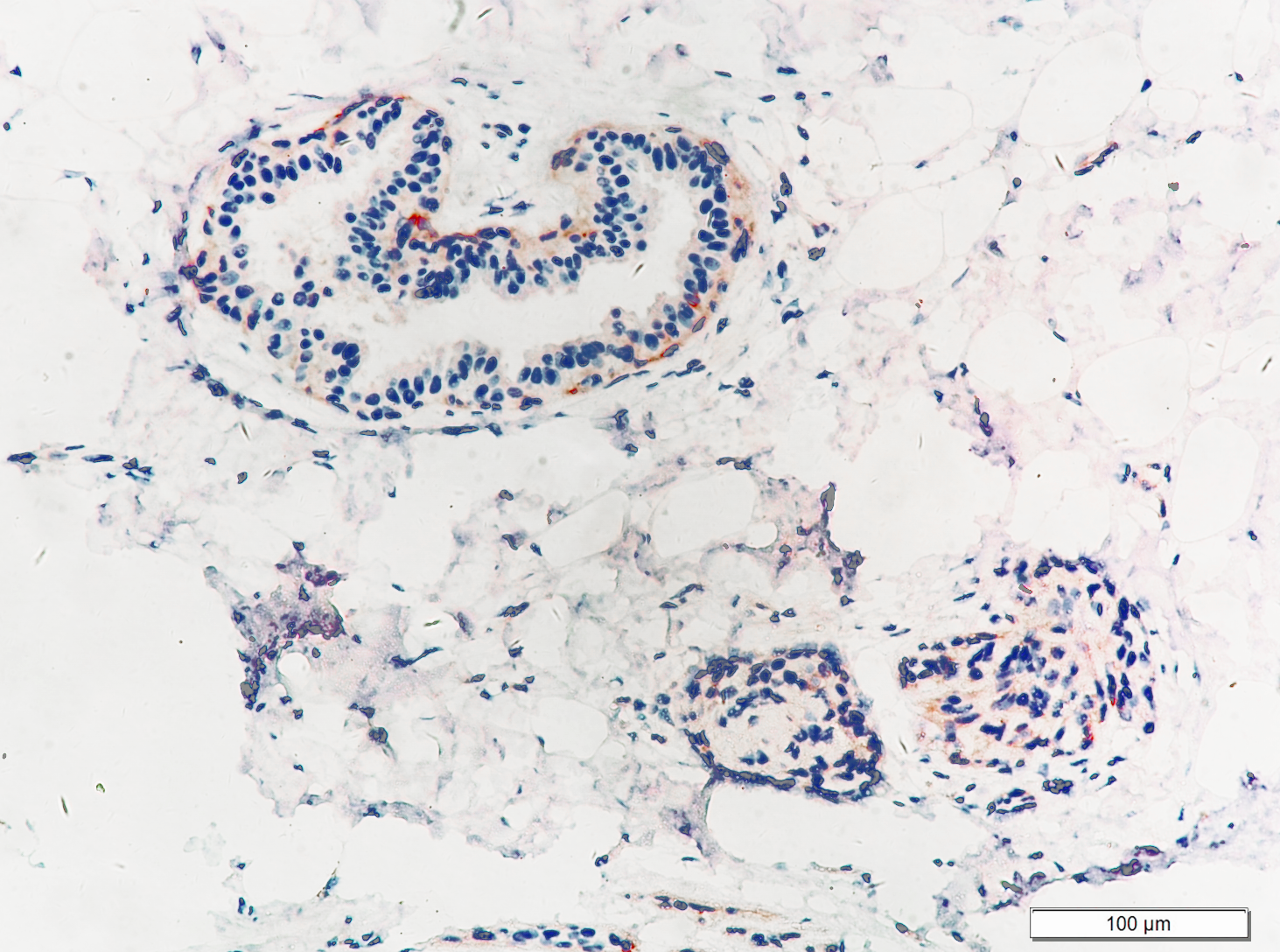

Supplement: Figure 1—source data 1. [file elife-68481-fig1-data1.zip › Figure 1-source data 1/1B source data/para-tumor.tif]

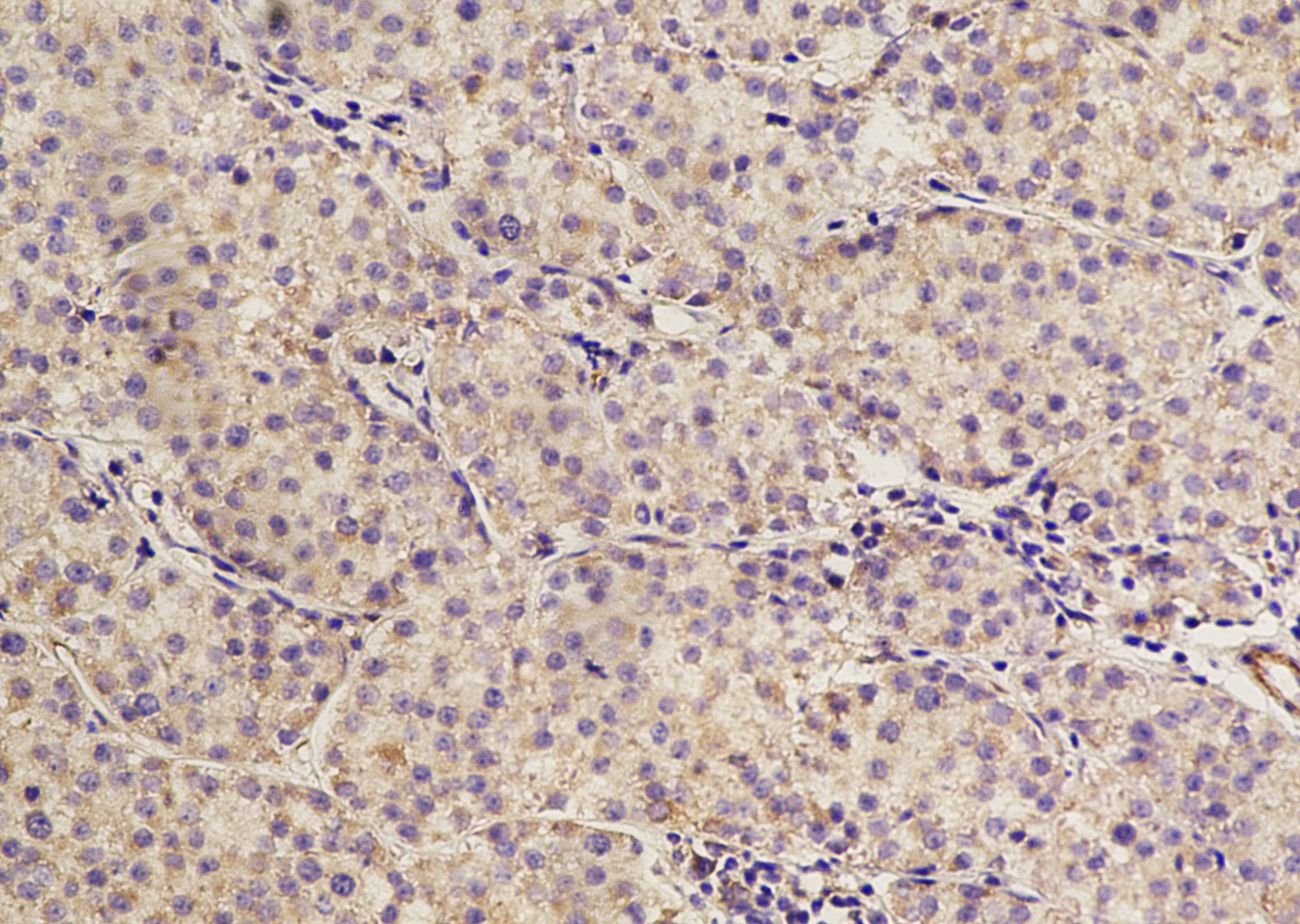

Supplement: Figure 1—source data 1. [file elife-68481-fig1-data1.zip › Figure 1-source data 1/1B source data/Lymphatic metastasis.tif]

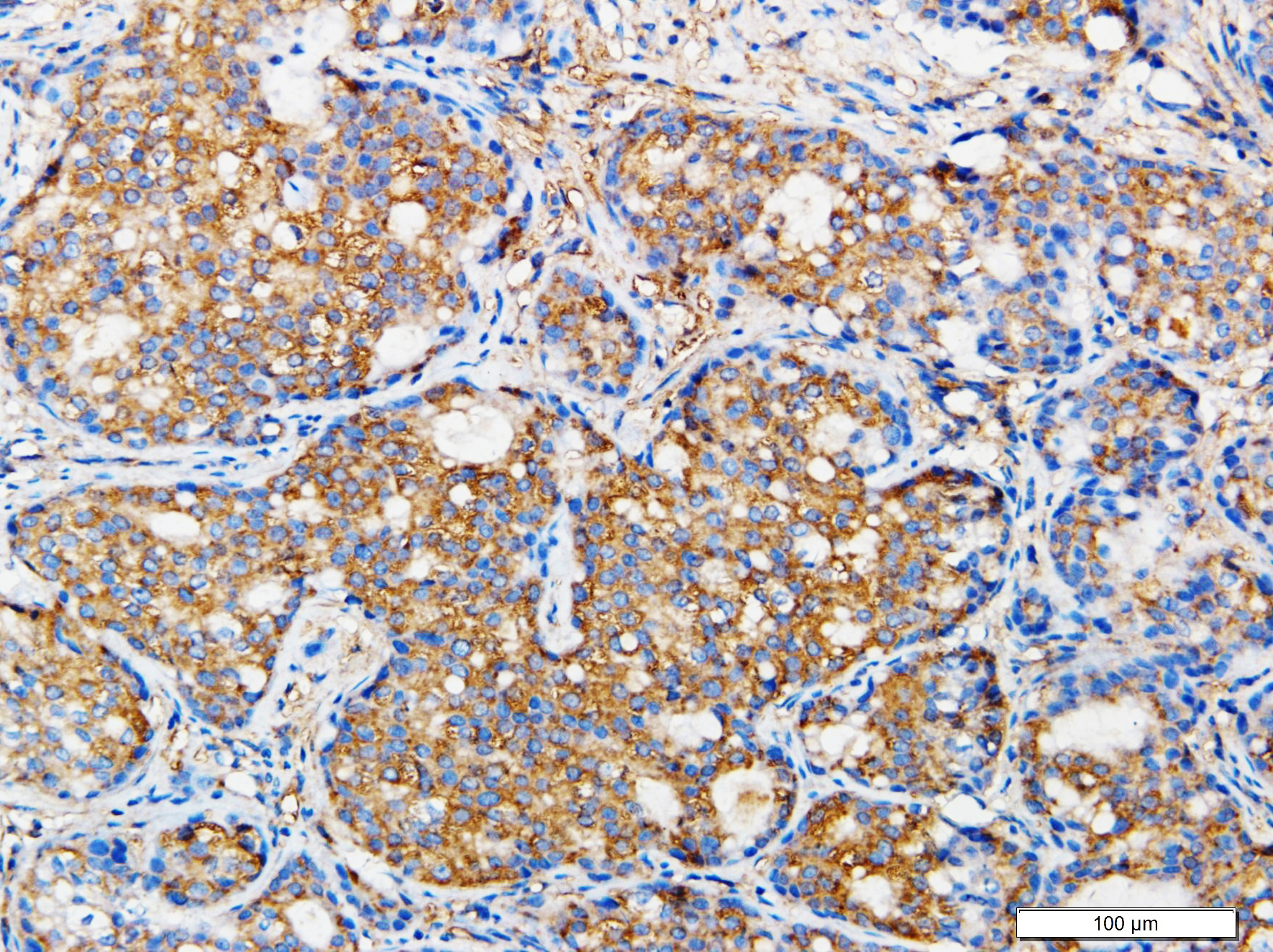

Supplement: Figure 1—source data 1. [file elife-68481-fig1-data1.zip › Figure 1-source data 1/1B source data/TLN1 high.jpg]

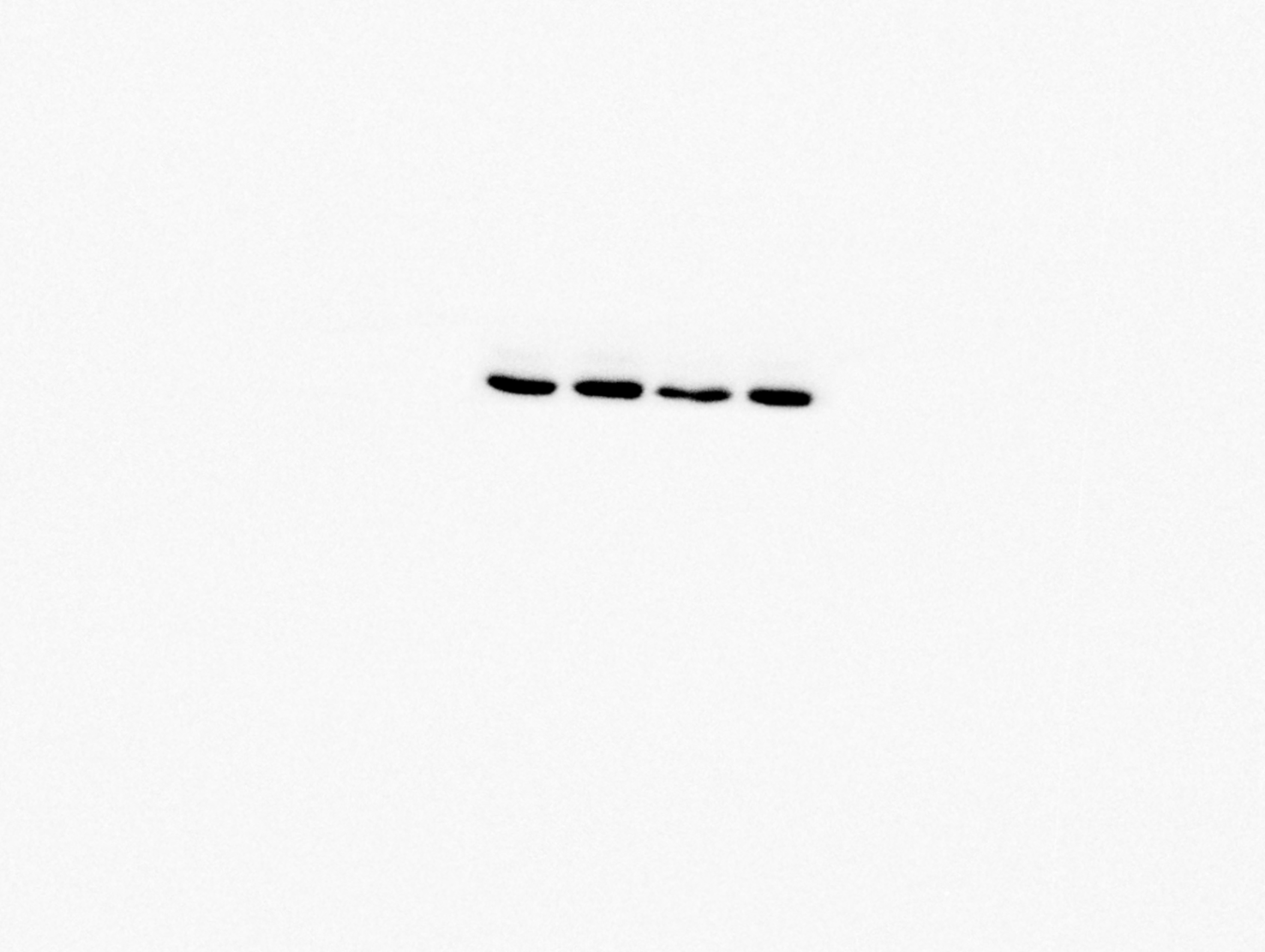

Supplement: Figure 2—source data 1. [file elife-68481-fig2-data1.zip › Figure 2-source data 1/2B source data/gapdh.tif]

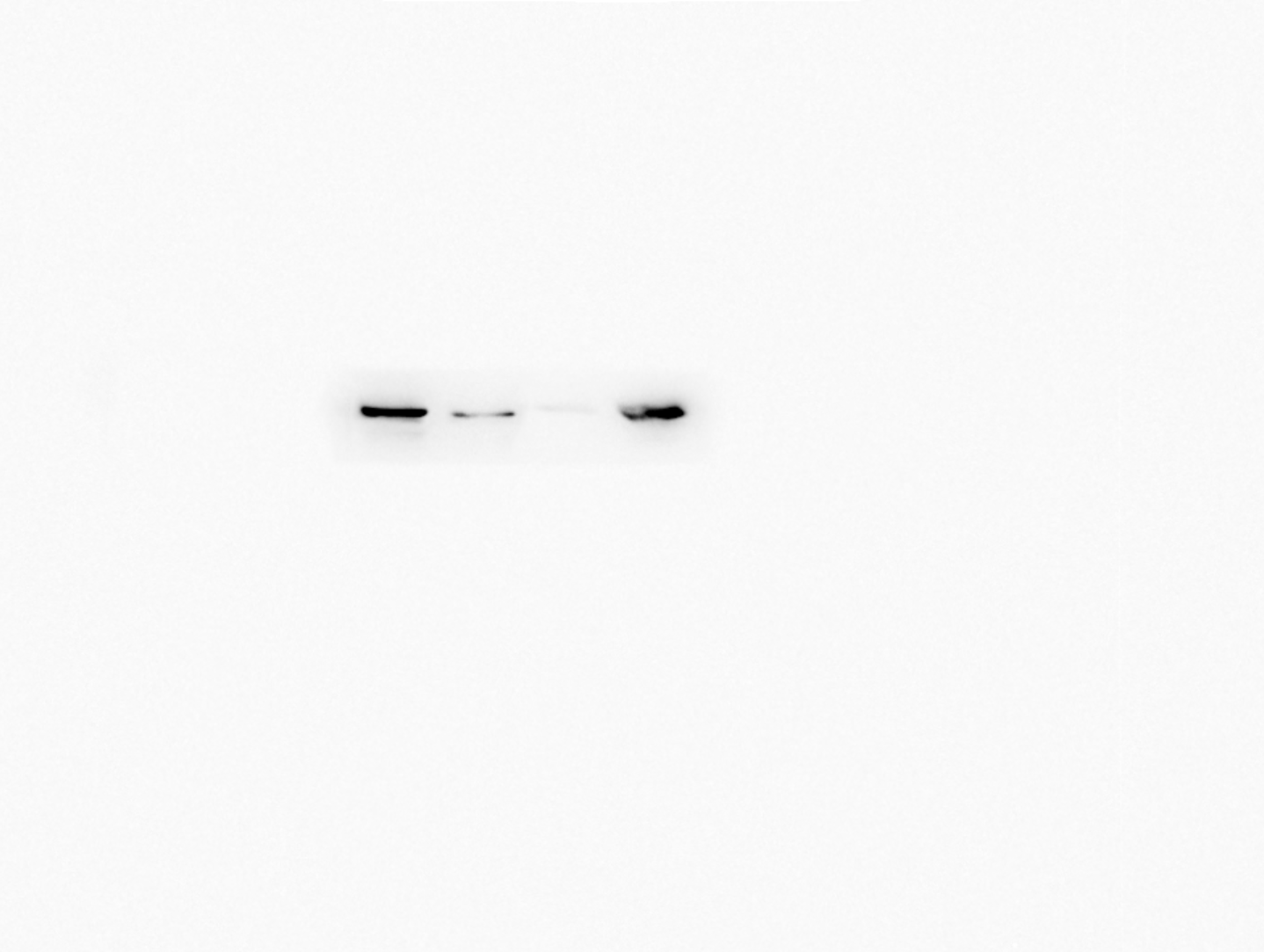

Supplement: Figure 2—source data 1. [file elife-68481-fig2-data1.zip › Figure 2-source data 1/2B source data/TLN1.tif]

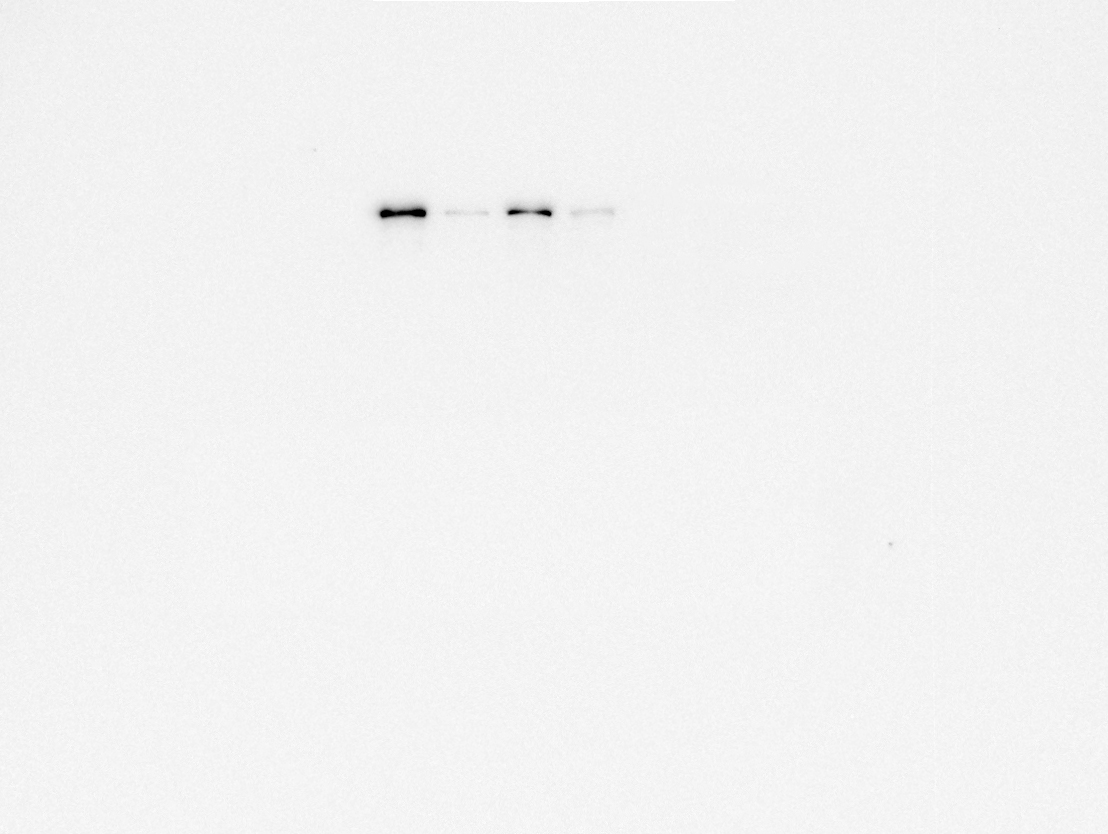

Supplement: Figure 2—source data 1. [file elife-68481-fig2-data1.zip › Figure 2-source data 1/2A source data/TLN1.jpg]

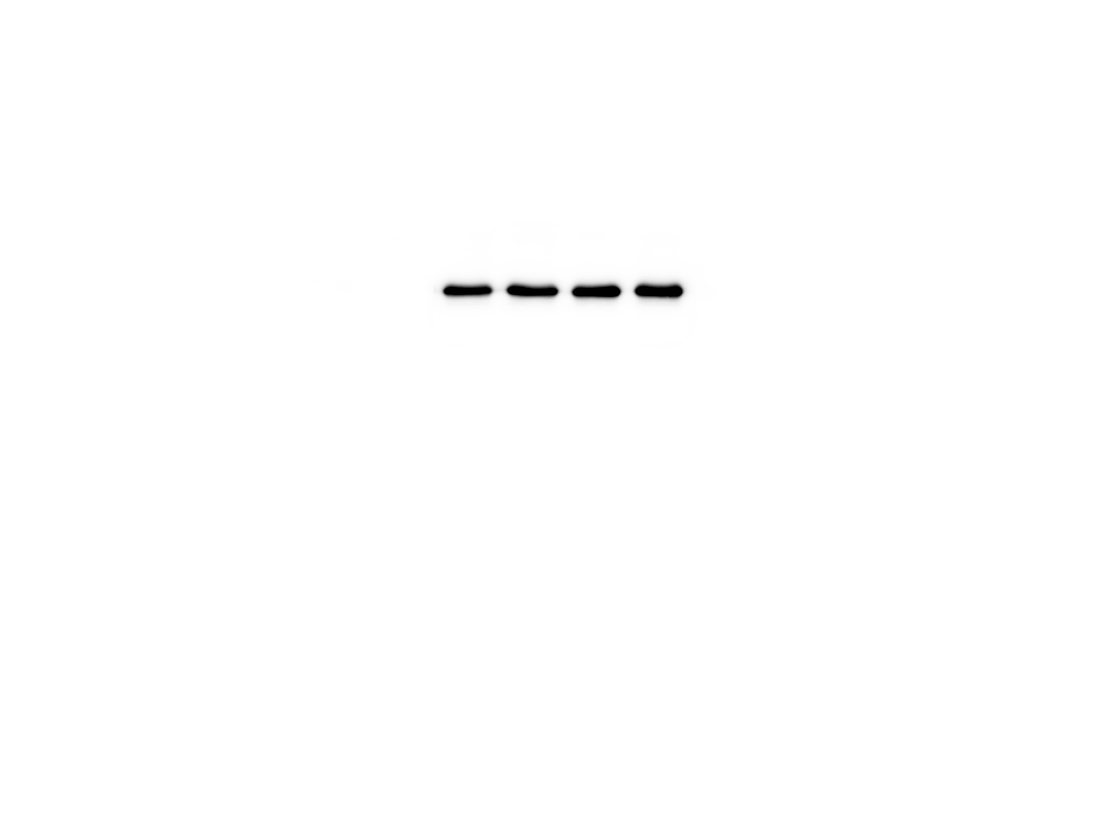

Supplement: Figure 2—source data 1. [file elife-68481-fig2-data1.zip › Figure 2-source data 1/2A source data/gapdh.jpg]

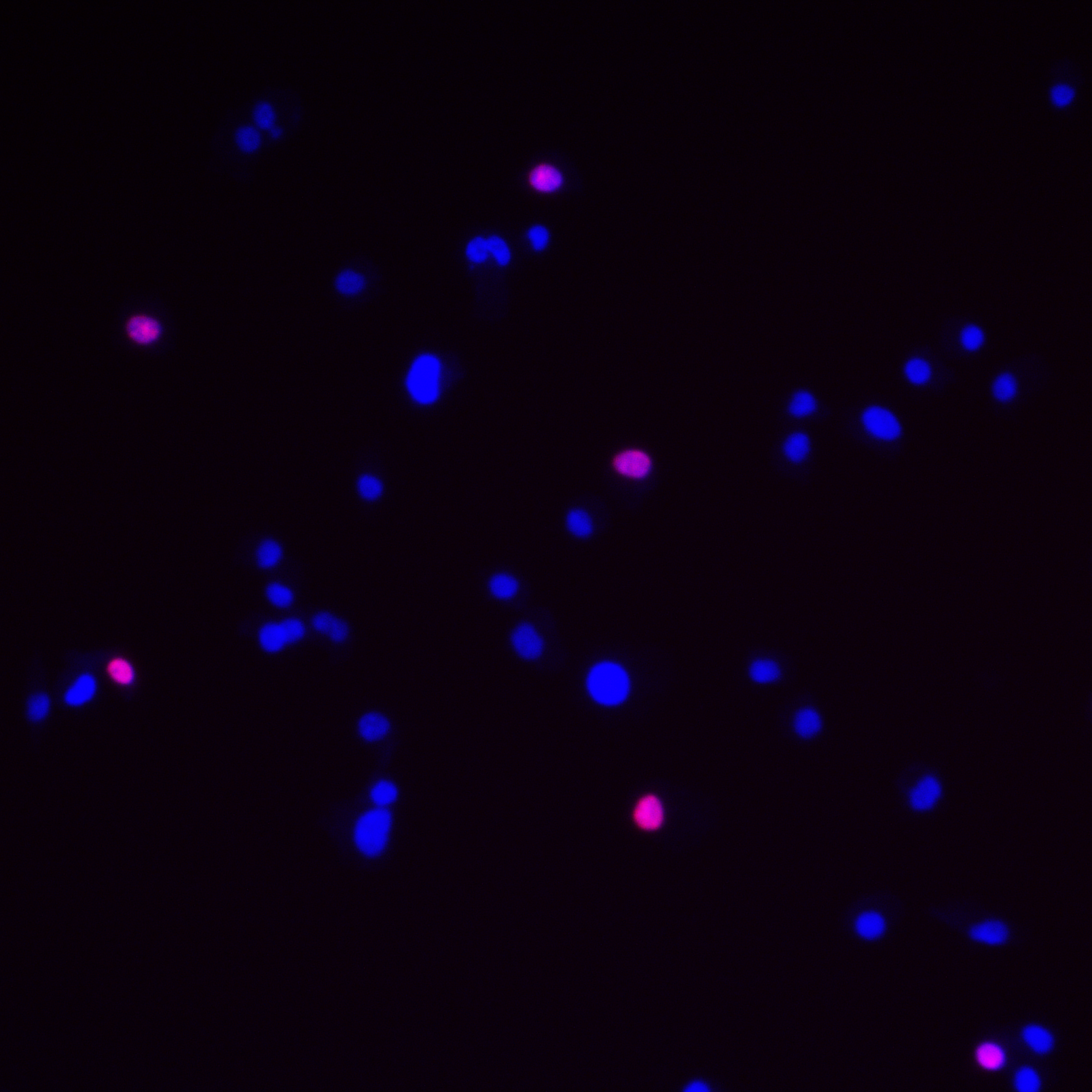

Supplement: Figure 2—source data 1. [file elife-68481-fig2-data1.zip › Figure 2-source data 1/2CDE source data/EdU/NC-0h.tif]

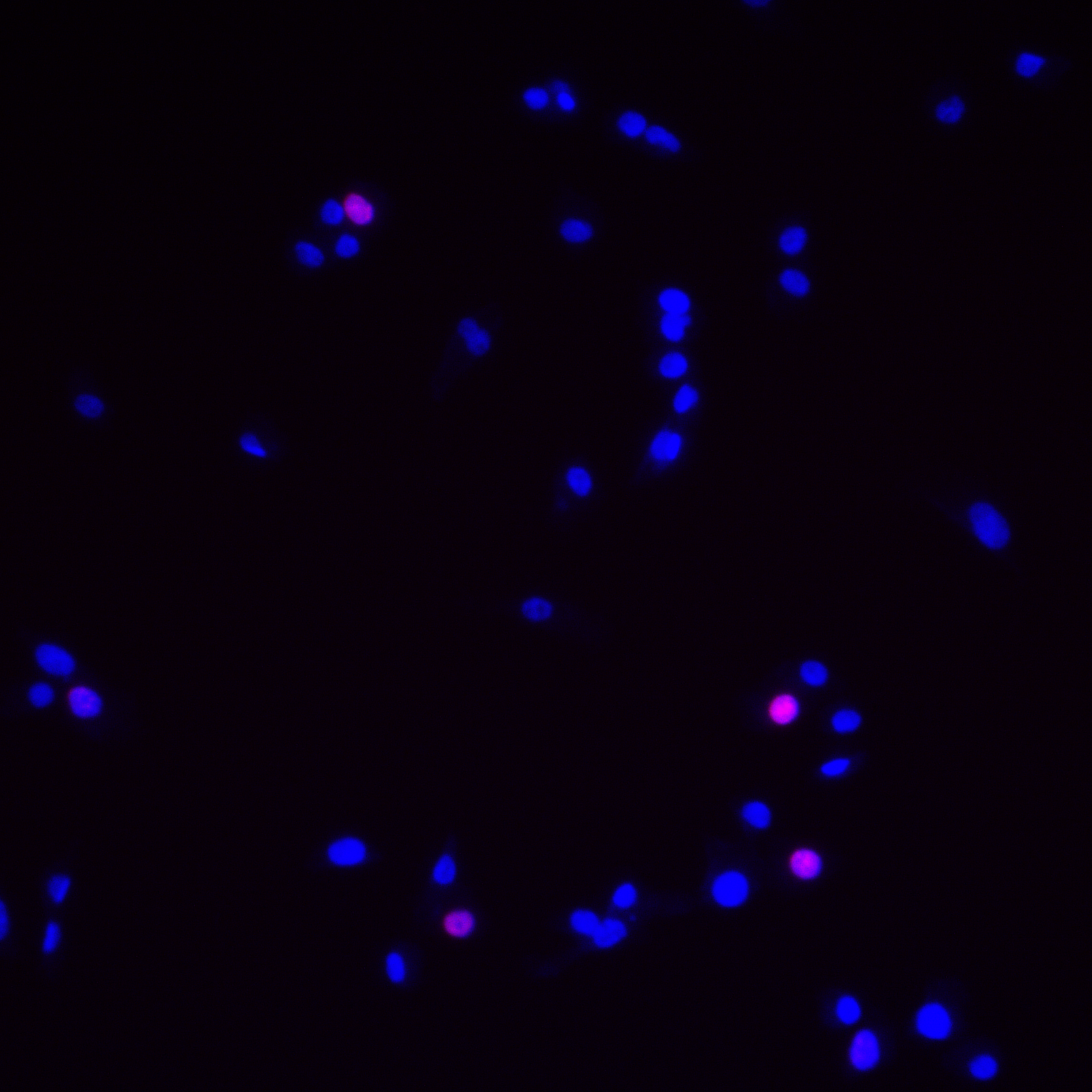

Supplement: Figure 2—source data 1. [file elife-68481-fig2-data1.zip › Figure 2-source data 1/2CDE source data/EdU/shTLN1-0h.tif]

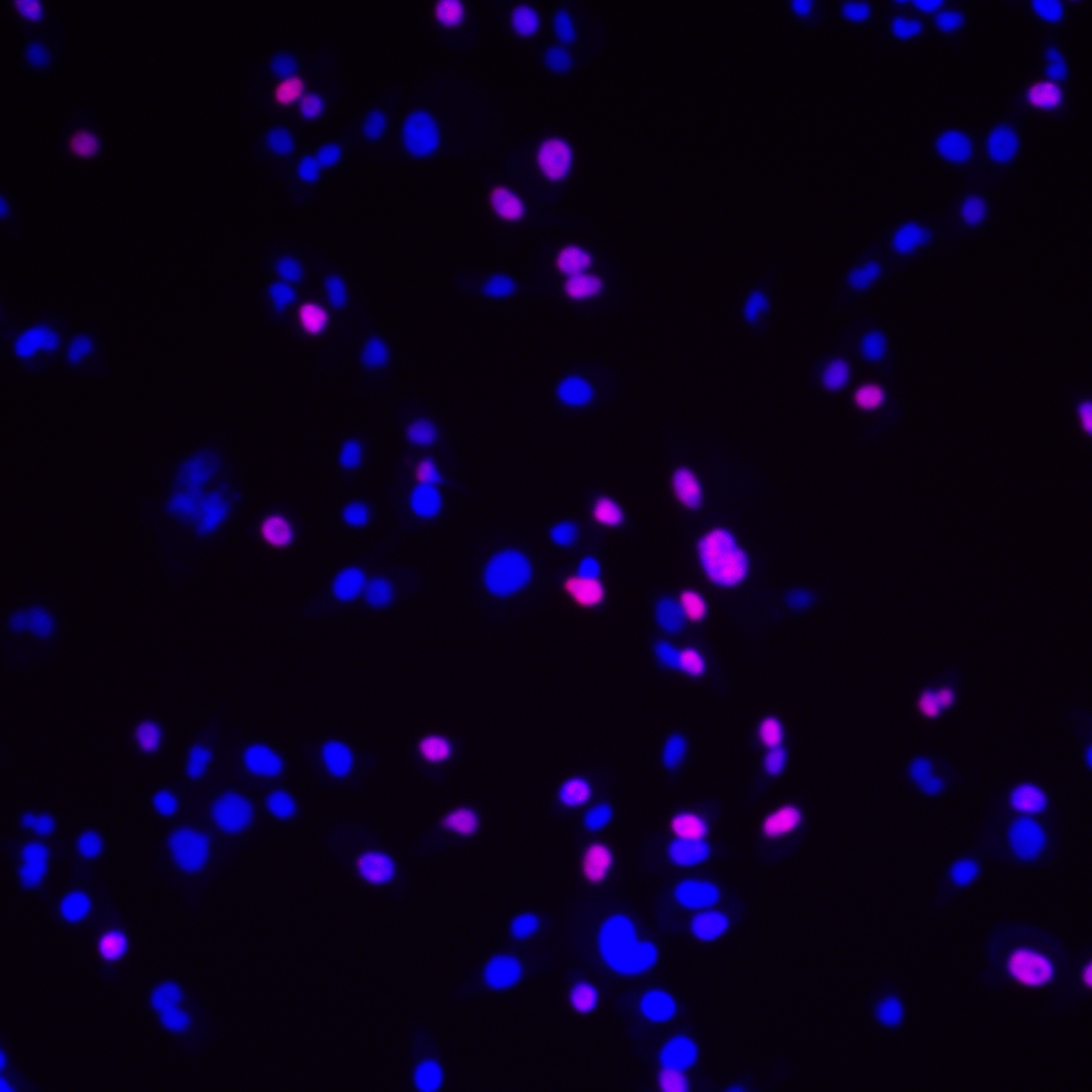

Supplement: Figure 2—source data 1. [file elife-68481-fig2-data1.zip › Figure 2-source data 1/2CDE source data/EdU/NC-24h.tif]

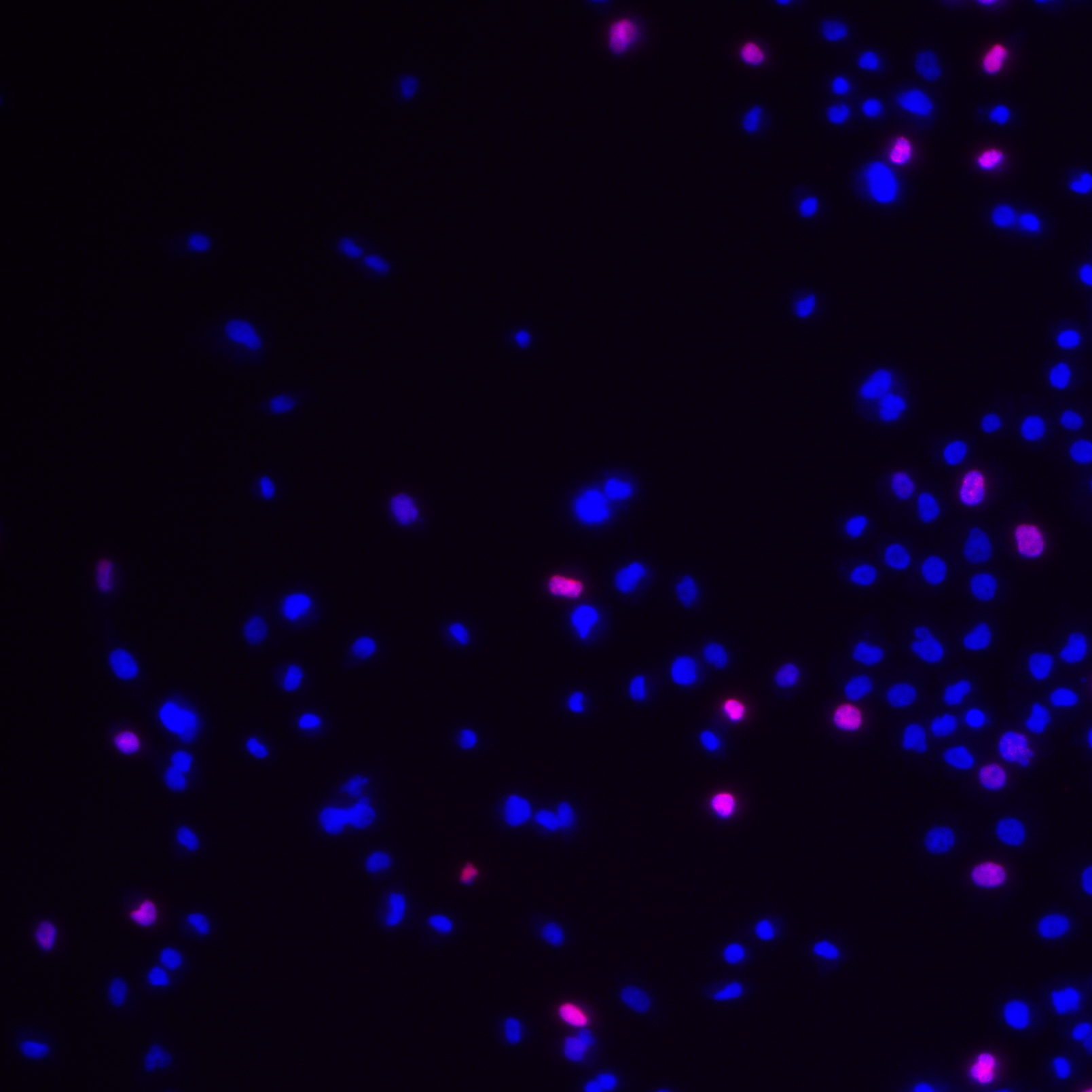

Supplement: Figure 2—source data 1. [file elife-68481-fig2-data1.zip › Figure 2-source data 1/2CDE source data/EdU/NC-48h.tif]

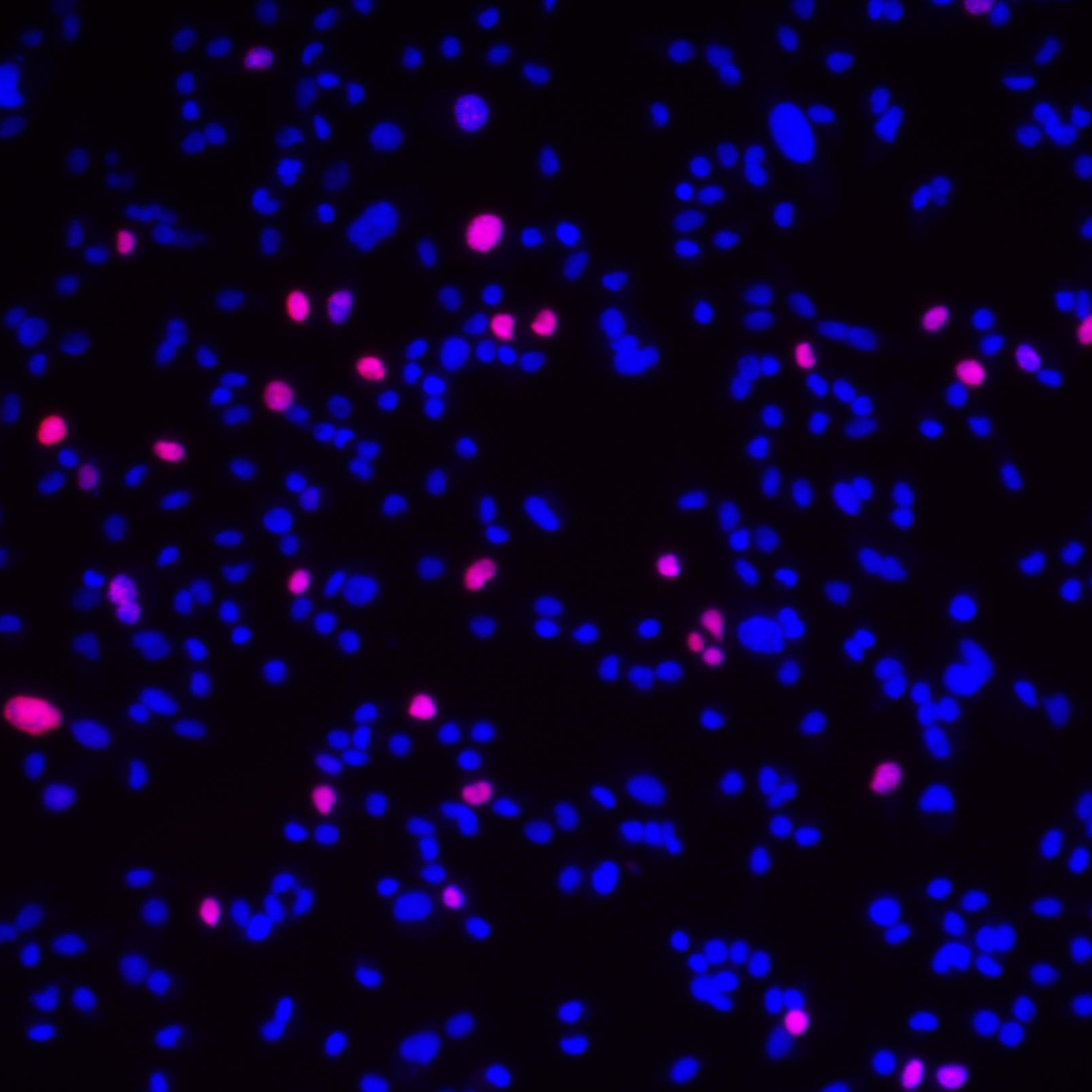

Supplement: Figure 2—source data 1. [file elife-68481-fig2-data1.zip › Figure 2-source data 1/2CDE source data/EdU/NC-72h.tif]

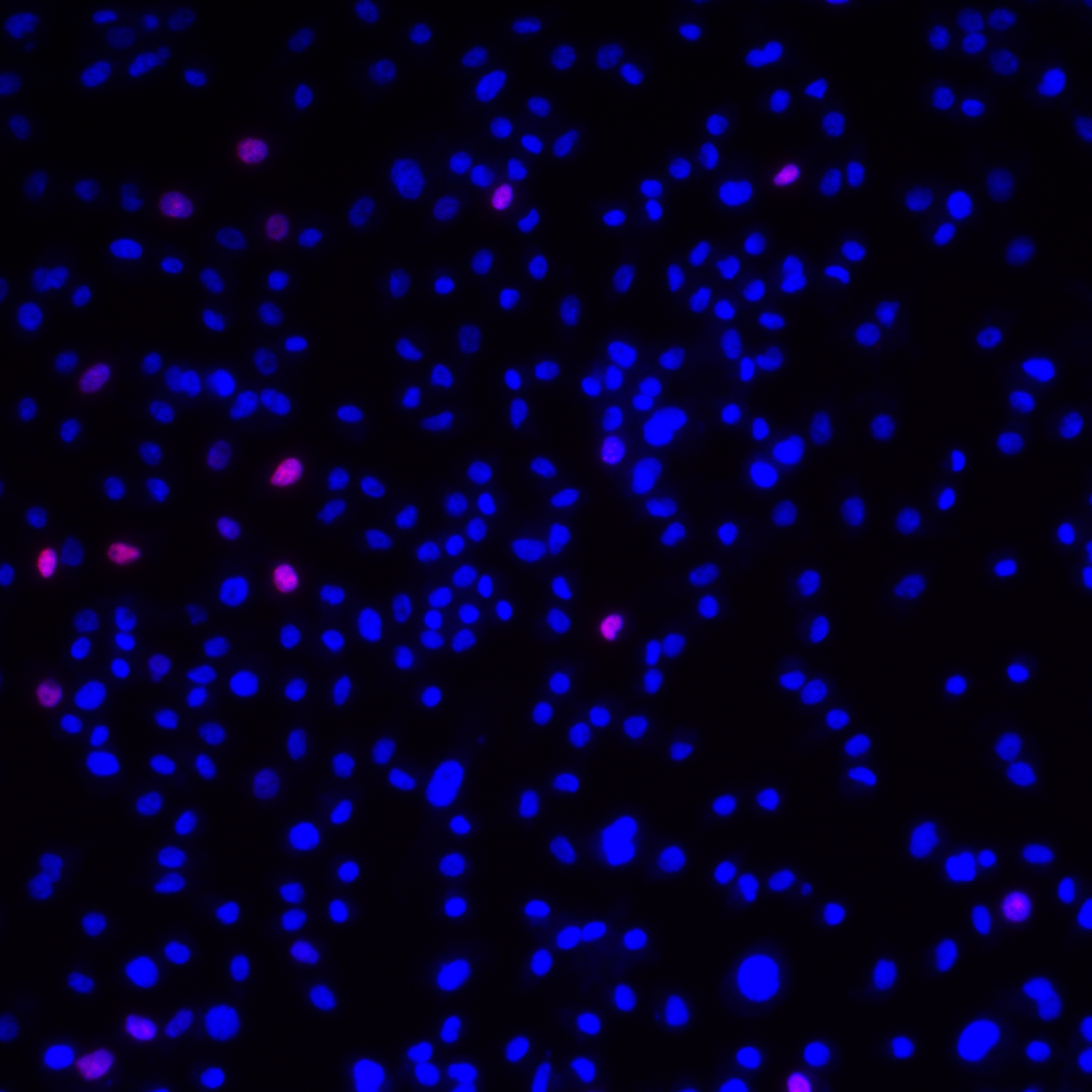

Supplement: Figure 2—source data 1. [file elife-68481-fig2-data1.zip › Figure 2-source data 1/2CDE source data/EdU/shTLN1-72h.tif]

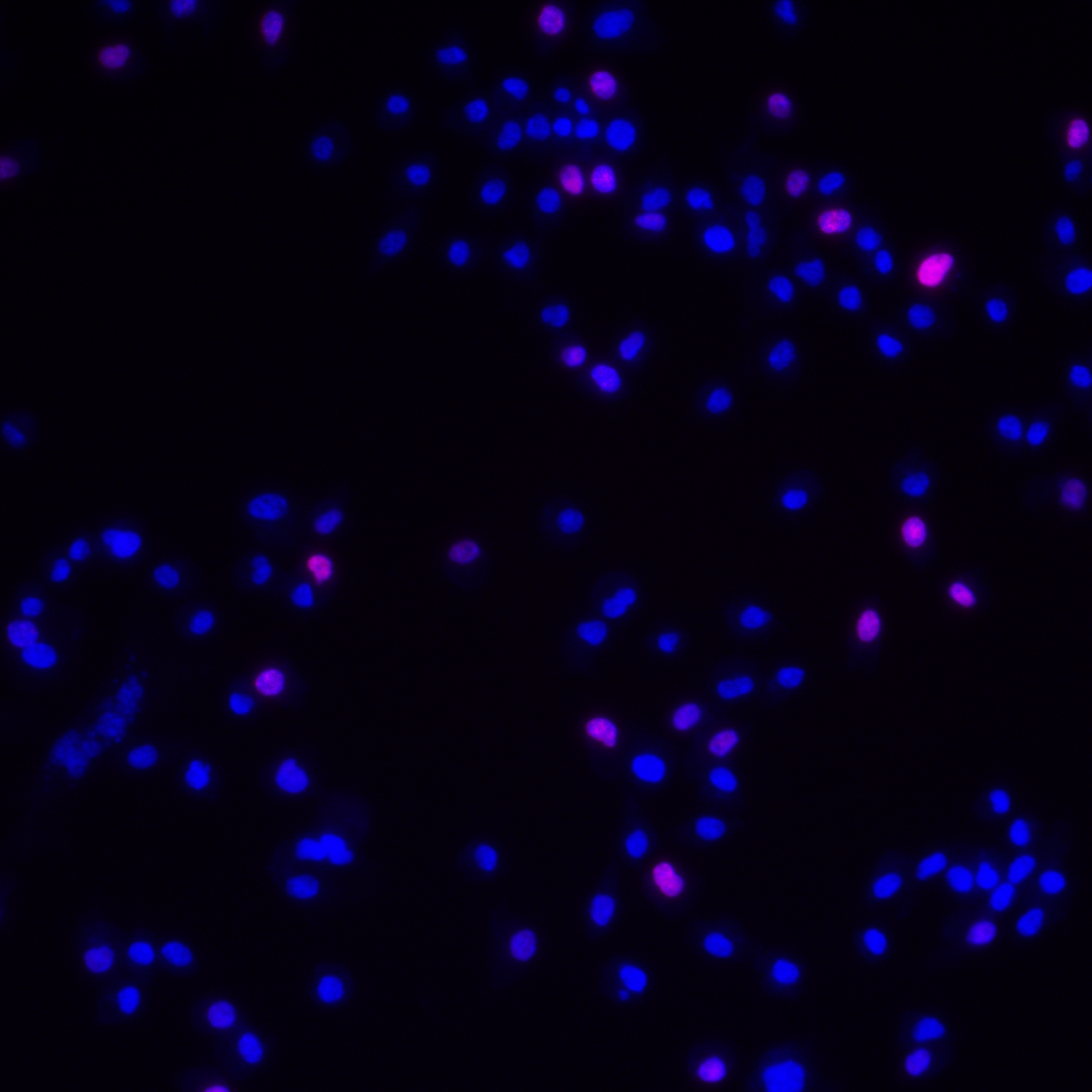

Supplement: Figure 2—source data 1. [file elife-68481-fig2-data1.zip › Figure 2-source data 1/2CDE source data/EdU/shTLN1-48h.tif]

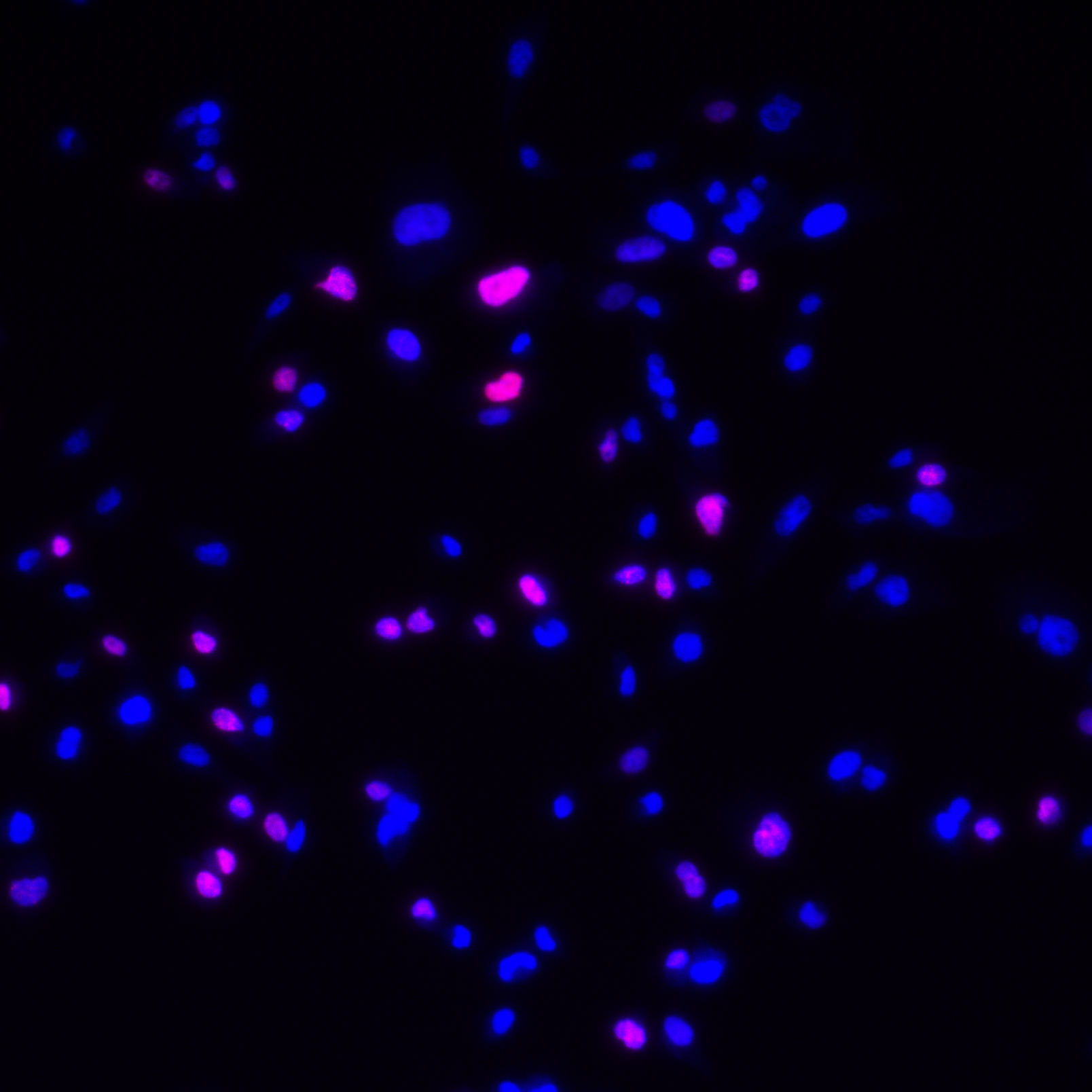

Supplement: Figure 2—source data 1. [file elife-68481-fig2-data1.zip › Figure 2-source data 1/2CDE source data/EdU/shTLN1-24h.tif]

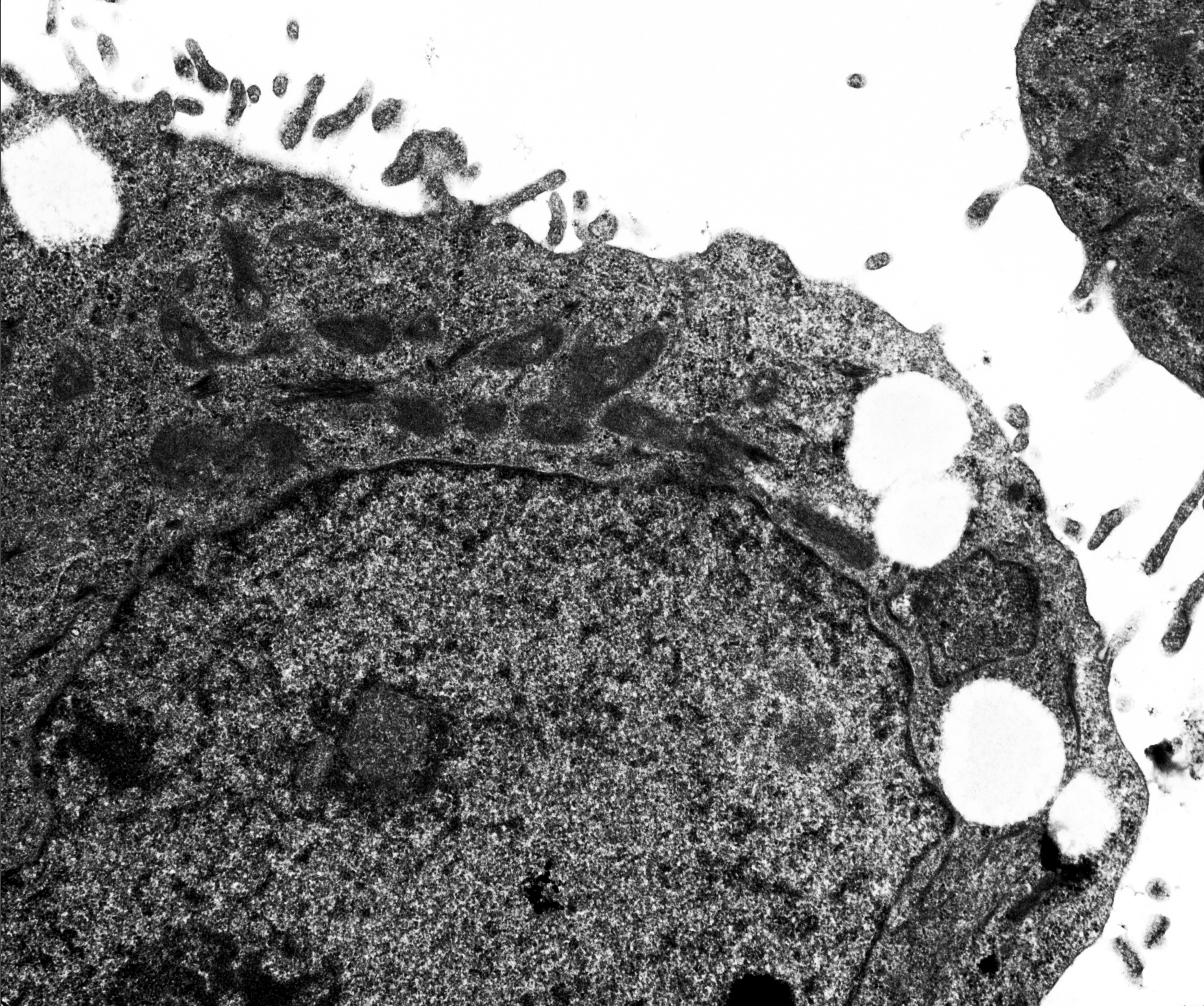

Supplement: Figure 2—source data 1. [file elife-68481-fig2-data1.zip › Figure 2-source data 1/2CDE source data/TEM/shTLN1-max.jpg]

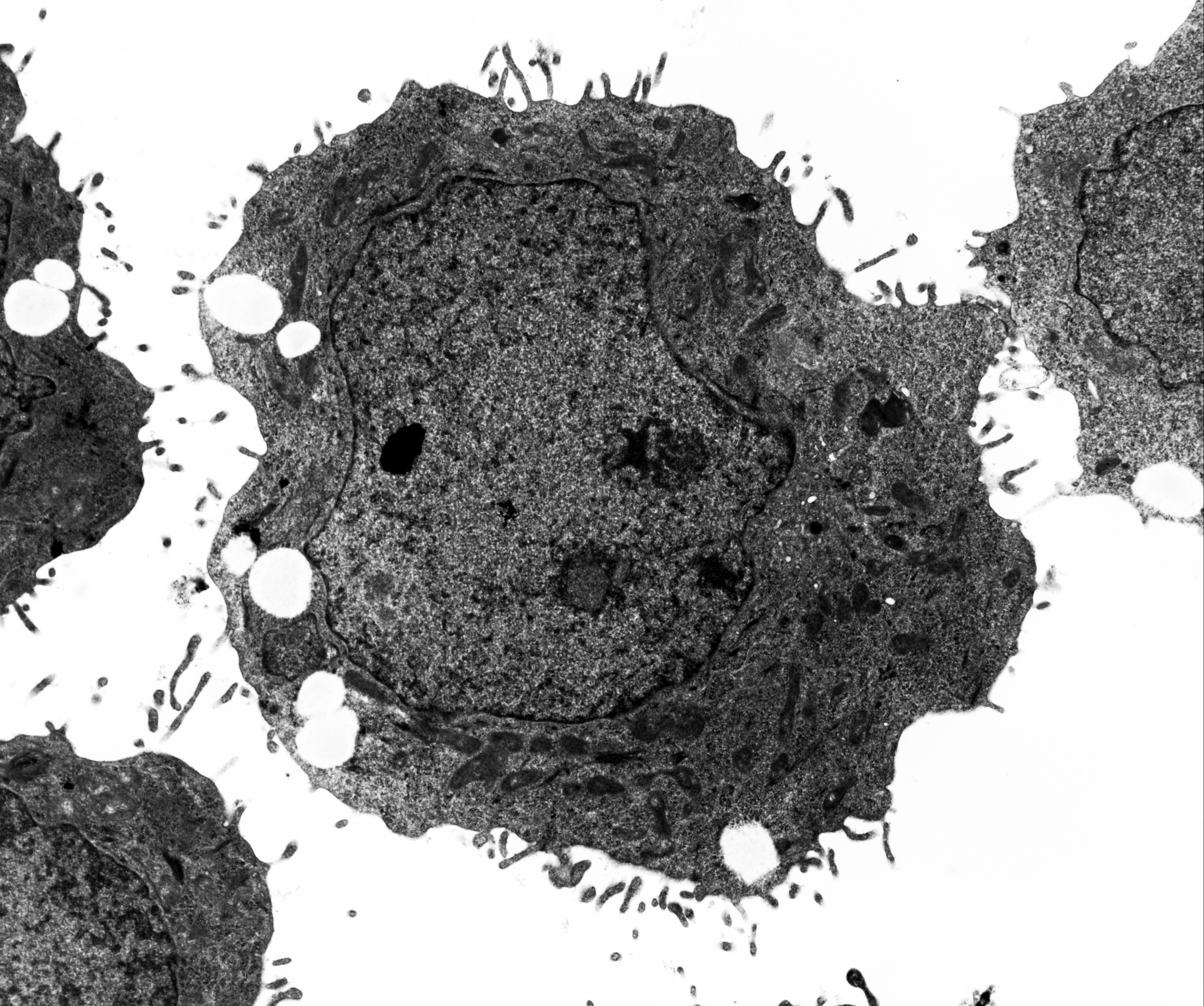

Supplement: Figure 2—source data 1. [file elife-68481-fig2-data1.zip › Figure 2-source data 1/2CDE source data/TEM/shTLN1.jpg]

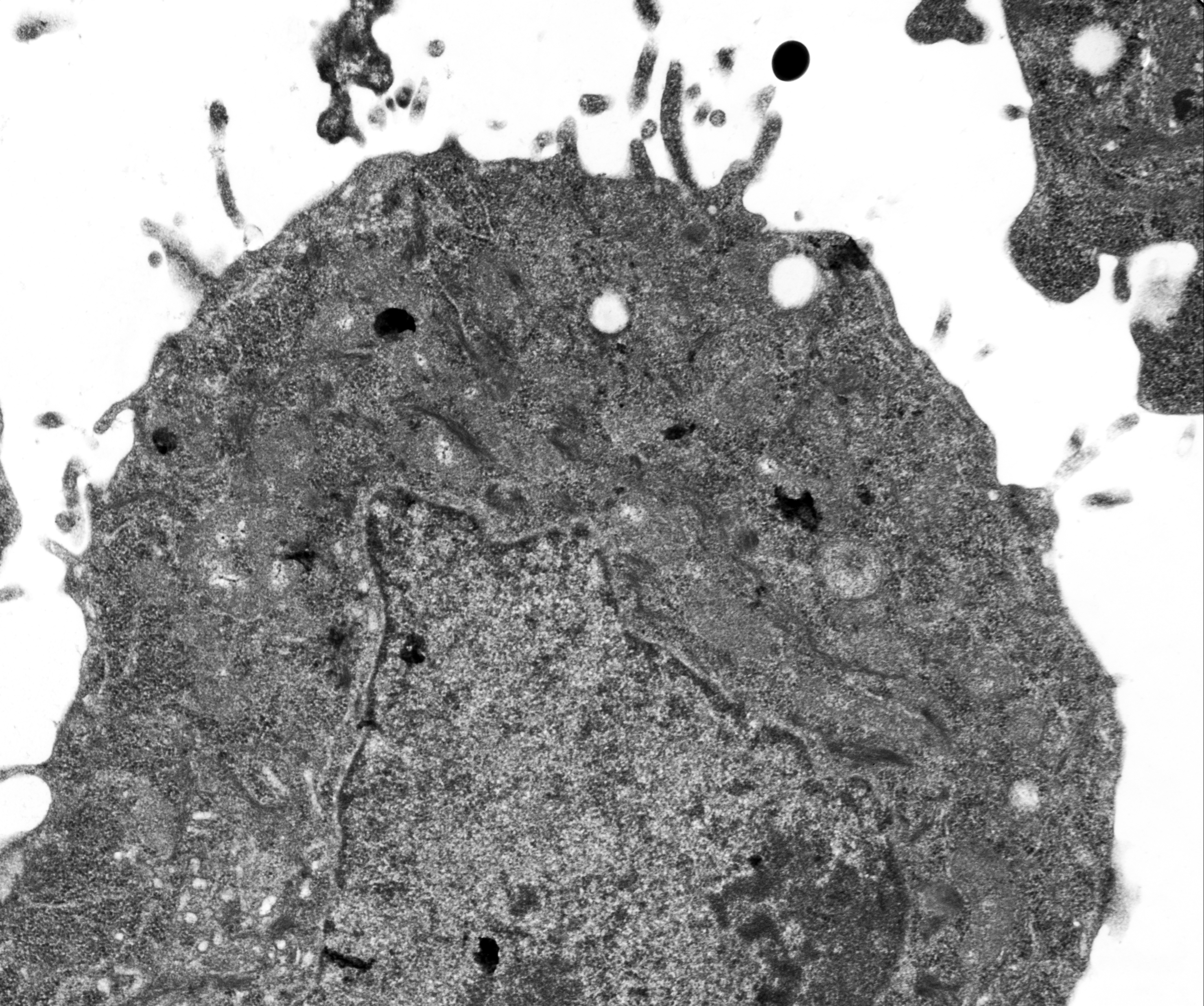

Supplement: Figure 2—source data 1. [file elife-68481-fig2-data1.zip › Figure 2-source data 1/2CDE source data/TEM/NC-max.jpg]

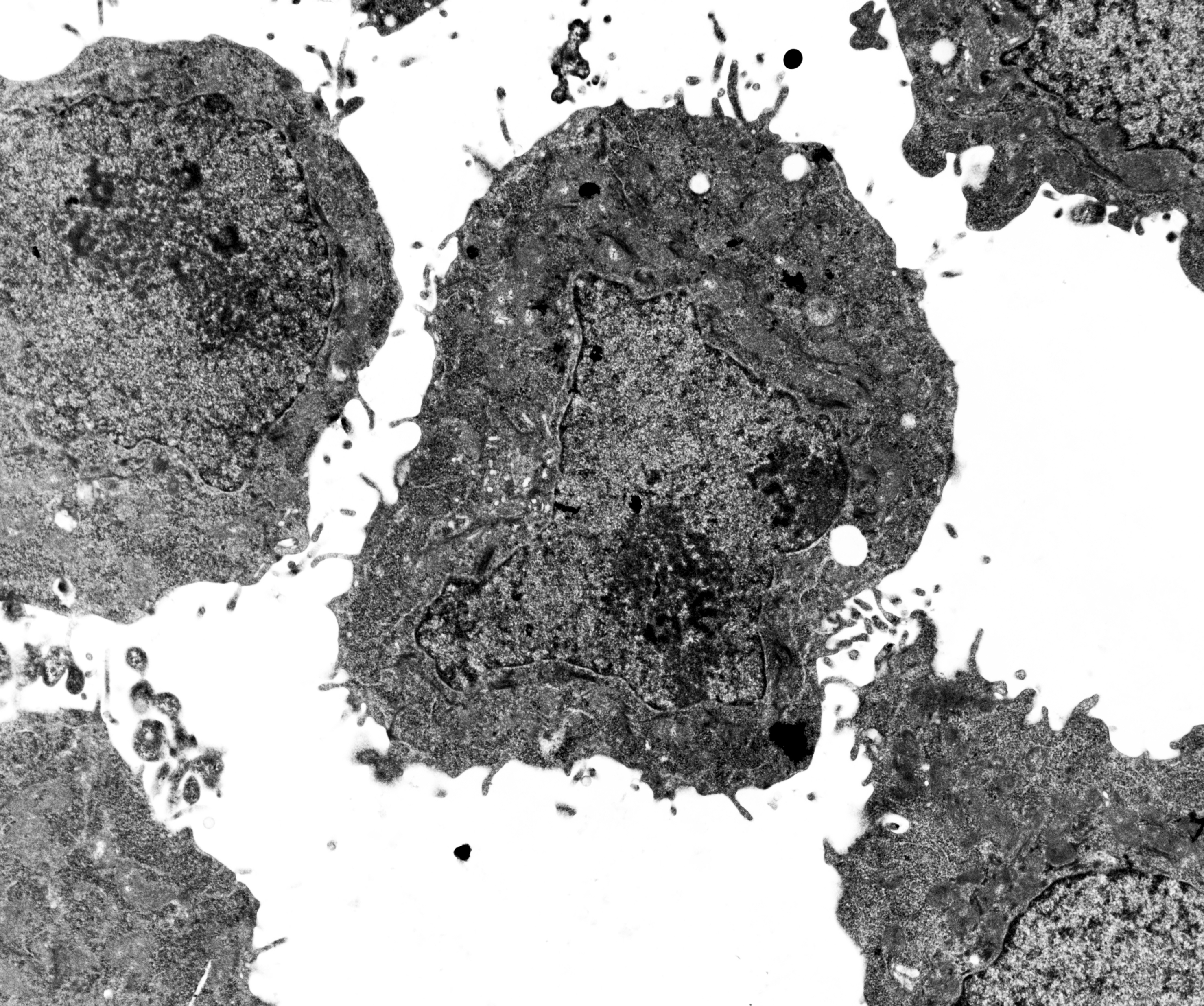

Supplement: Figure 2—source data 1. [file elife-68481-fig2-data1.zip › Figure 2-source data 1/2CDE source data/TEM/NC.jpg]

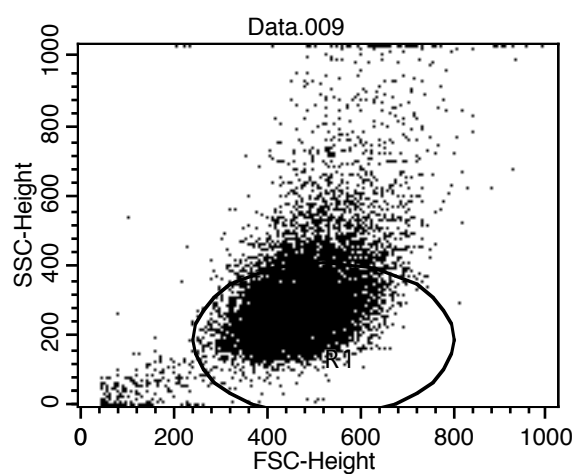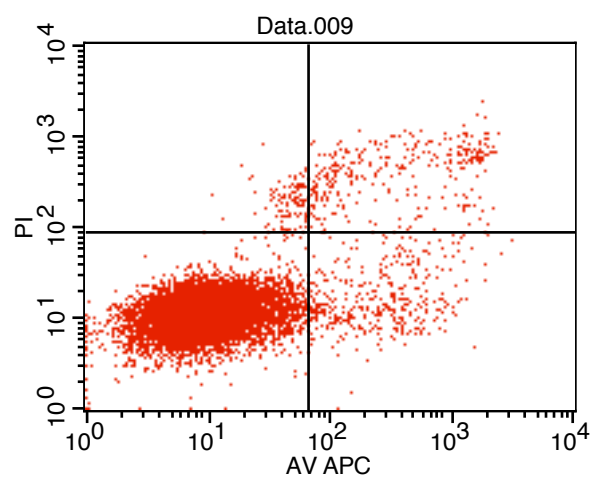

| Quad | Events | % Gated | % Total | X Mean | Y Mean |
|------|--------|---------|---------|--------|--------|
| UL   | 104    | 1.13    | 0.92    | 47.35  | 210.47 |
| UR   | 323    | 3.51    | 2.86    | 653.67 | 489.26 |
| LL   | 8386   | 91.15   | 74.26   | 12.01  | 12.09  |
| LR   | 387    | 4.21    | 3.43    | 351.38 | 20.92  |

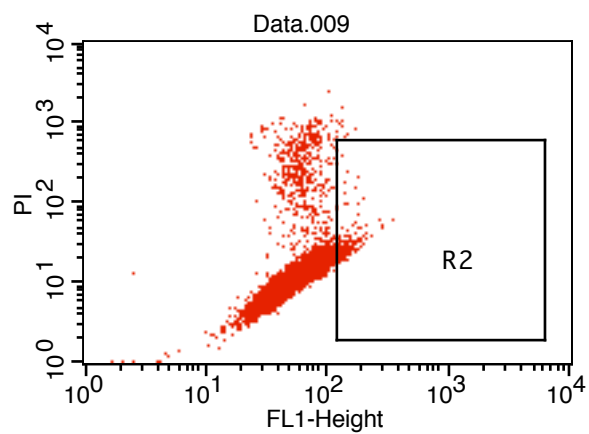

| Region | Events | % Gated | % Total | X Mean | Y Mean |
|--------|--------|---------|---------|--------|--------|
| R1     | 9200   | 100.00  | 81.47   | 58.49  | 31.46  |
| R2     | 195    | 2.12    | 1.73    | 149.01 | 38.13  |

Supplement: Figure 2—source data 1. [file elife-68481-fig2-data1.zip › Figure 2-source data 1/2CDE source data/apoptosis/shTLN1.pdf]

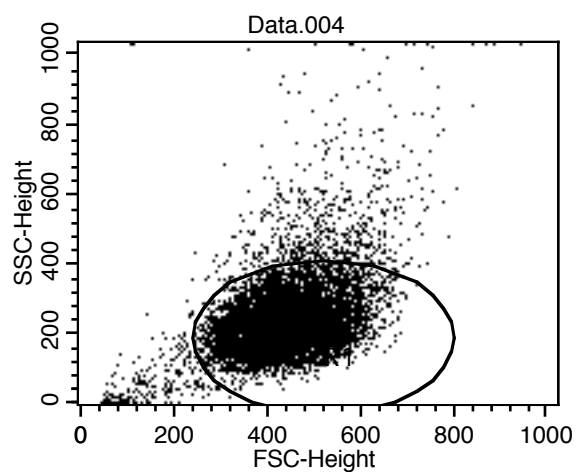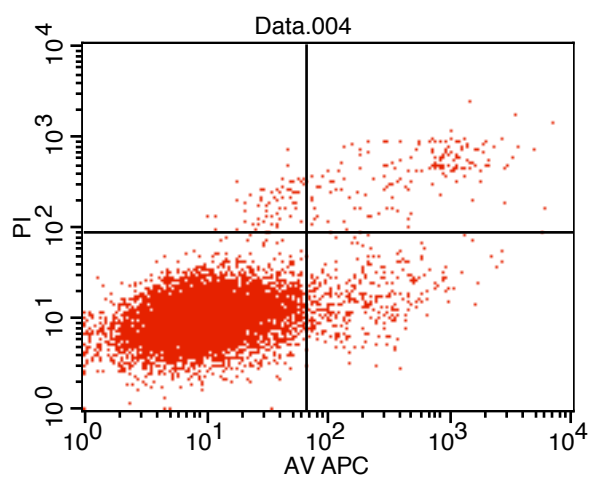

| Quad | Events | % Gated | % Total | X Mean | Y Mean |
|------|--------|---------|---------|--------|--------|
| UL   | 71     | 0.73    | 0.67    | 37.69  | 203.76 |
| UR   | 164    | 1.69    | 1.55    | 913.98 | 499.93 |
| LL   | 9126   | 94.19   | 86.25   | 12.13  | 11.22  |
| LR   | 328    | 3.39    | 3.10    | 297.70 | 19.88  |

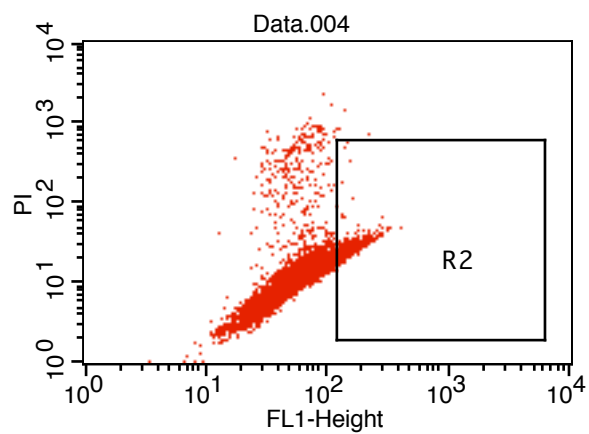

| Region | Events | % Gated | % Total | X Mean | Y Mean |
|--------|--------|---------|---------|--------|--------|
| R1     | 9689   | 100.00  | 91.57   | 58.70  | 21.20  |
| R2     | 495    | 5.11    | 4.68    | 156.69 | 29.94  |

Supplement: Figure 2—source data 1. [file elife-68481-fig2-data1.zip › Figure 2-source data 1/2CDE source data/apoptosis/NC.pdf]

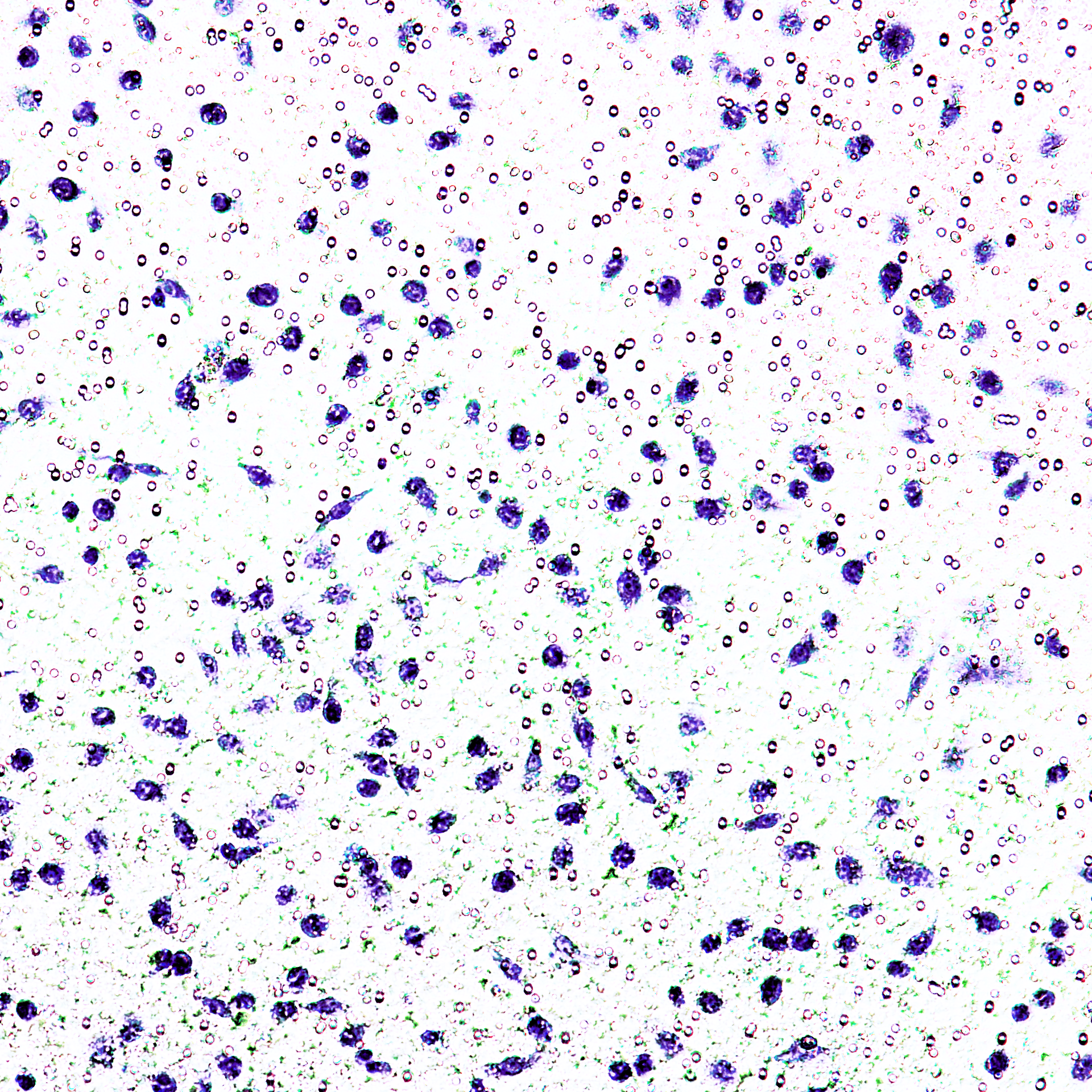

Supplement: Figure 3—source data 1. [file elife-68481-fig3-data1.zip › Figure 3-source data 1/3B source data/Migration shTALIN1.tif]

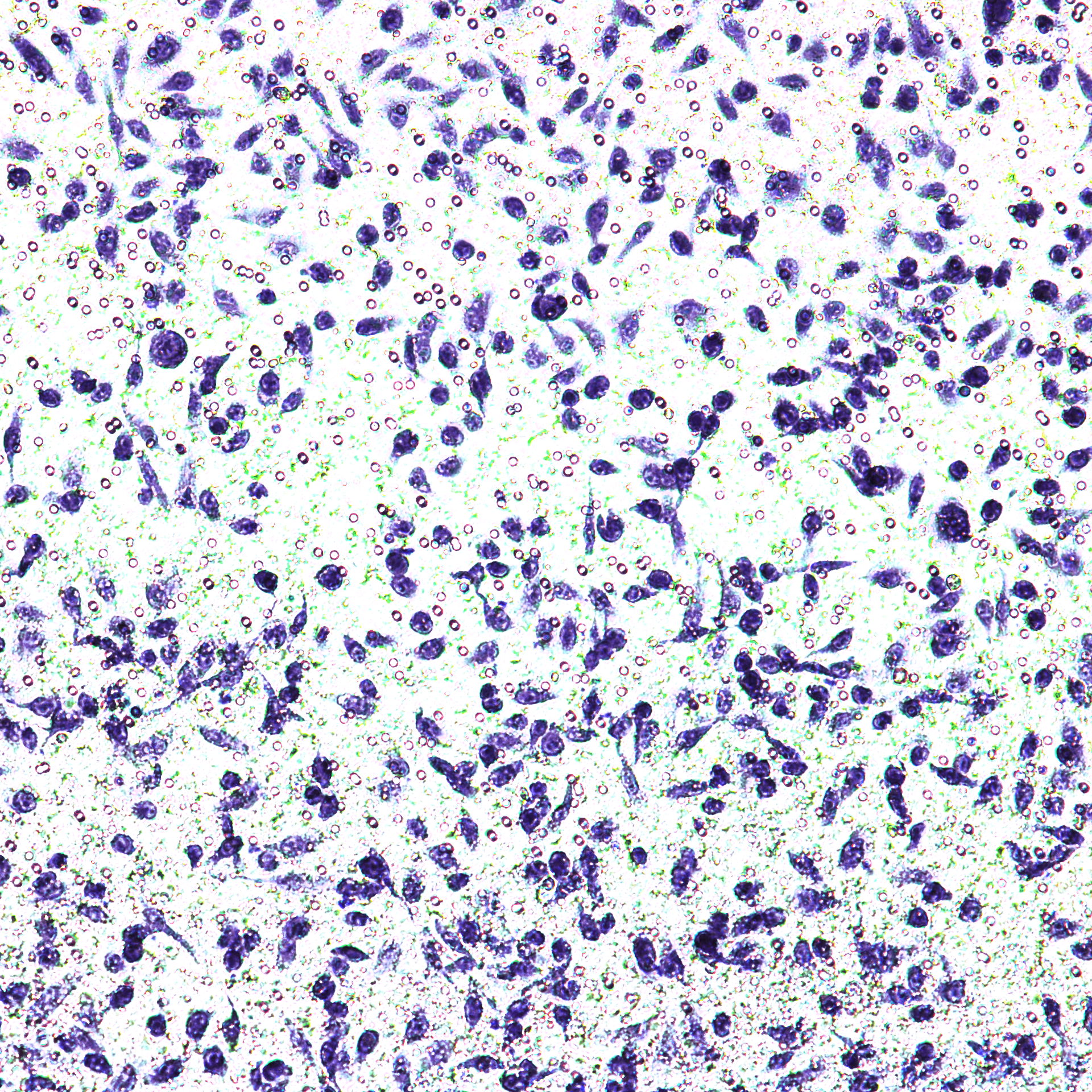

Supplement: Figure 3—source data 1. [file elife-68481-fig3-data1.zip › Figure 3-source data 1/3B source data/Migration NC.tif]

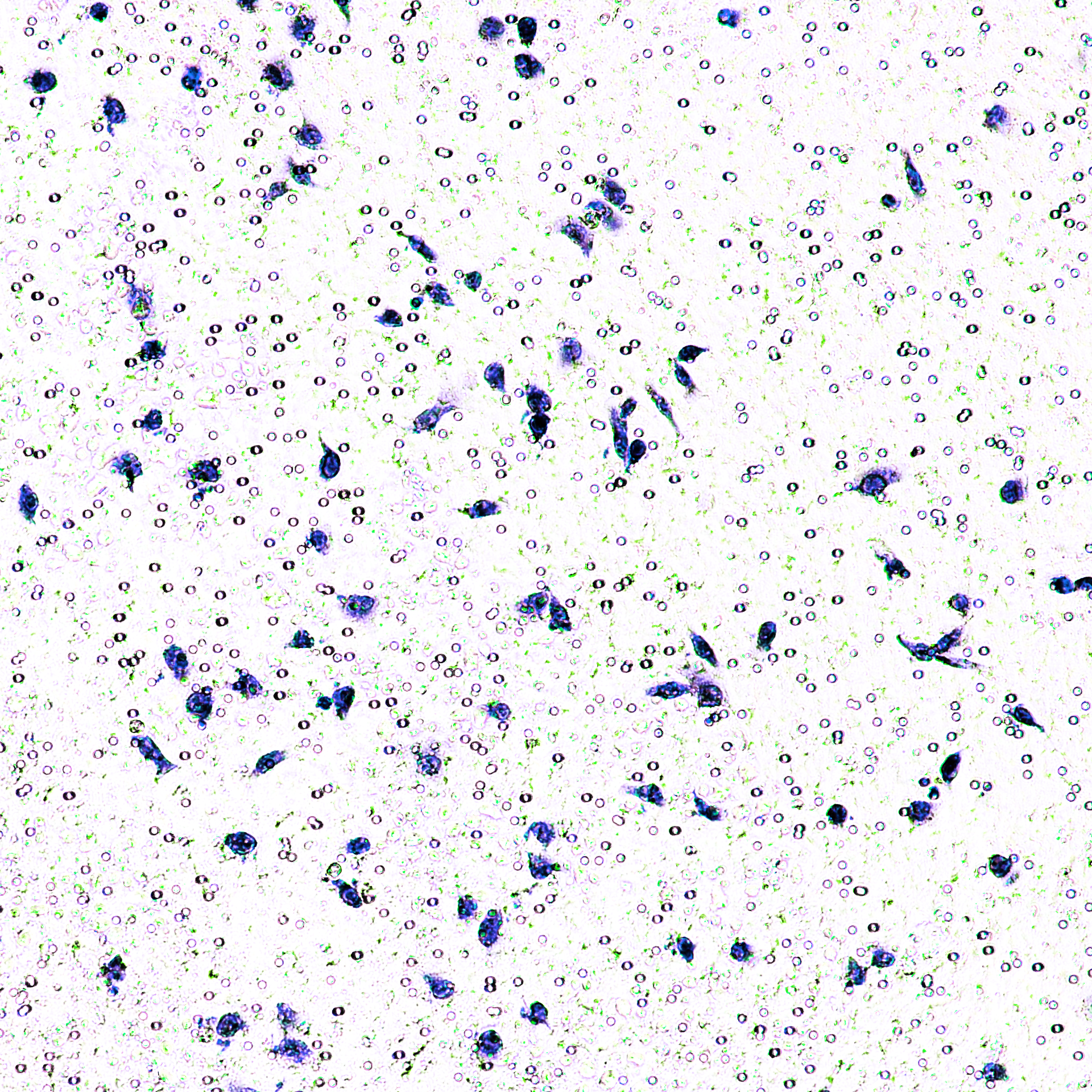

Supplement: Figure 3—source data 1. [file elife-68481-fig3-data1.zip › Figure 3-source data 1/3B source data/Invasion shTALIN1.tif]

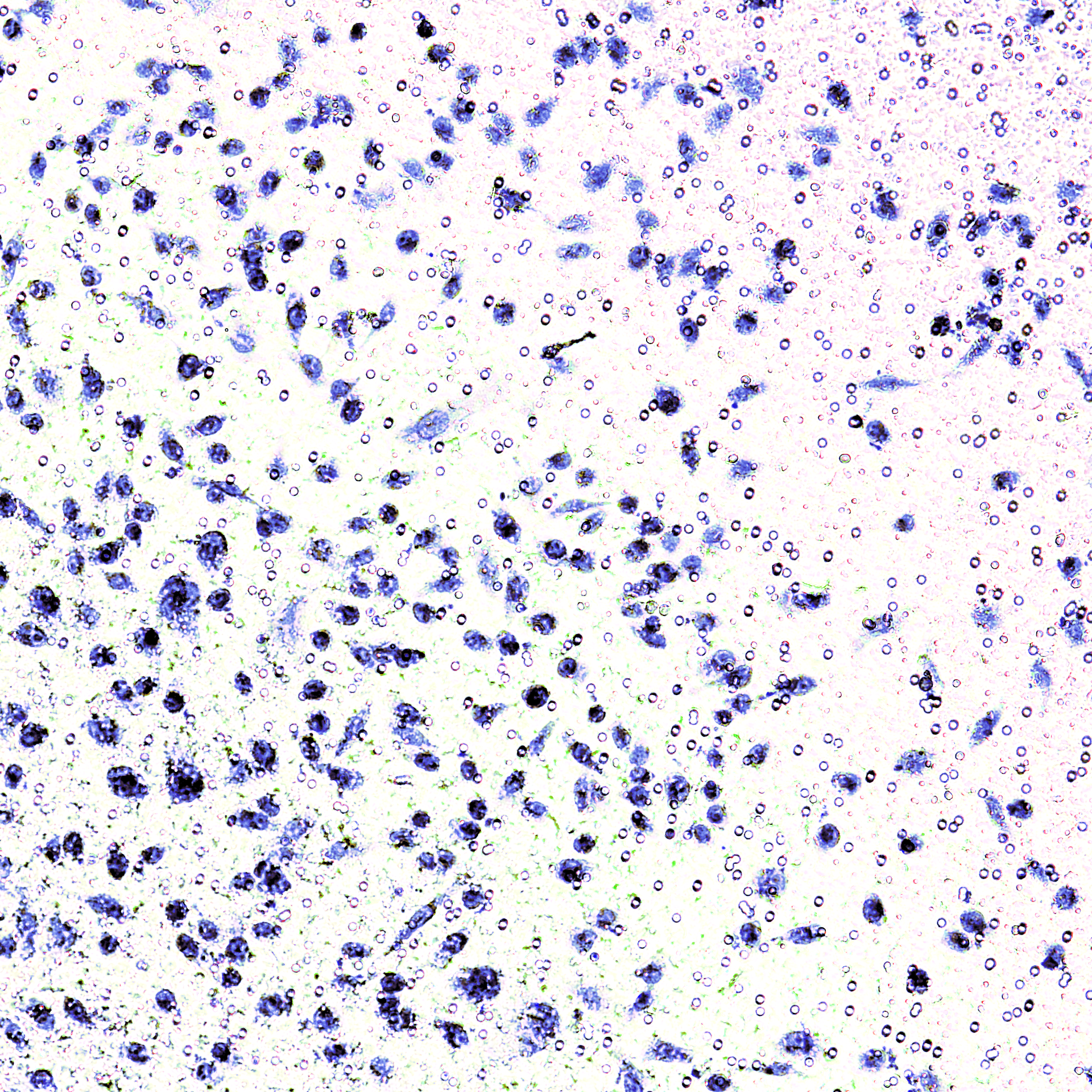

Supplement: Figure 3—source data 1. [file elife-68481-fig3-data1.zip › Figure 3-source data 1/3B source data/Invasion NC.tif]

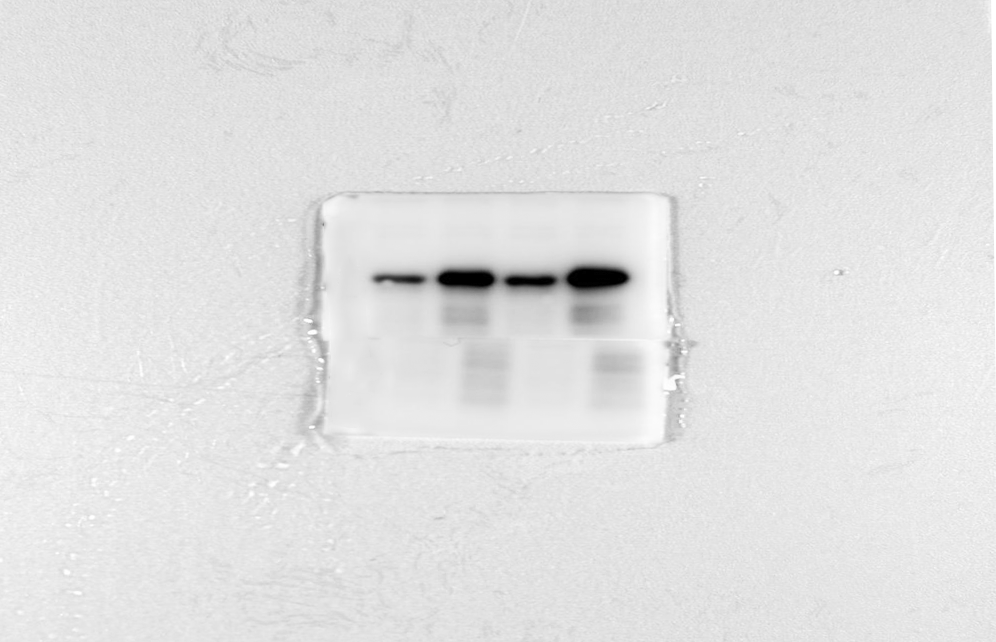

Supplement: Figure 3—source data 1. [file elife-68481-fig3-data1.zip › Figure 3-source data 1/3FGH source data/integerin b1.tif]

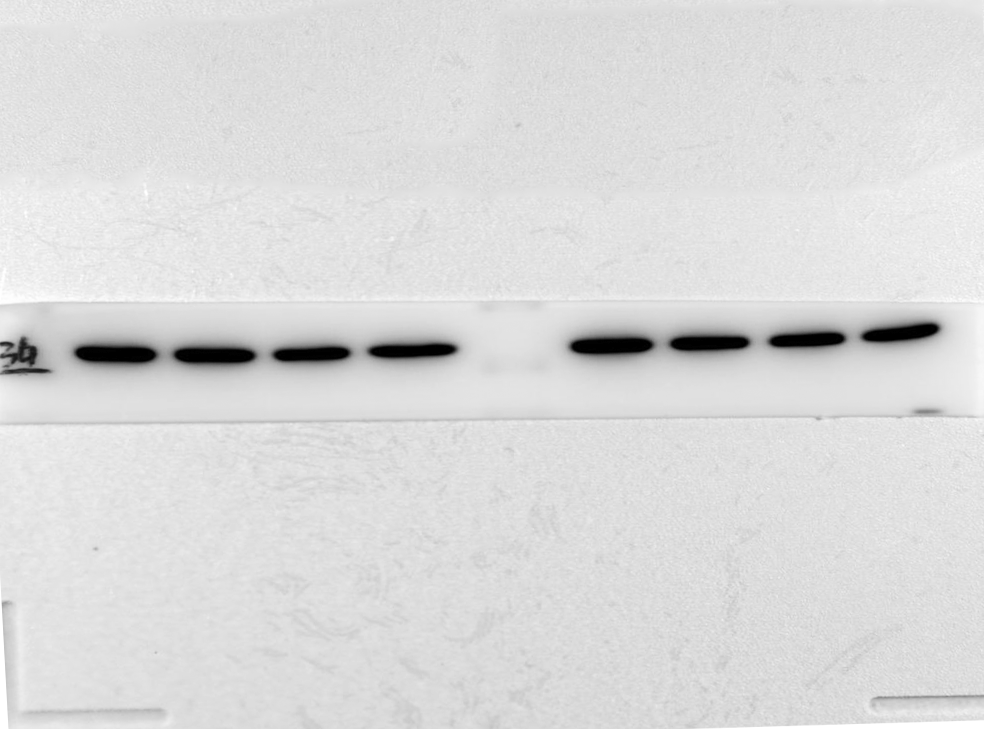

Supplement: Figure 3—source data 1. [file elife-68481-fig3-data1.zip › Figure 3-source data 1/3FGH source data/gapdh.tif]

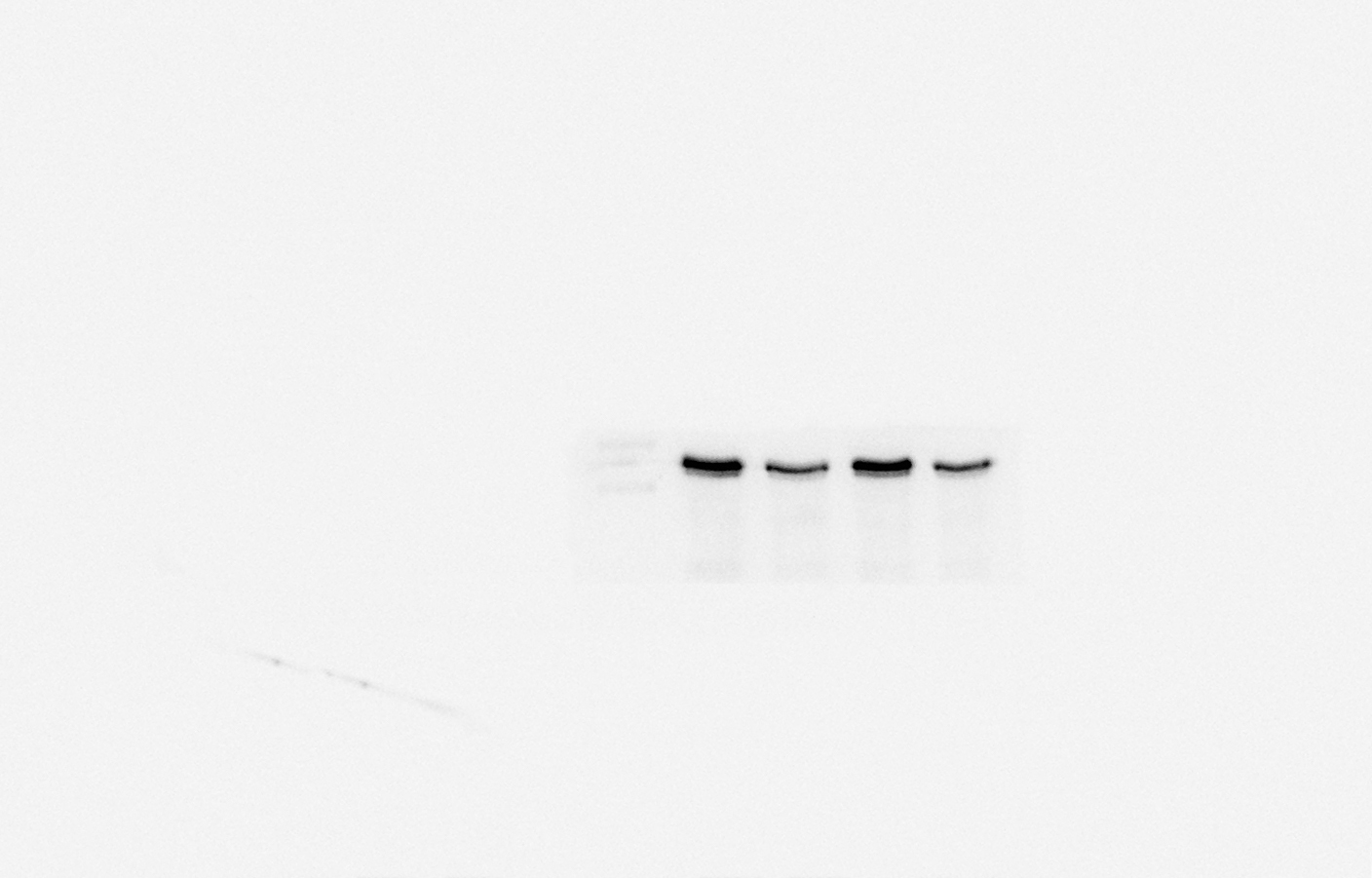

Supplement: Figure 3—source data 1. [file elife-68481-fig3-data1.zip › Figure 3-source data 1/3CD source data/vim.jpg]

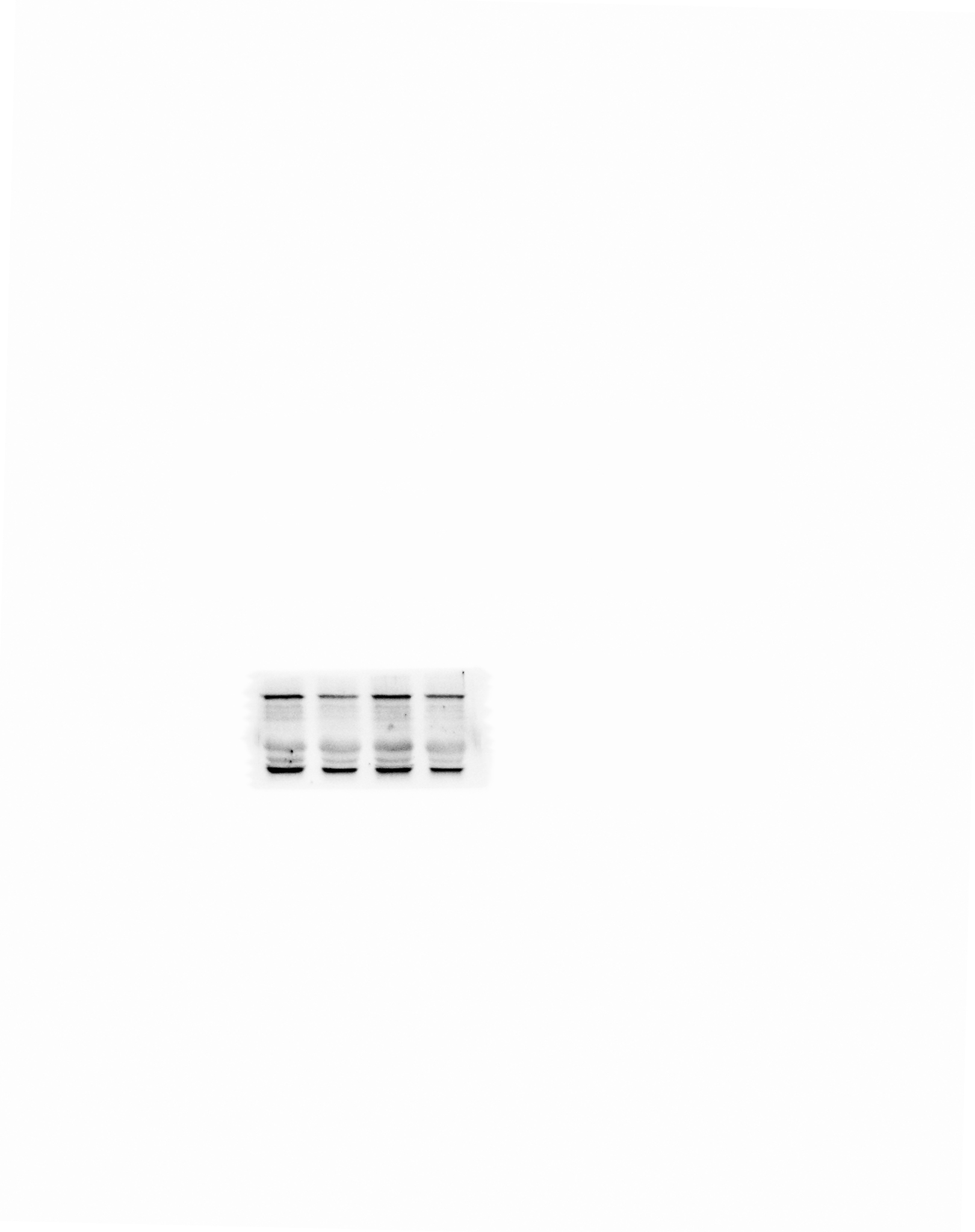

Supplement: Figure 3—source data 1. [file elife-68481-fig3-data1.zip › Figure 3-source data 1/3CD source data/CK18.jpg]

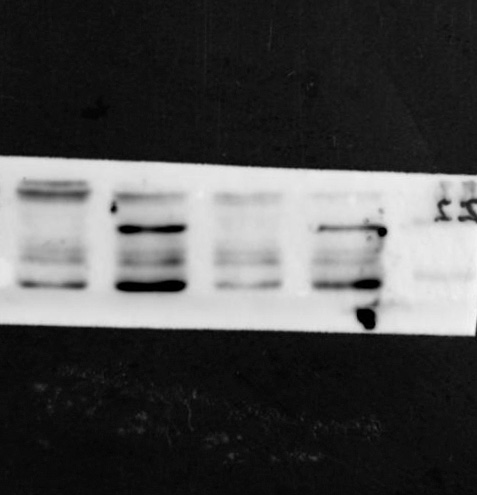

Supplement: Figure 3—source data 1. [file elife-68481-fig3-data1.zip › Figure 3-source data 1/3CD source data/E-ca.jpg]

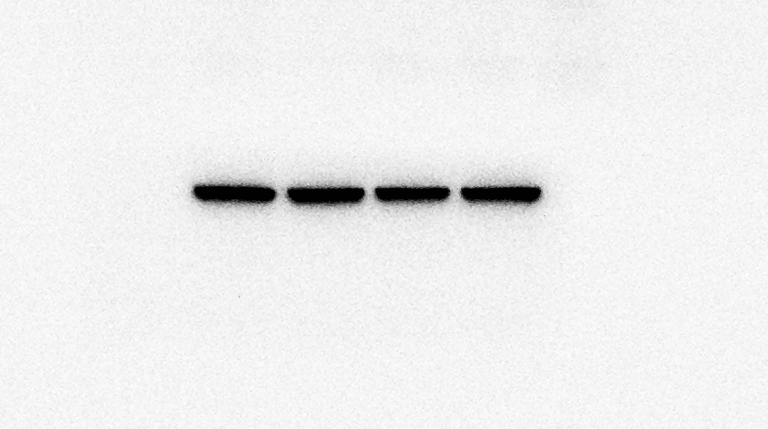

Supplement: Figure 3—source data 1. [file elife-68481-fig3-data1.zip › Figure 3-source data 1/3CD source data/GAPDH.jpg]

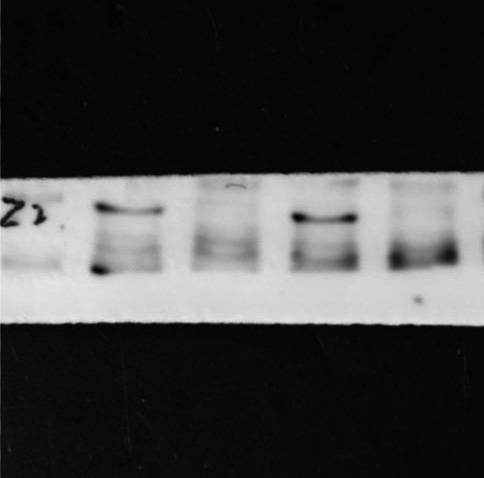

Supplement: Figure 3—source data 1. [file elife-68481-fig3-data1.zip › Figure 3-source data 1/3CD source data/N-ca.jpg]

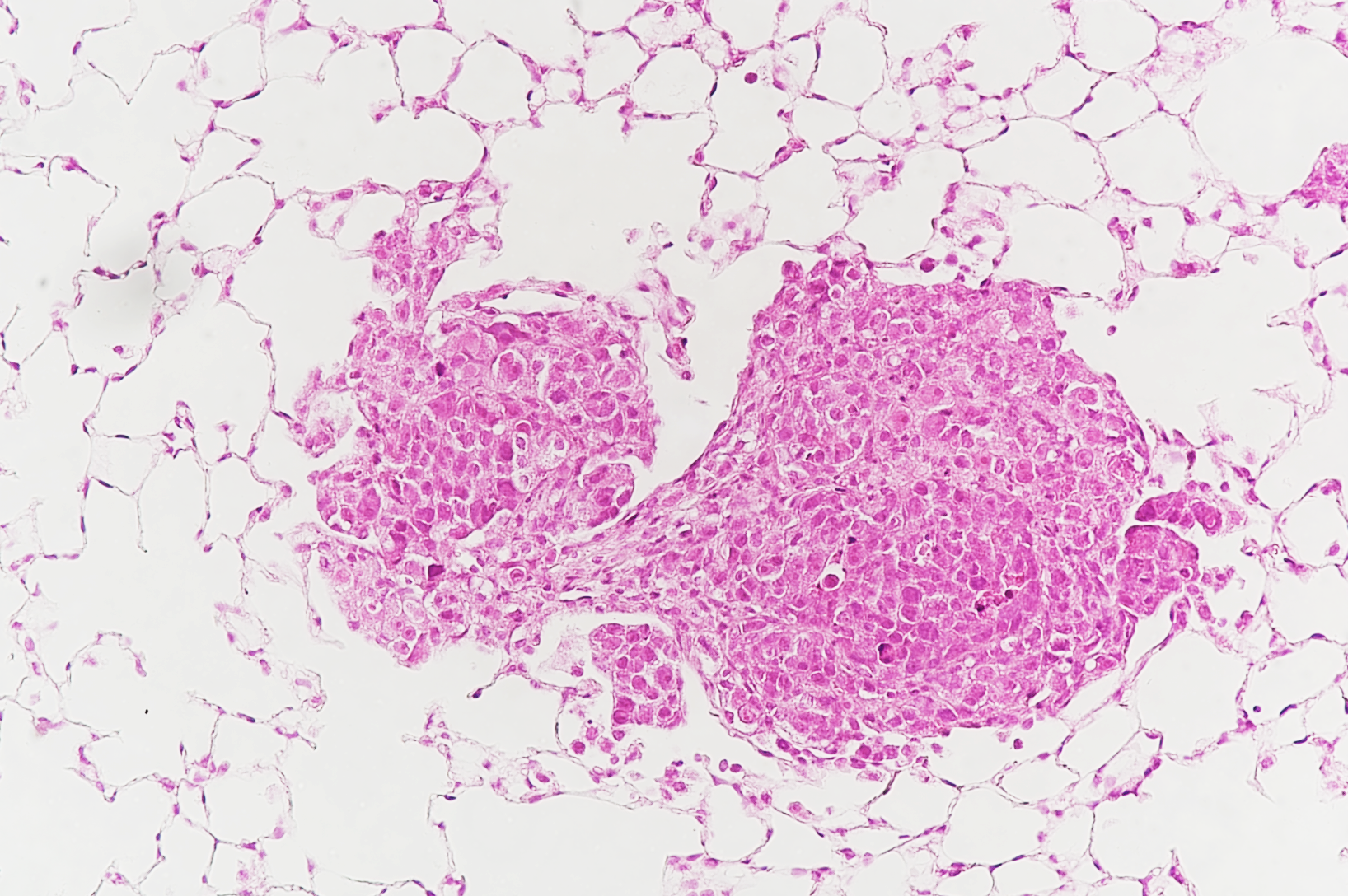

Supplement: Figure 3—source data 1. [file elife-68481-fig3-data1.zip › Figure 3-source data 1/3E source data/lung-2-2-20x-.tif]

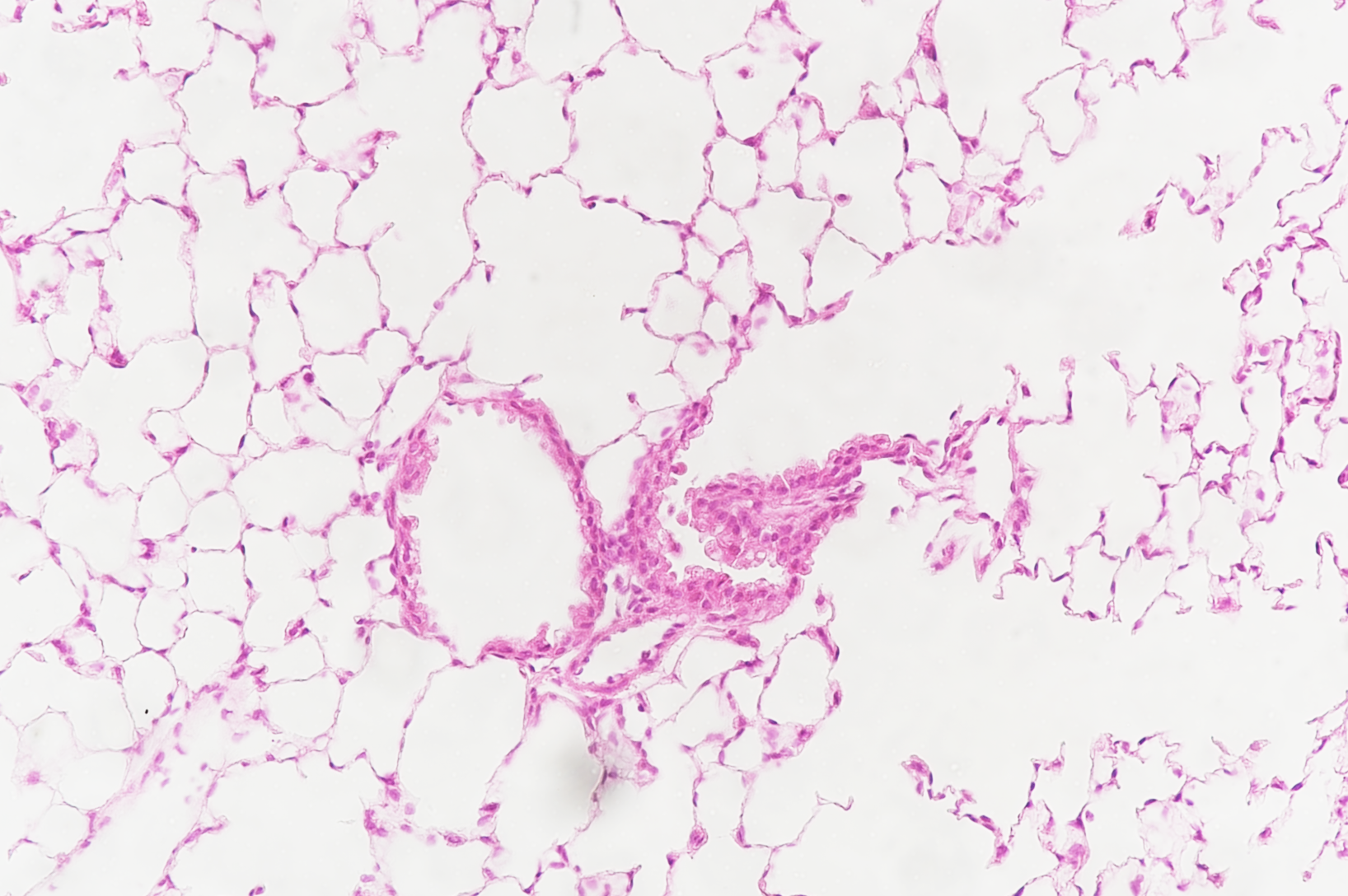

Supplement: Figure 3—source data 1. [file elife-68481-fig3-data1.zip › Figure 3-source data 1/3E source data/lung-4-3-20x-.tif]

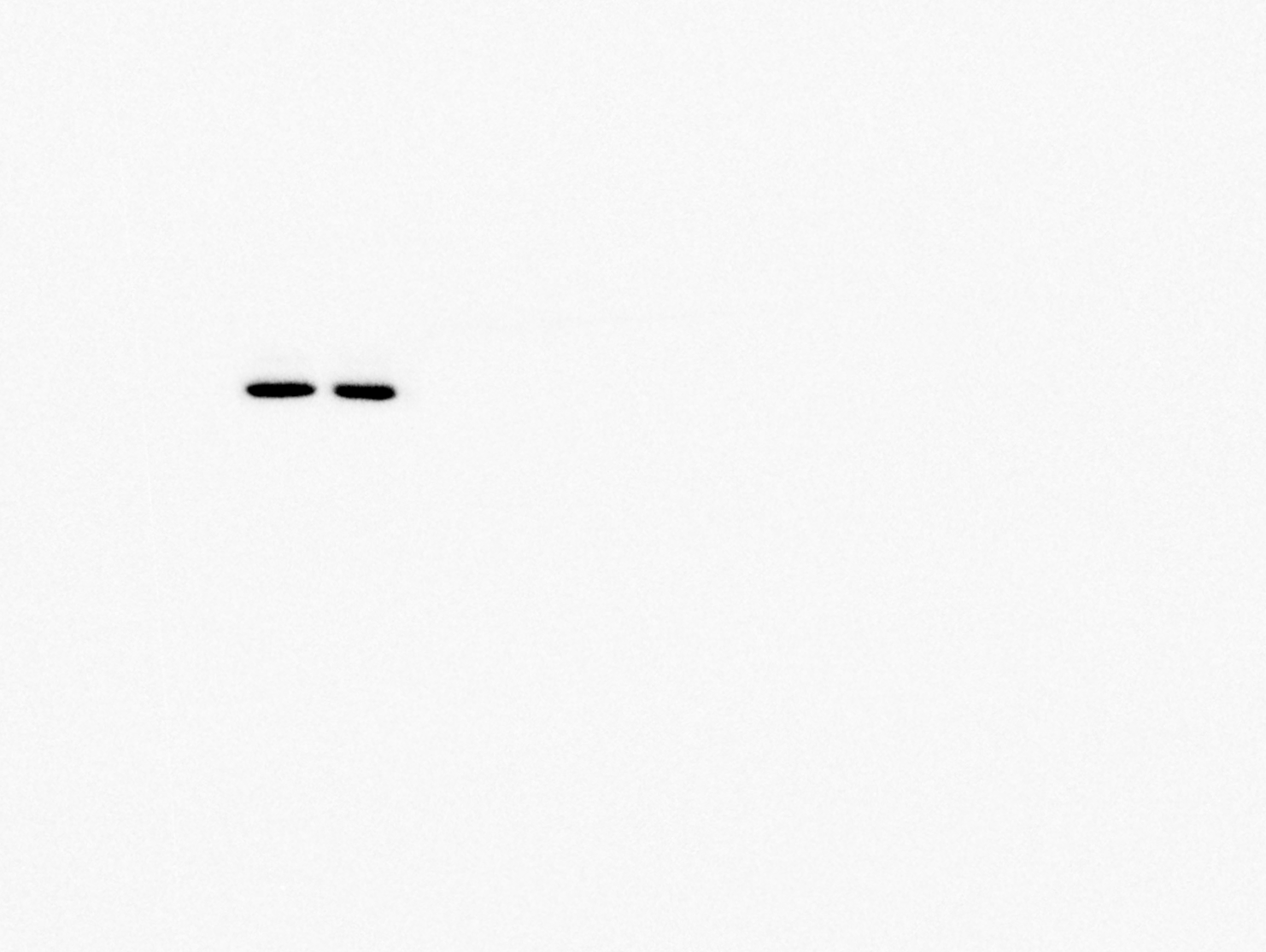

Supplement: Figure 3—figure supplement 1—source data 1. [file elife-68481-fig3-figsupp1-data1.zip › Figure 3-figure supplement 1 source data 1/S2A source data/gapdh.tif]

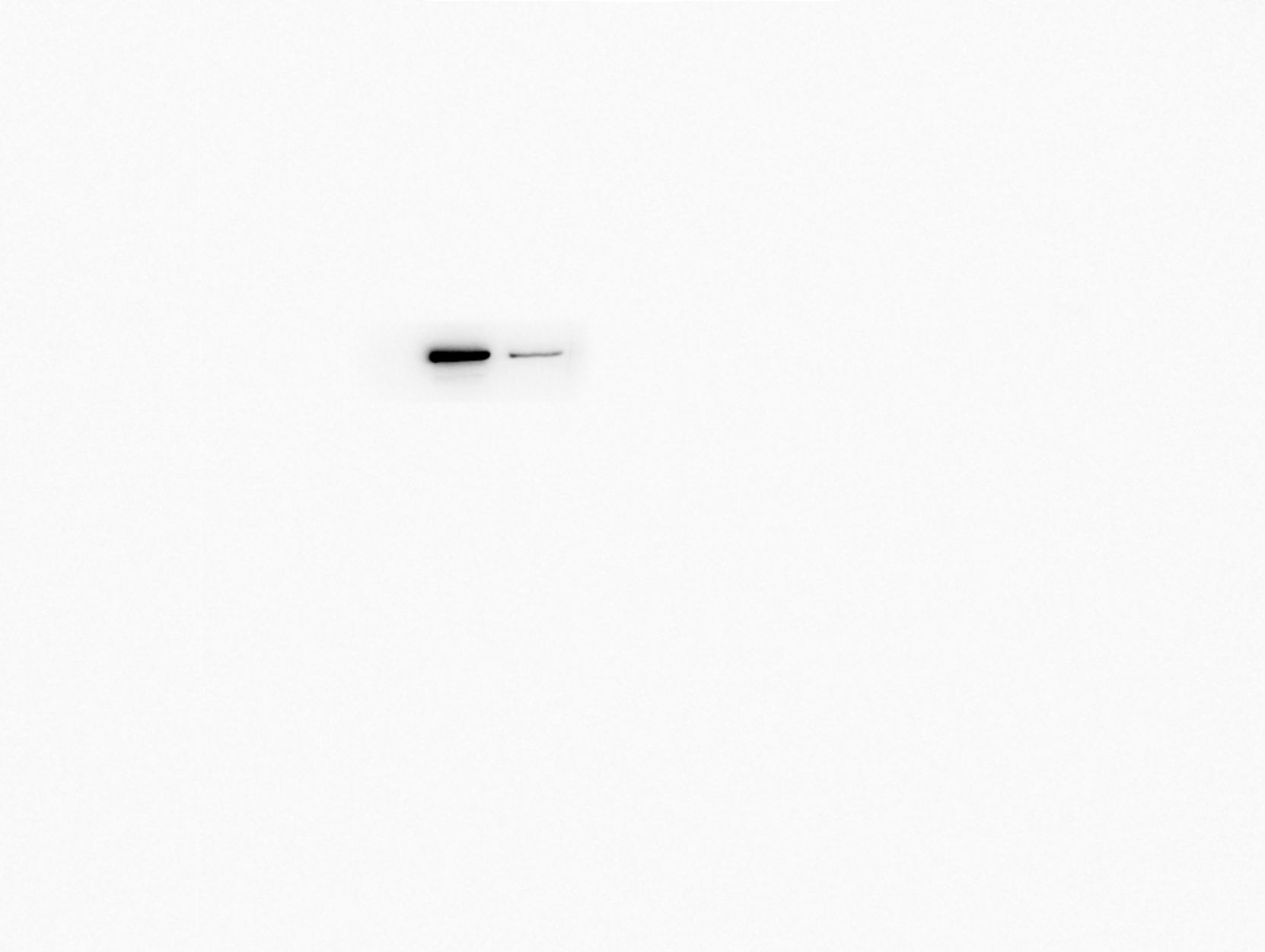

Supplement: Figure 3—figure supplement 1—source data 1. [file elife-68481-fig3-figsupp1-data1.zip › Figure 3-figure supplement 1 source data 1/S2A source data/TLN1.tif]

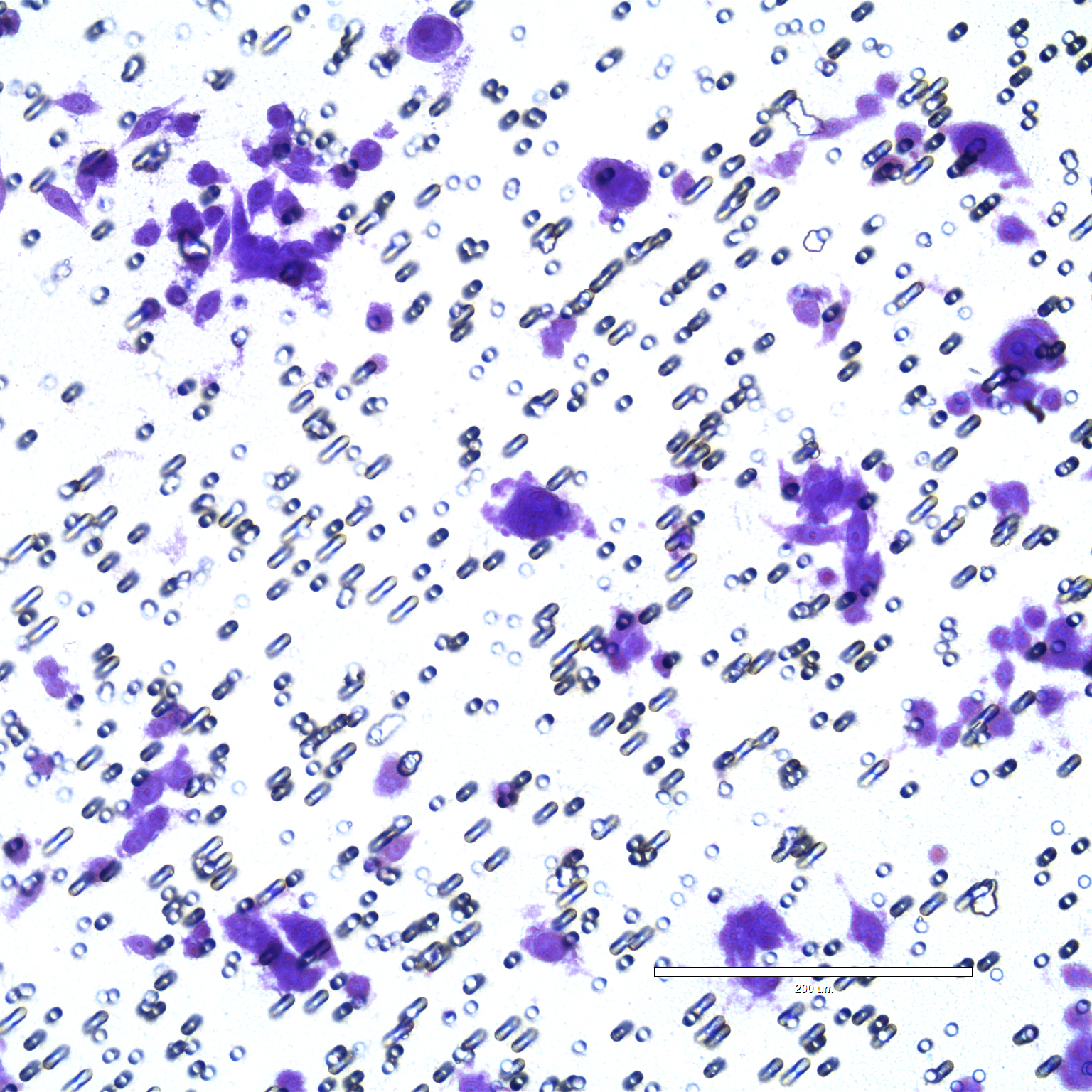

Supplement: Figure 3—figure supplement 1—source data 1. [file elife-68481-fig3-figsupp1-data1.zip › Figure 3-figure supplement 1 source data 1/S2C source data/invasion-shTLN1.jpg]

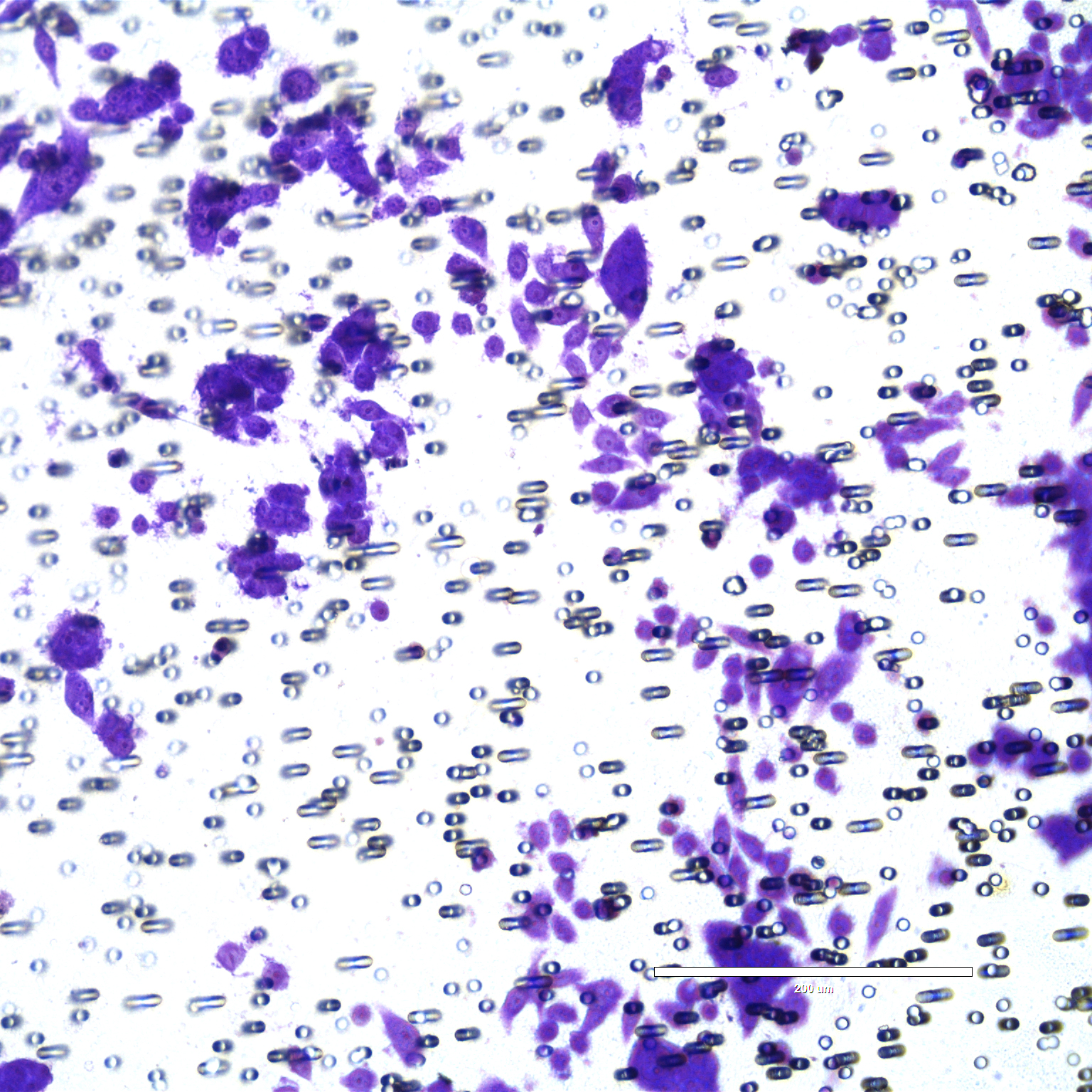

Supplement: Figure 3—figure supplement 1—source data 1. [file elife-68481-fig3-figsupp1-data1.zip › Figure 3-figure supplement 1 source data 1/S2C source data/migration-549:NC.jpg]

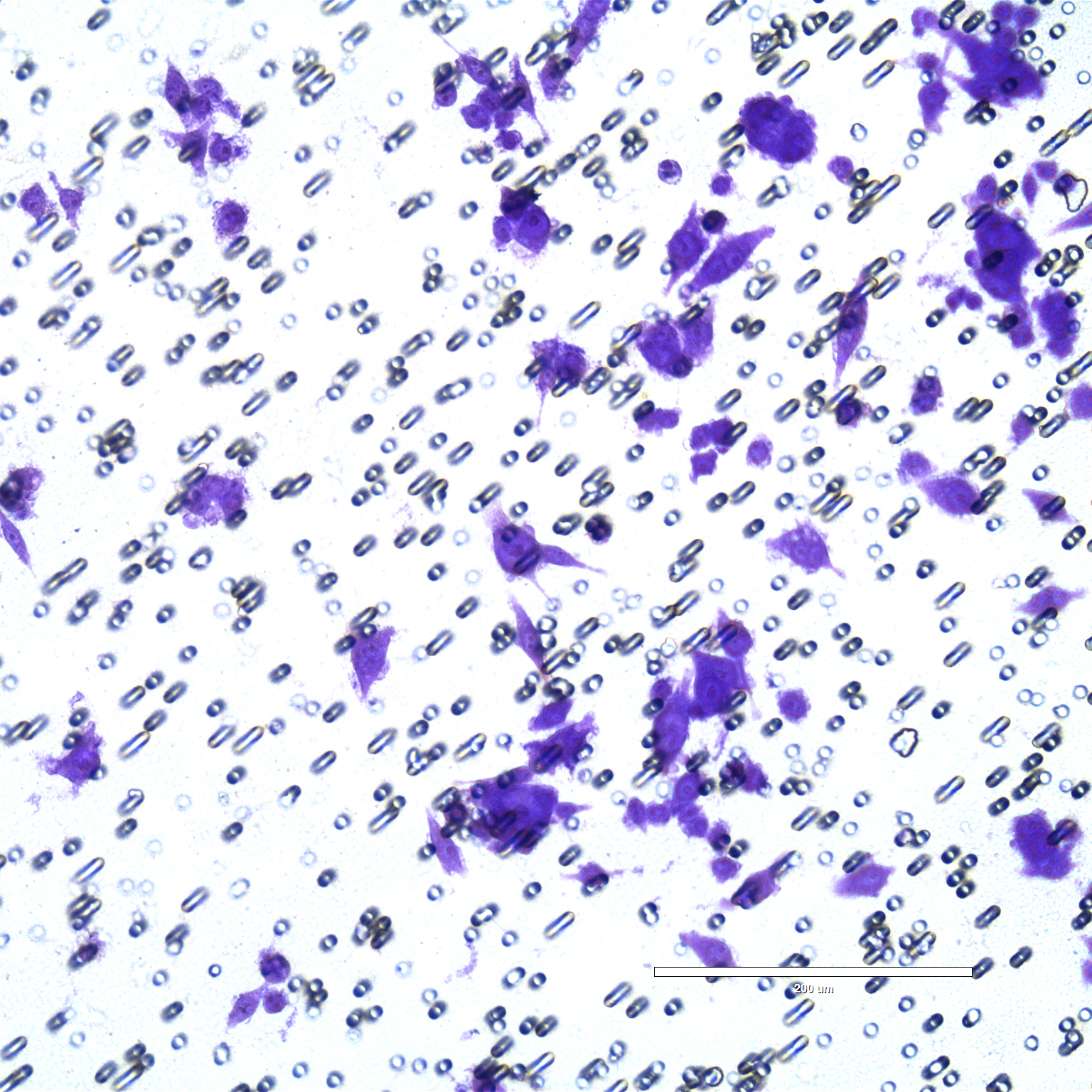

Supplement: Figure 3—figure supplement 1—source data 1. [file elife-68481-fig3-figsupp1-data1.zip › Figure 3-figure supplement 1 source data 1/S2C source data/migration-shTLN1.jpg]

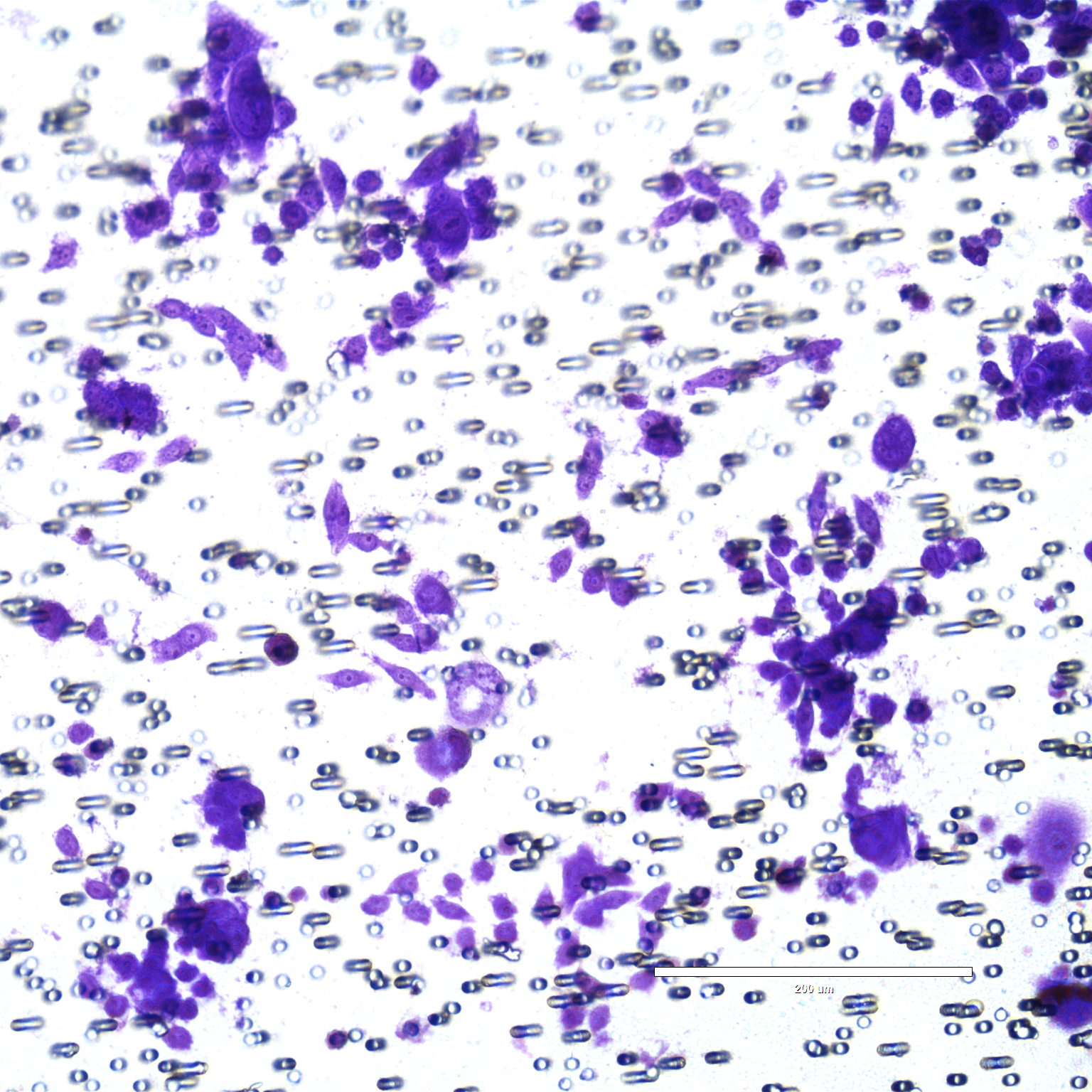

Supplement: Figure 3—figure supplement 1—source data 1. [file elife-68481-fig3-figsupp1-data1.zip › Figure 3-figure supplement 1 source data 1/S2C source data/invasion-549:NC.jpg]

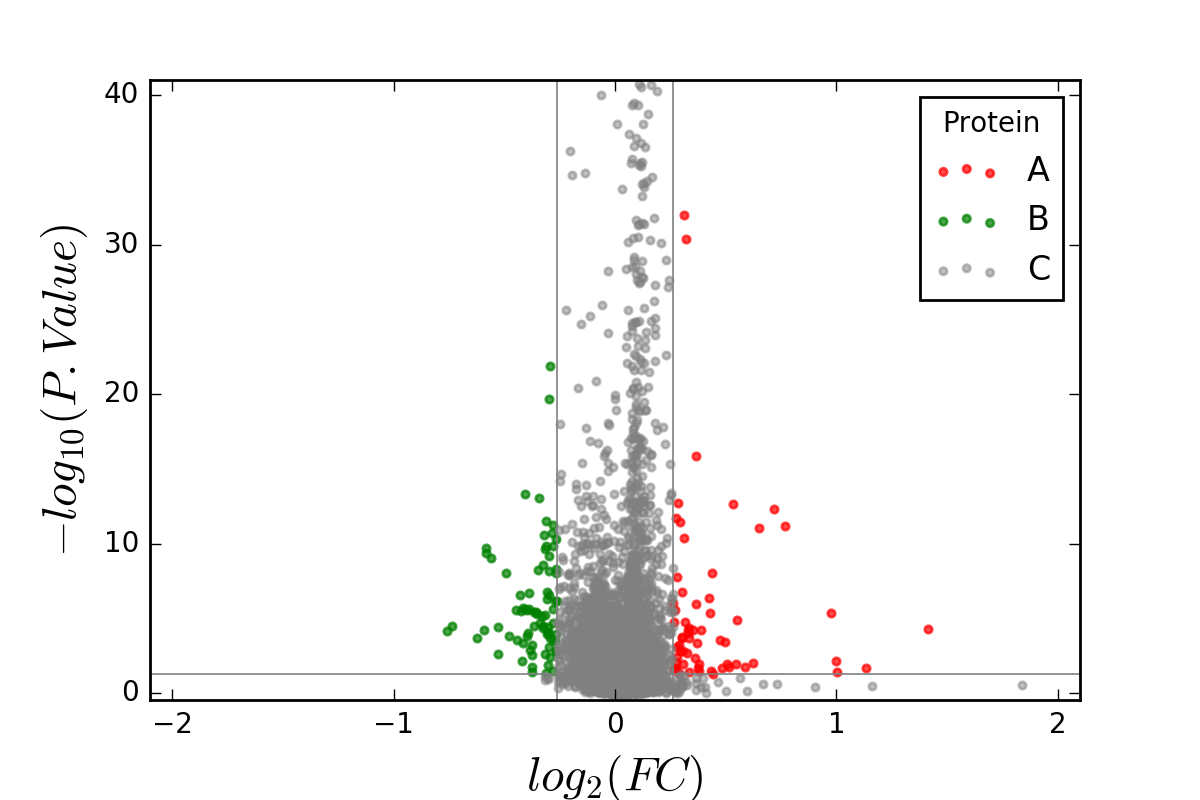

Supplement: Figure 3—figure supplement 2—source data 1. [file elife-68481-fig3-figsupp2-data1.zip › Figure 3-figure supplement 2 source data 1/S3A source data.png]

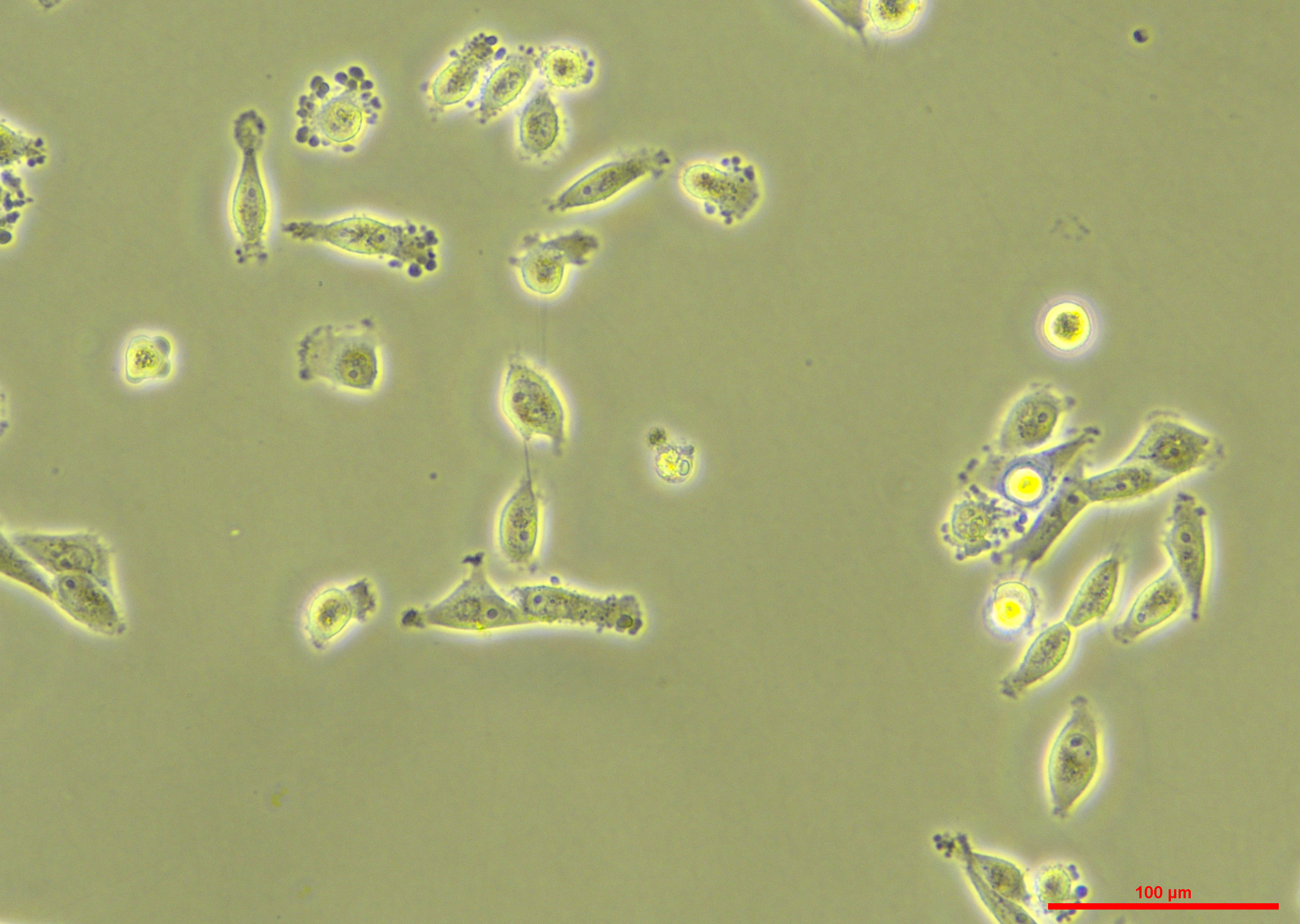

Supplement: Figure 4—source data 1. [file elife-68481-fig4-data1.zip › Figure 4-source data 1/4D source data/shTLN1-20x.tif]

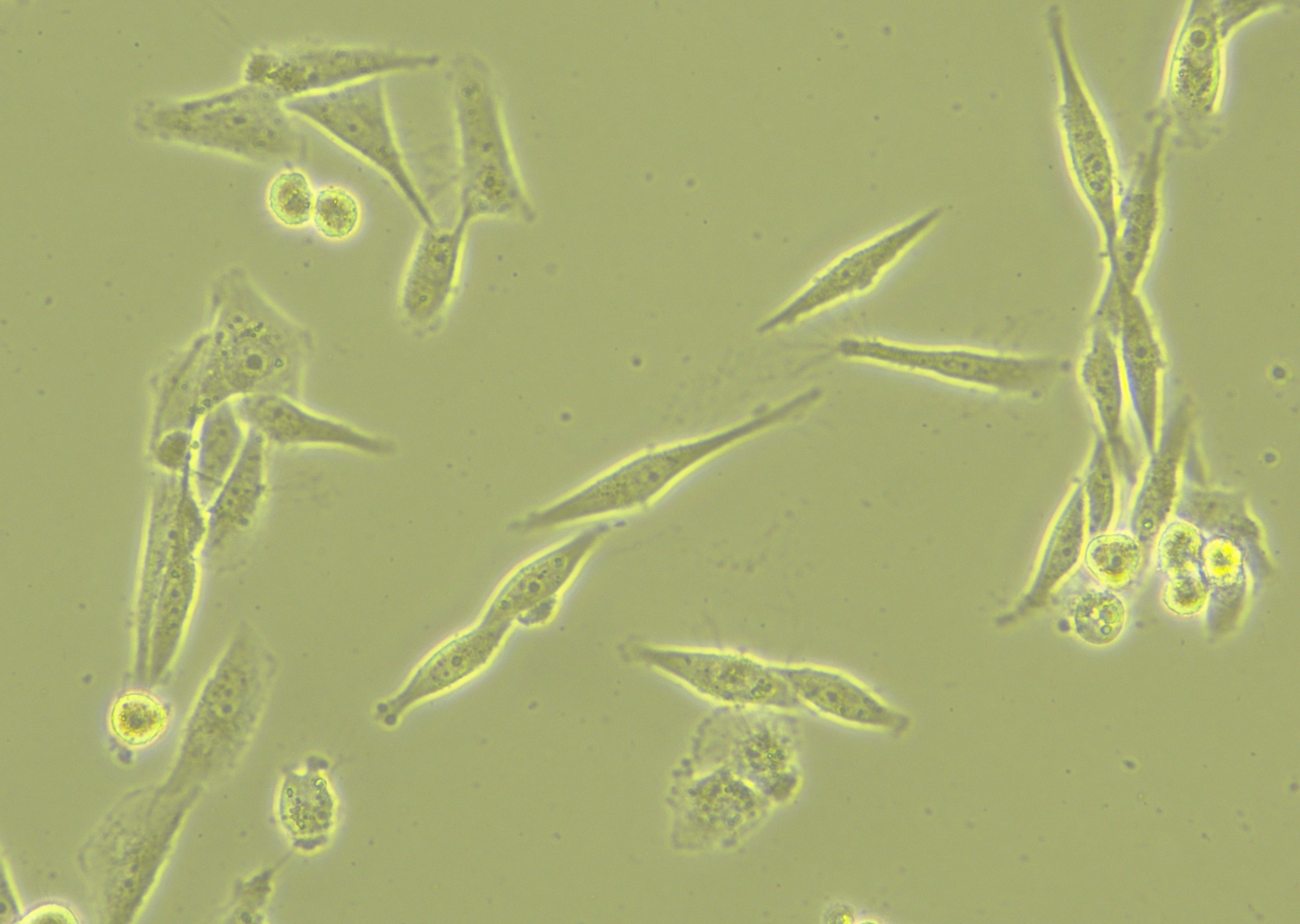

Supplement: Figure 4—source data 1. [file elife-68481-fig4-data1.zip › Figure 4-source data 1/4D source data/NC-20x.tif]

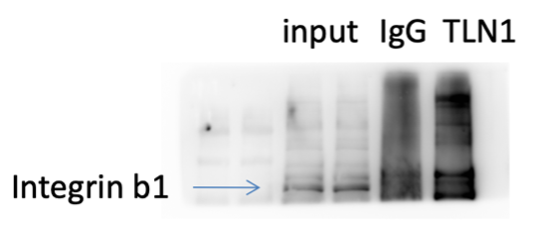

Supplement: Figure 4—source data 1. [file elife-68481-fig4-data1.zip › Figure 4-source data 1/4A source data/IP-1.png]

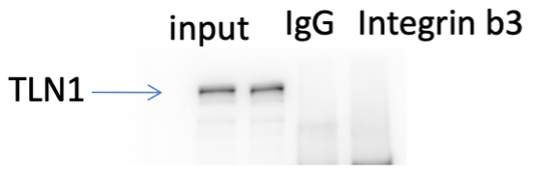

Supplement: Figure 4—source data 1. [file elife-68481-fig4-data1.zip › Figure 4-source data 1/4A source data/IP-3.png]

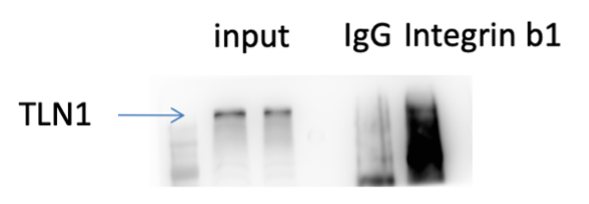

Supplement: Figure 4—source data 1. [file elife-68481-fig4-data1.zip › Figure 4-source data 1/4A source data/IP-2.png]

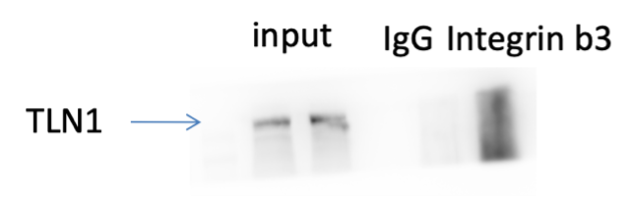

Supplement: Figure 4—source data 1. [file elife-68481-fig4-data1.zip › Figure 4-source data 1/4A source data/IP-4.png]

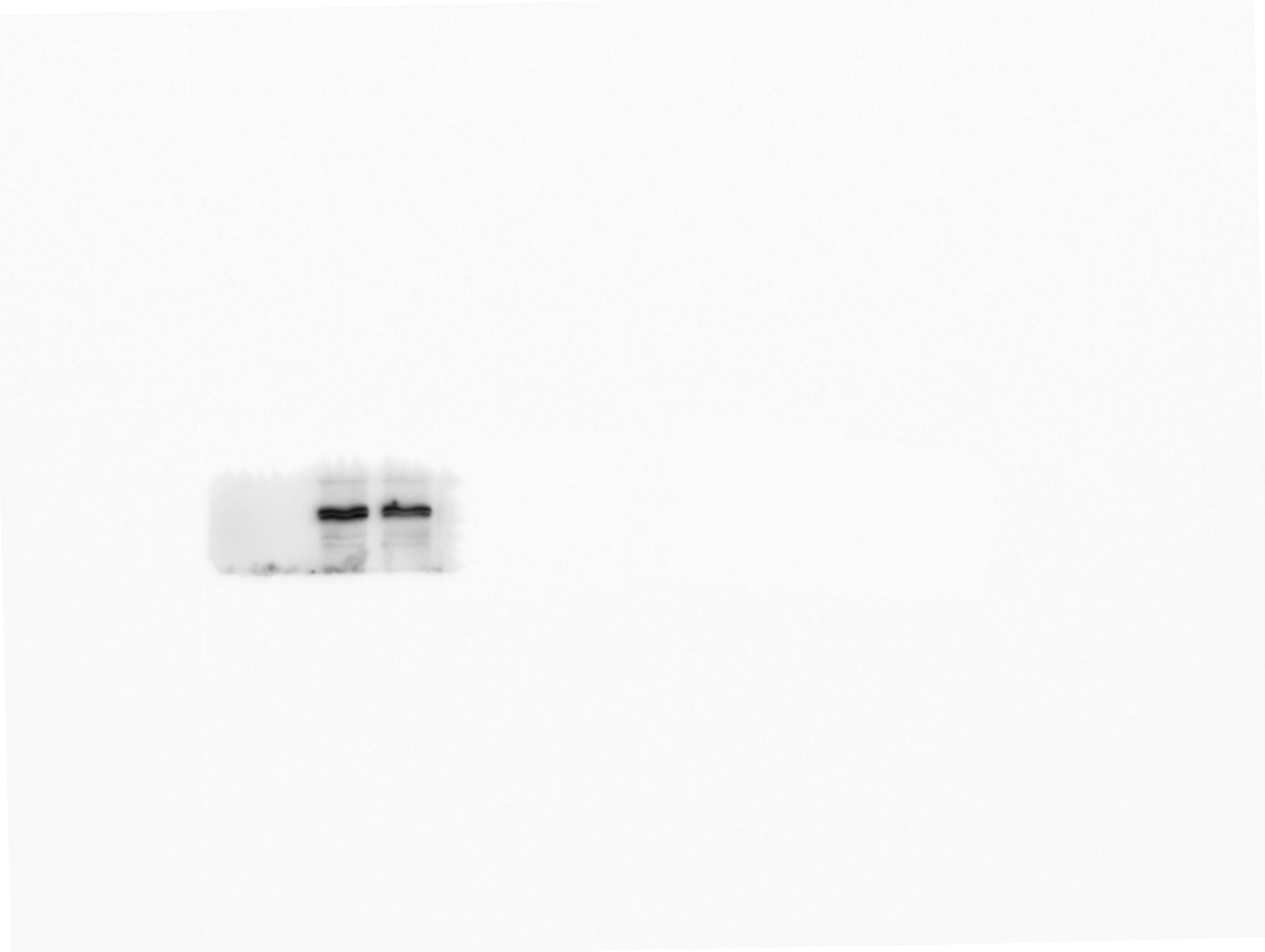

Supplement: Figure 4—source data 1. [file elife-68481-fig4-data1.zip › Figure 4-source data 1/4E source data/FAK.tif]

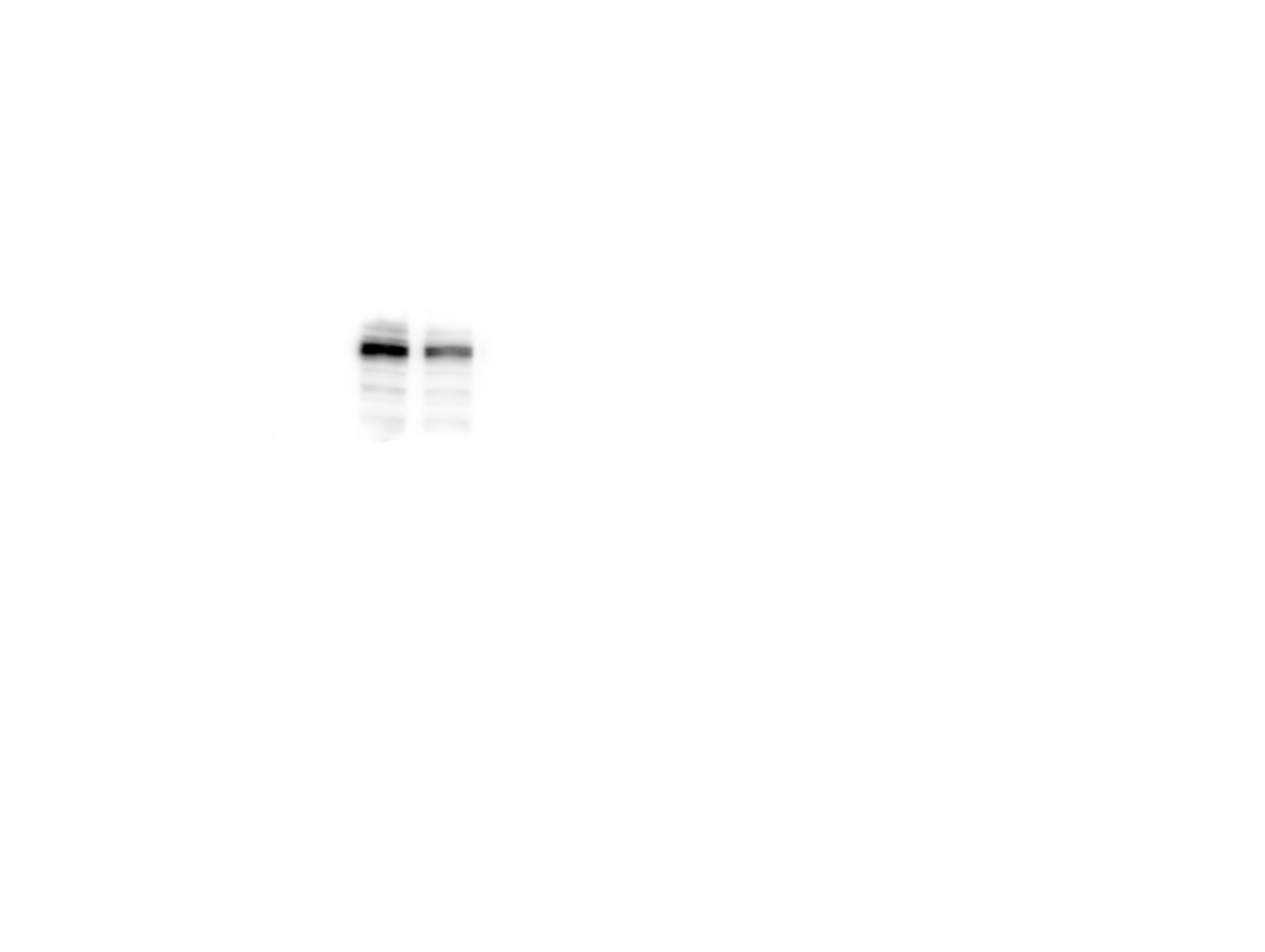

Supplement: Figure 4—source data 1. [file elife-68481-fig4-data1.zip › Figure 4-source data 1/4E source data/pFAK.tif]

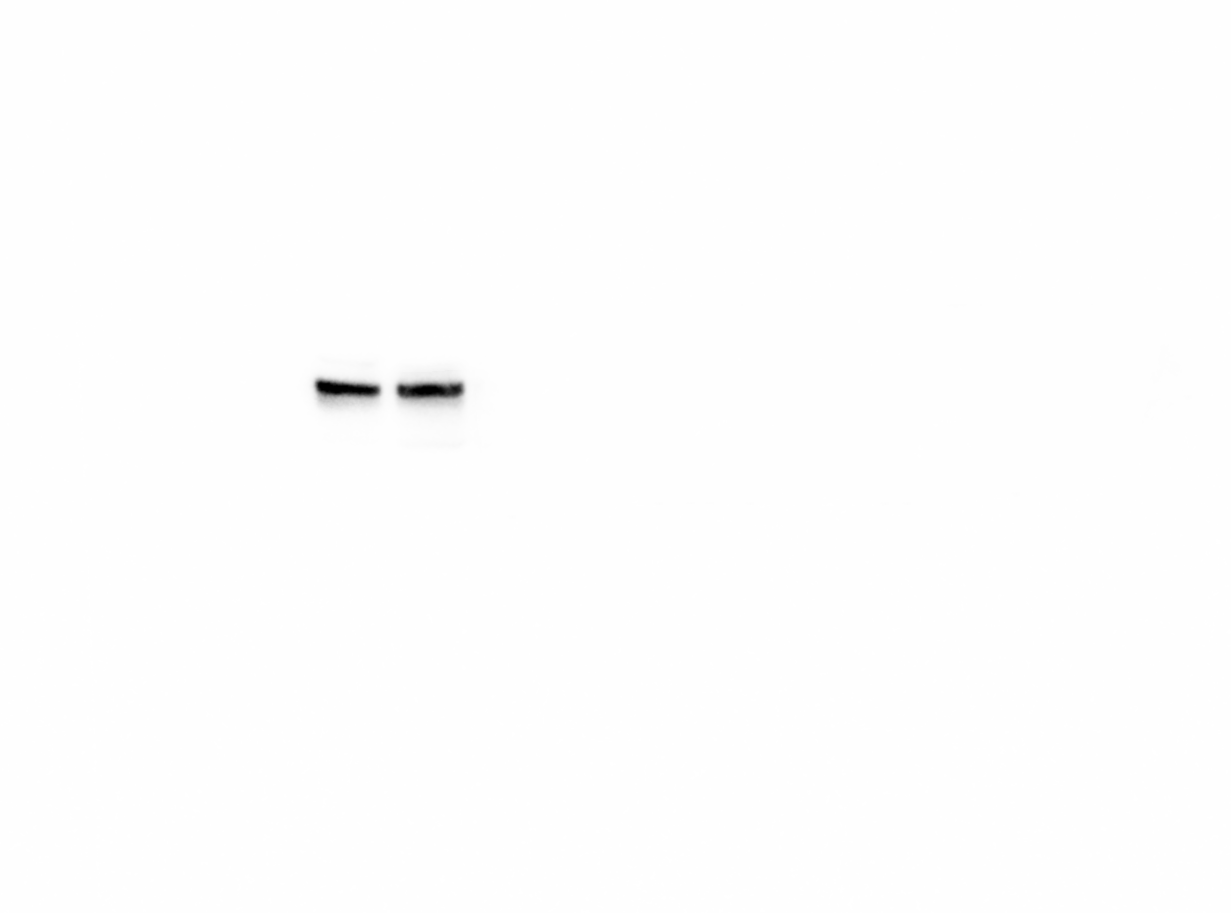

Supplement: Figure 4—source data 1. [file elife-68481-fig4-data1.zip › Figure 4-source data 1/4E source data/b3.tif]

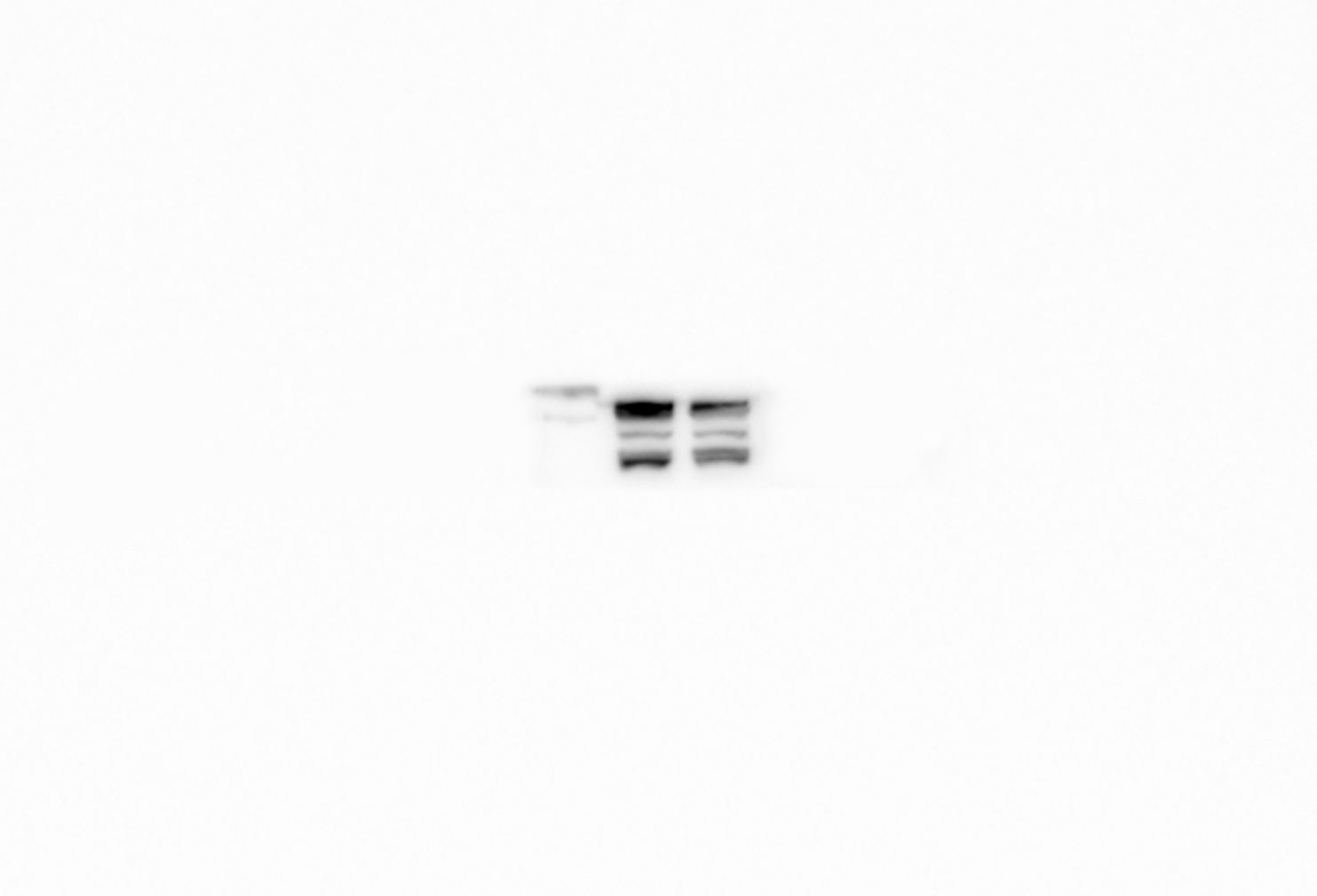

Supplement: Figure 4—source data 1. [file elife-68481-fig4-data1.zip › Figure 4-source data 1/4E source data/pAKT.tif]

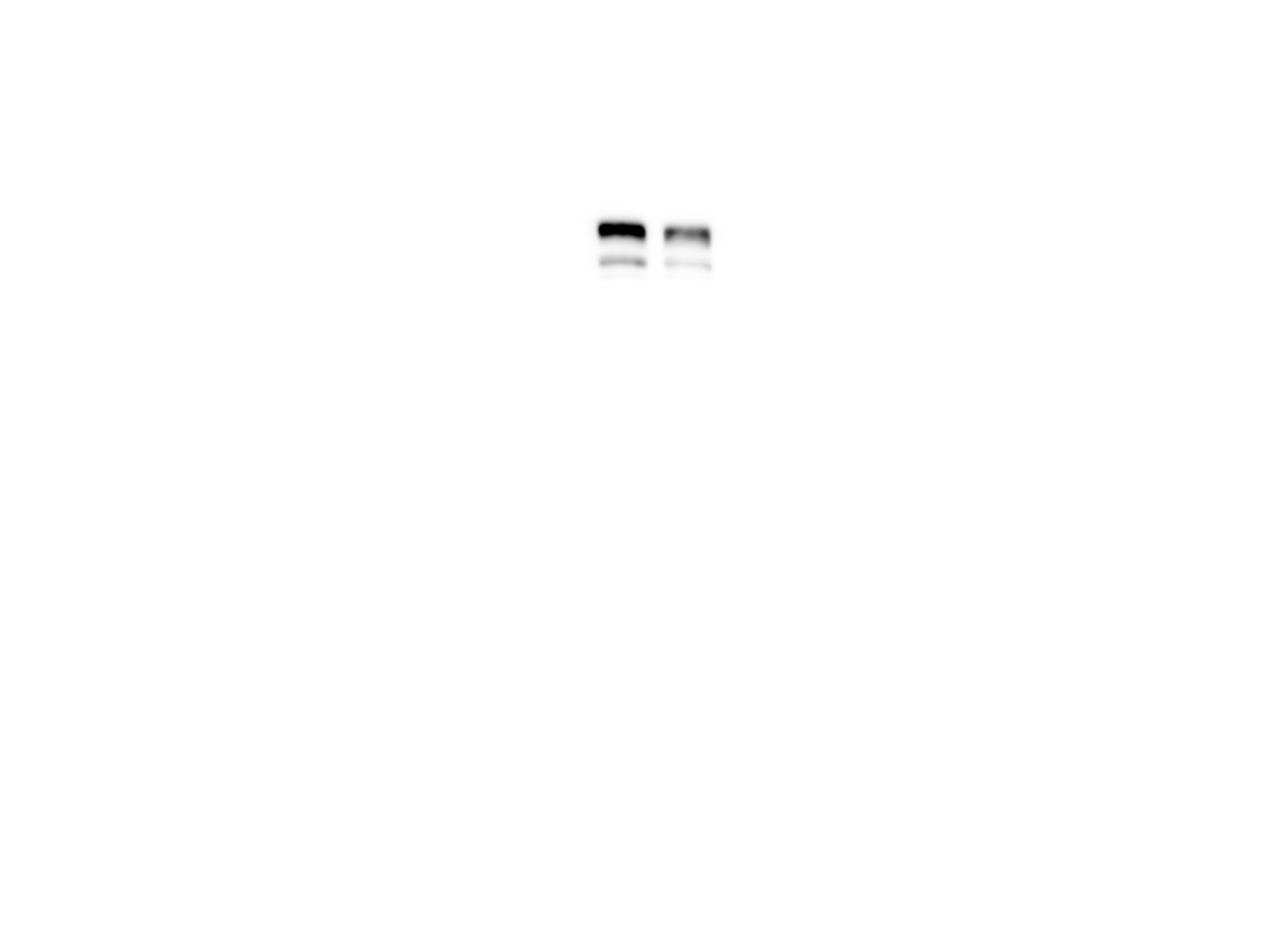

Supplement: Figure 4—source data 1. [file elife-68481-fig4-data1.zip › Figure 4-source data 1/4E source data/b1.tif]

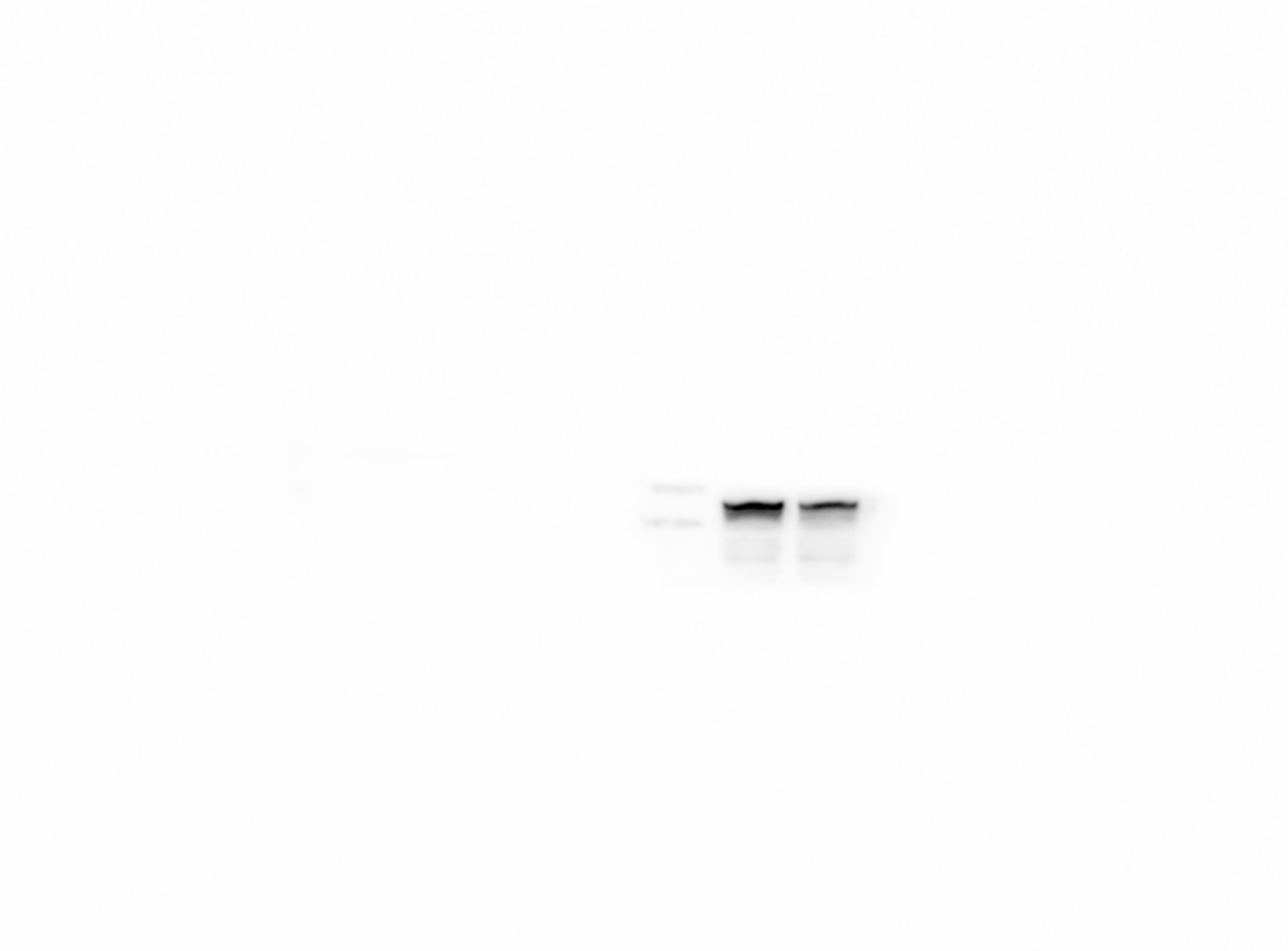

Supplement: Figure 4—source data 1. [file elife-68481-fig4-data1.zip › Figure 4-source data 1/4E source data/AKT.tif]

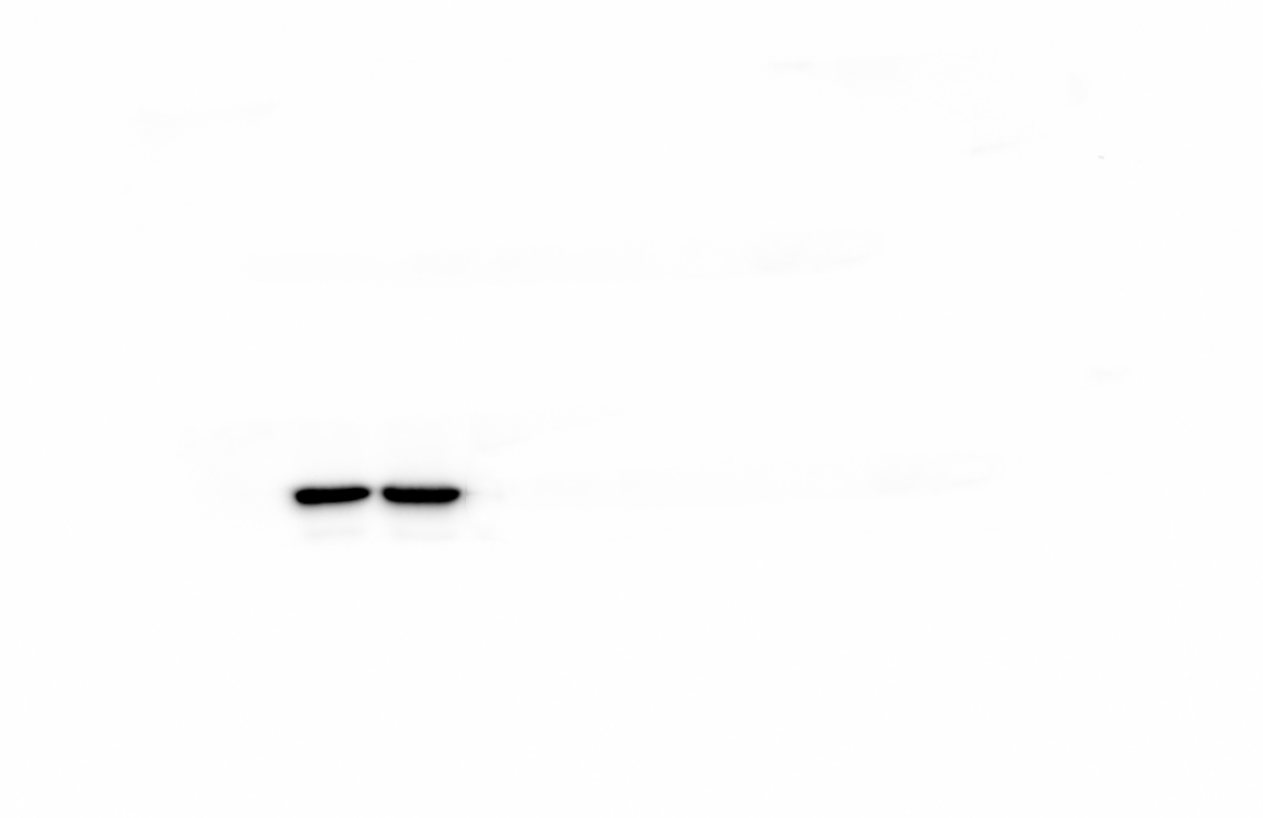

Supplement: Figure 4—source data 1. [file elife-68481-fig4-data1.zip › Figure 4-source data 1/4E source data/gapdh.tif]

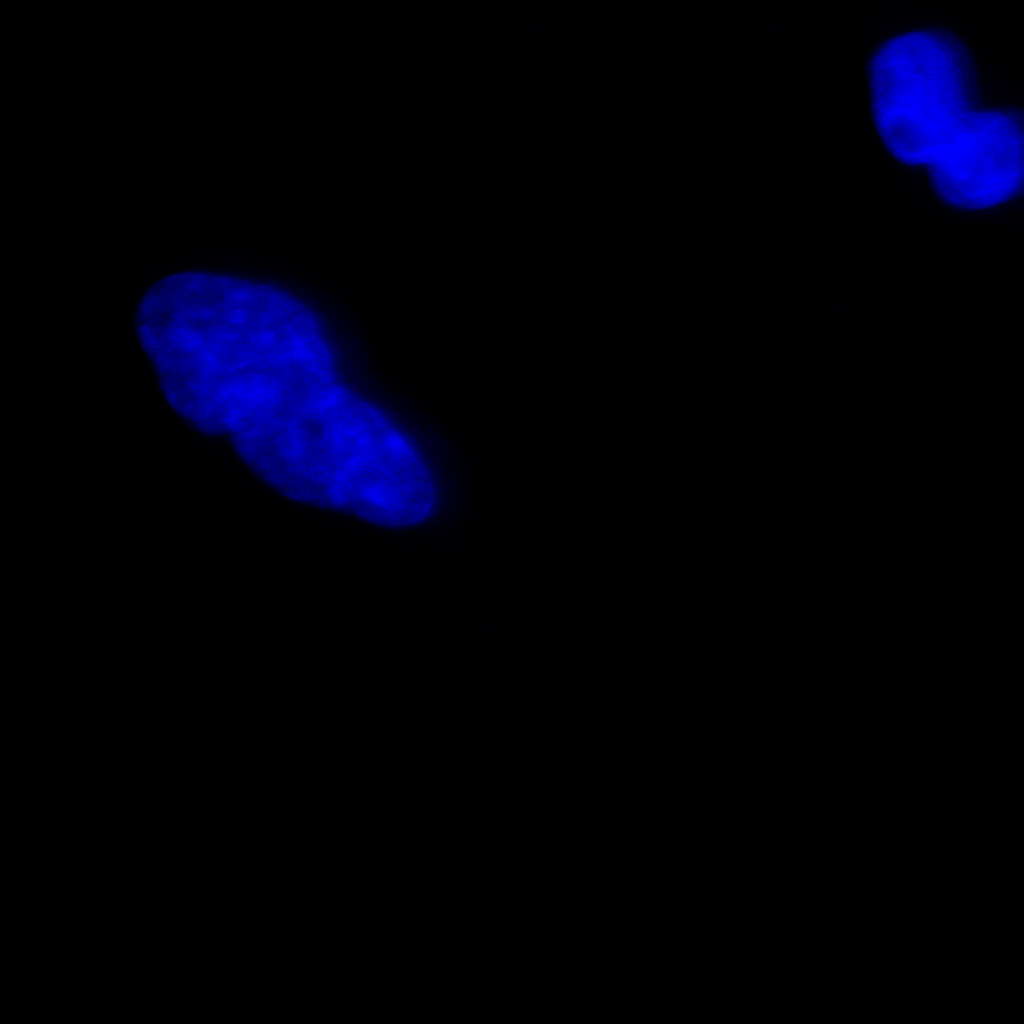

Supplement: Figure 4—source data 1. [file elife-68481-fig4-data1.zip › Figure 4-source data 1/4BC source data/4B-IF/DAPI.tif]

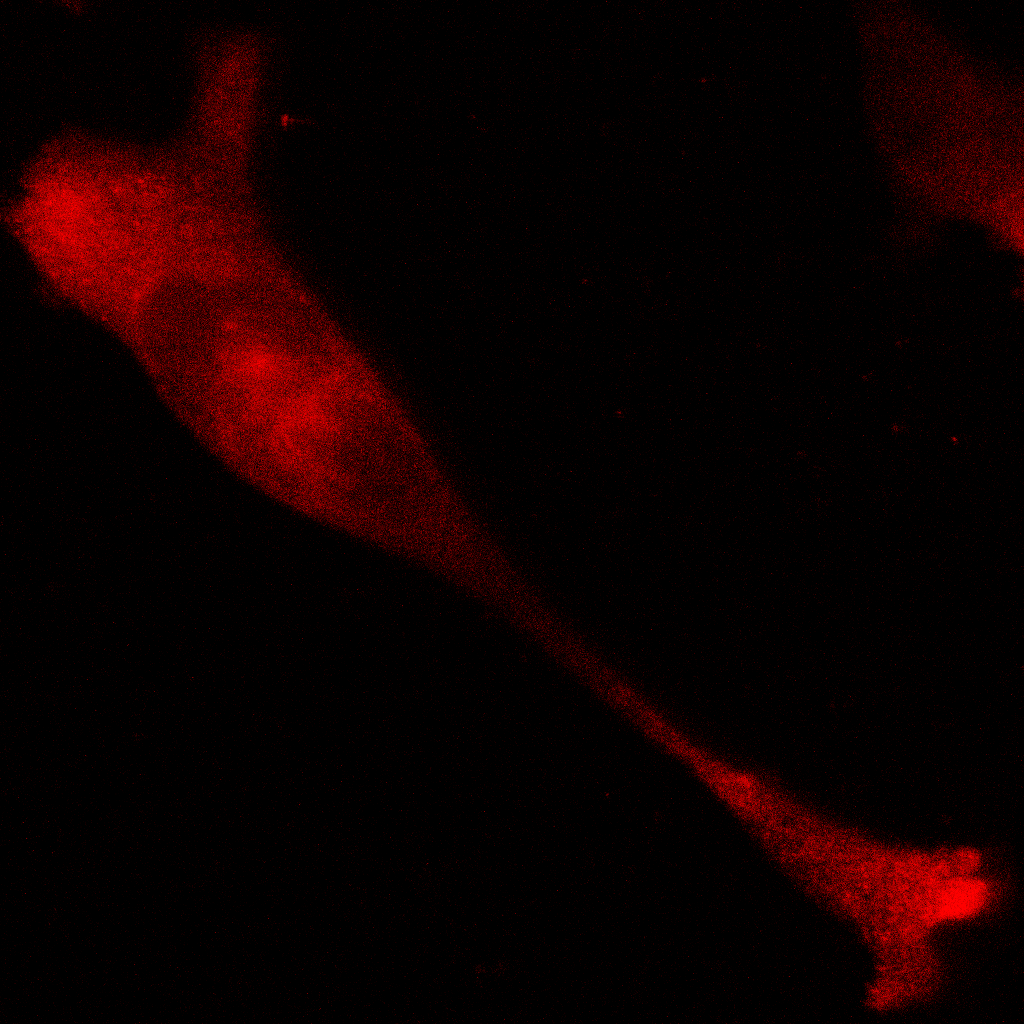

Supplement: Figure 4—source data 1. [file elife-68481-fig4-data1.zip › Figure 4-source data 1/4BC source data/4B-IF/TLN1.tif]

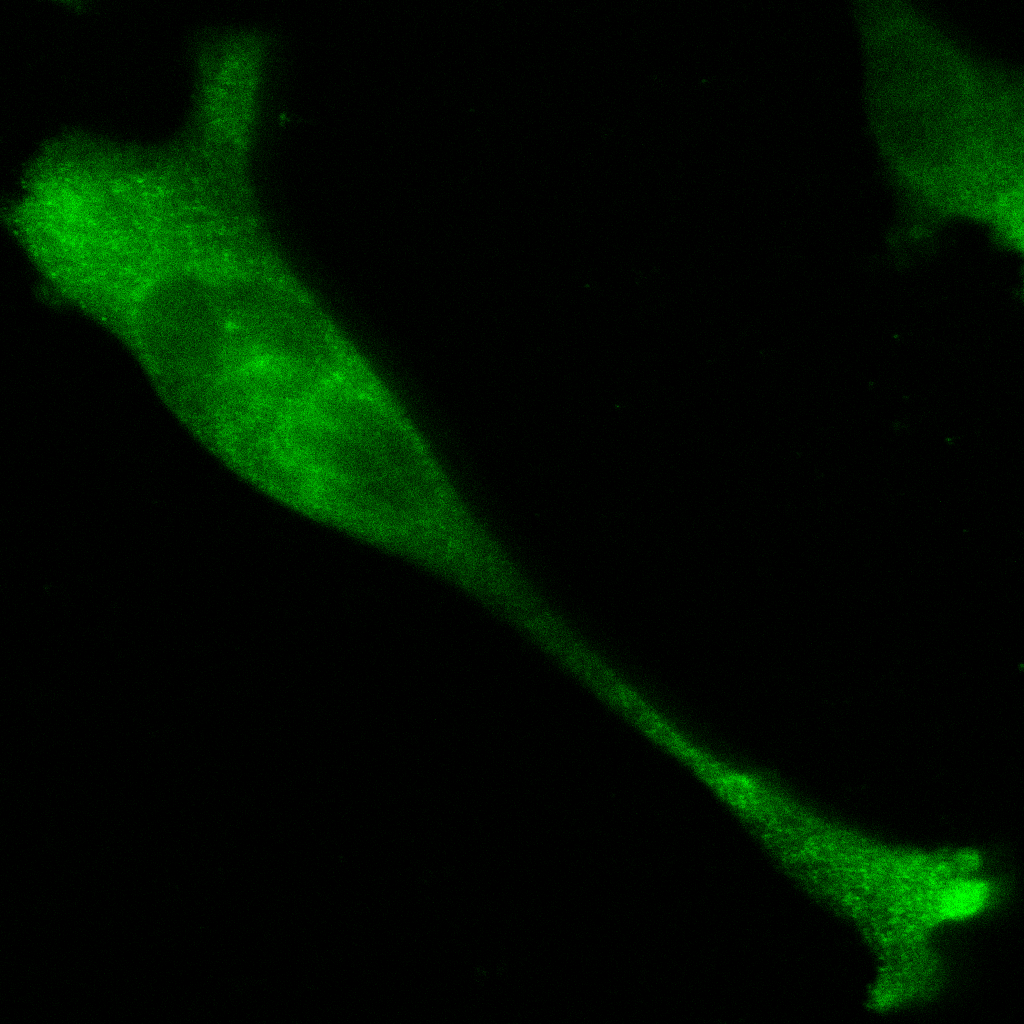

Supplement: Figure 4—source data 1. [file elife-68481-fig4-data1.zip › Figure 4-source data 1/4BC source data/4B-IF/ITGB.tif]

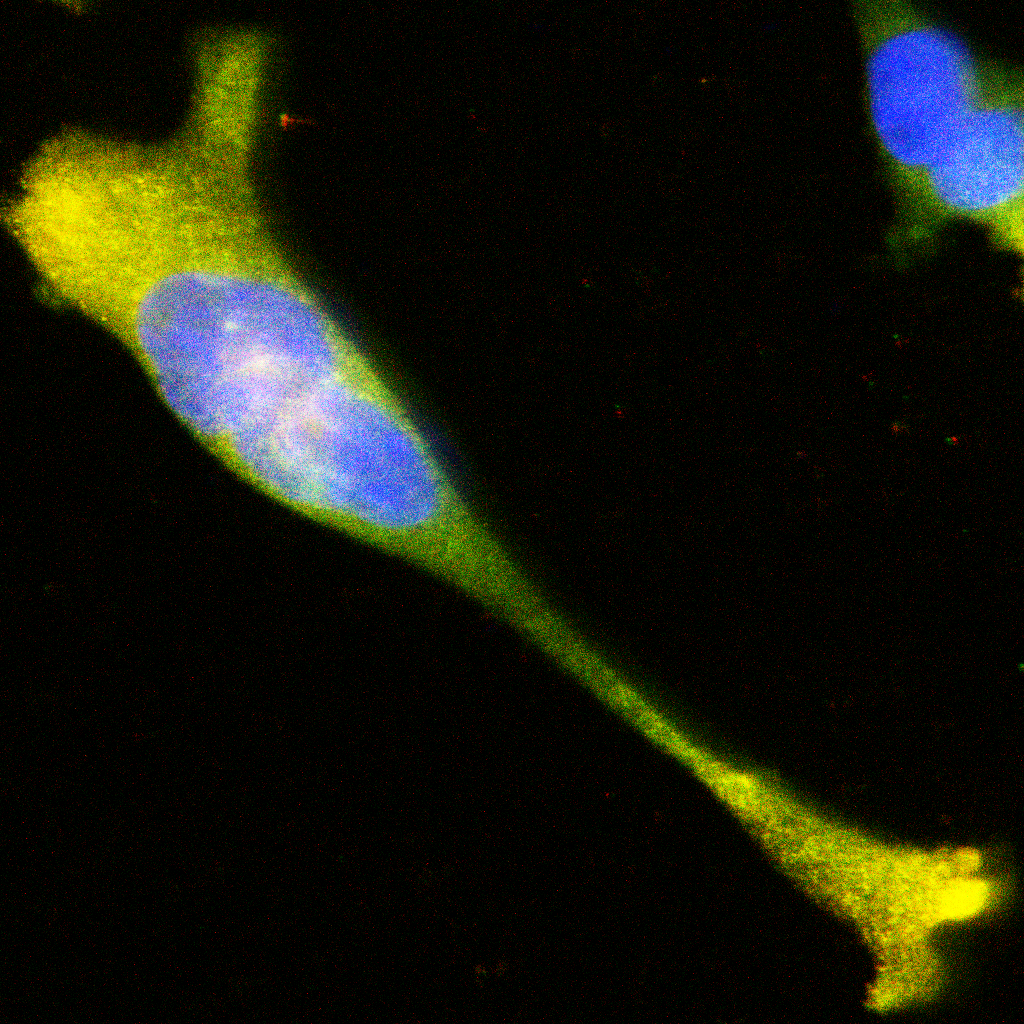

Supplement: Figure 4—source data 1. [file elife-68481-fig4-data1.zip › Figure 4-source data 1/4BC source data/4B-IF/Merge.tif]

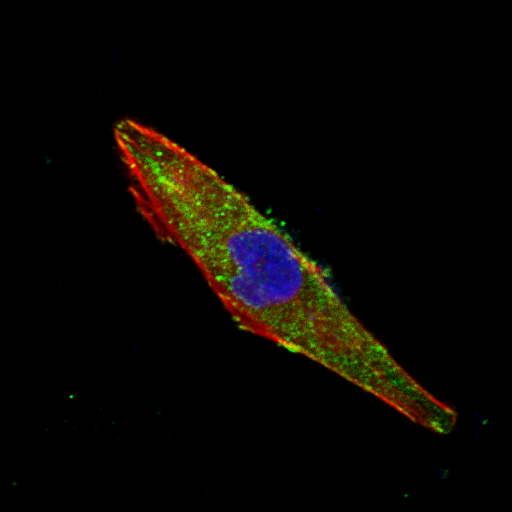

Supplement: Figure 4—source data 1. [file elife-68481-fig4-data1.zip › Figure 4-source data 1/4BC source data/4C-IF/nc-800/nc-800-7_merge.tif]

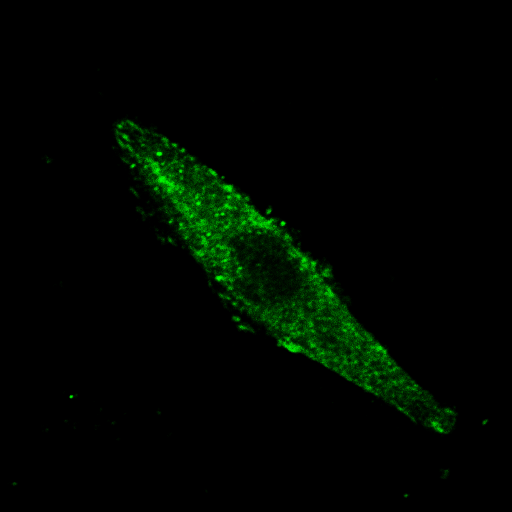

Supplement: Figure 4—source data 1. [file elife-68481-fig4-data1.zip › Figure 4-source data 1/4BC source data/4C-IF/nc-800/nc-800-7_c2.tif]

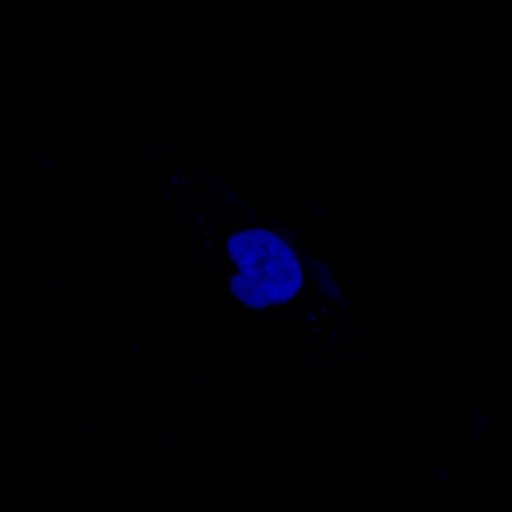

Supplement: Figure 4—source data 1. [file elife-68481-fig4-data1.zip › Figure 4-source data 1/4BC source data/4C-IF/nc-800/nc-800-7_c3.tif]

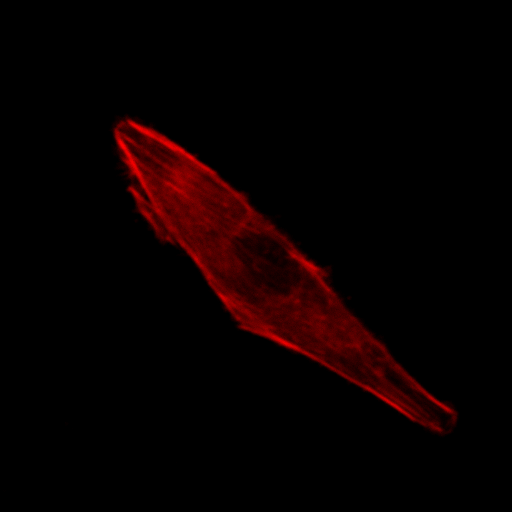

Supplement: Figure 4—source data 1. [file elife-68481-fig4-data1.zip › Figure 4-source data 1/4BC source data/4C-IF/nc-800/nc-800-7_c1.tif]

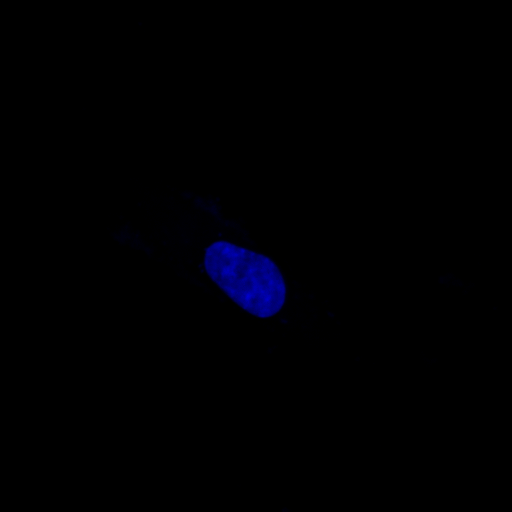

Supplement: Figure 4—source data 1. [file elife-68481-fig4-data1.zip › Figure 4-source data 1/4BC source data/4C-IF/shT-800/shT-800-4_c3.tif]

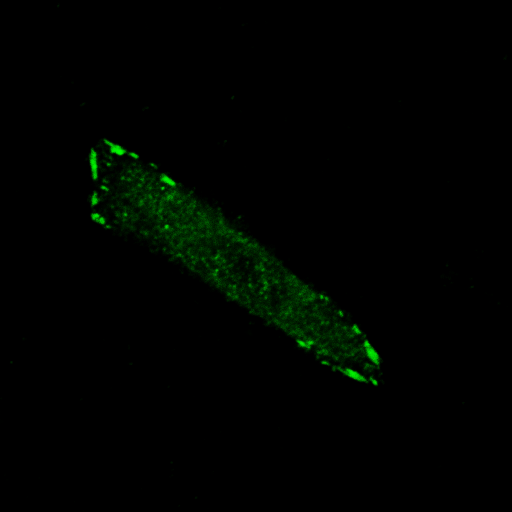

Supplement: Figure 4—source data 1. [file elife-68481-fig4-data1.zip › Figure 4-source data 1/4BC source data/4C-IF/shT-800/shT-800-4_c2.tif]

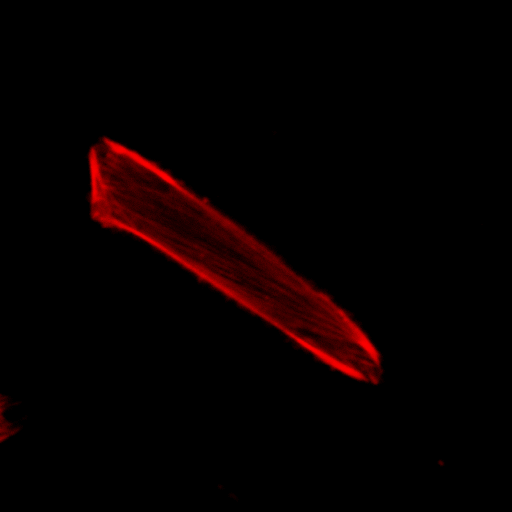

Supplement: Figure 4—source data 1. [file elife-68481-fig4-data1.zip › Figure 4-source data 1/4BC source data/4C-IF/shT-800/shT-800-4_c1.tif]

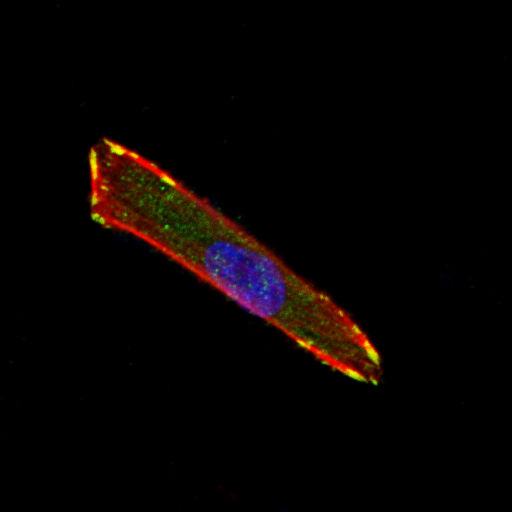

Supplement: Figure 4—source data 1. [file elife-68481-fig4-data1.zip › Figure 4-source data 1/4BC source data/4C-IF/shT-800/shT-800-4_merge.tif]

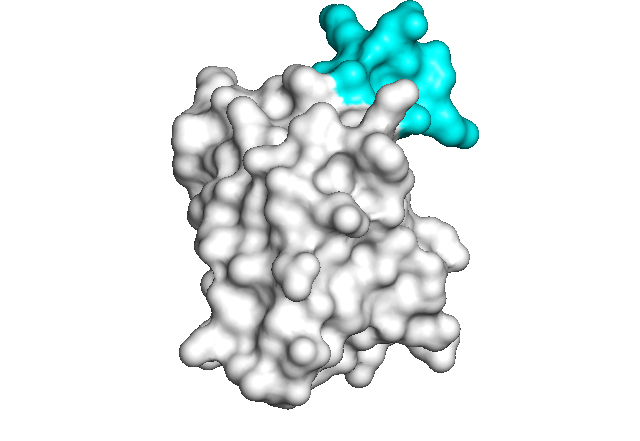

Supplement: Figure 5—source data 1. [file elife-68481-fig5-data1.zip › Figure 5-source data 1/5ABC source data/l2.png]

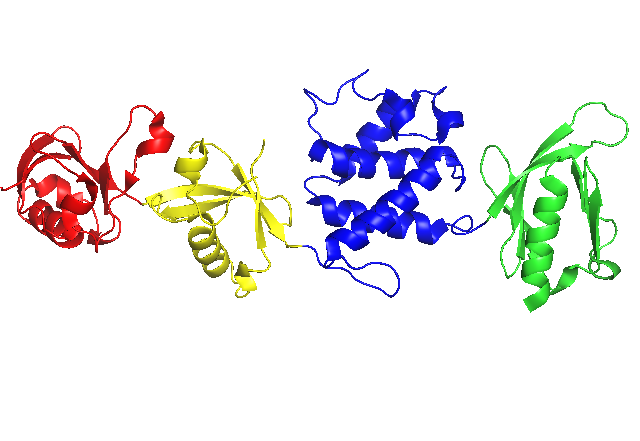

Supplement: Figure 5—source data 1. [file elife-68481-fig5-data1.zip › Figure 5-source data 1/5ABC source data/talin1-head.png]

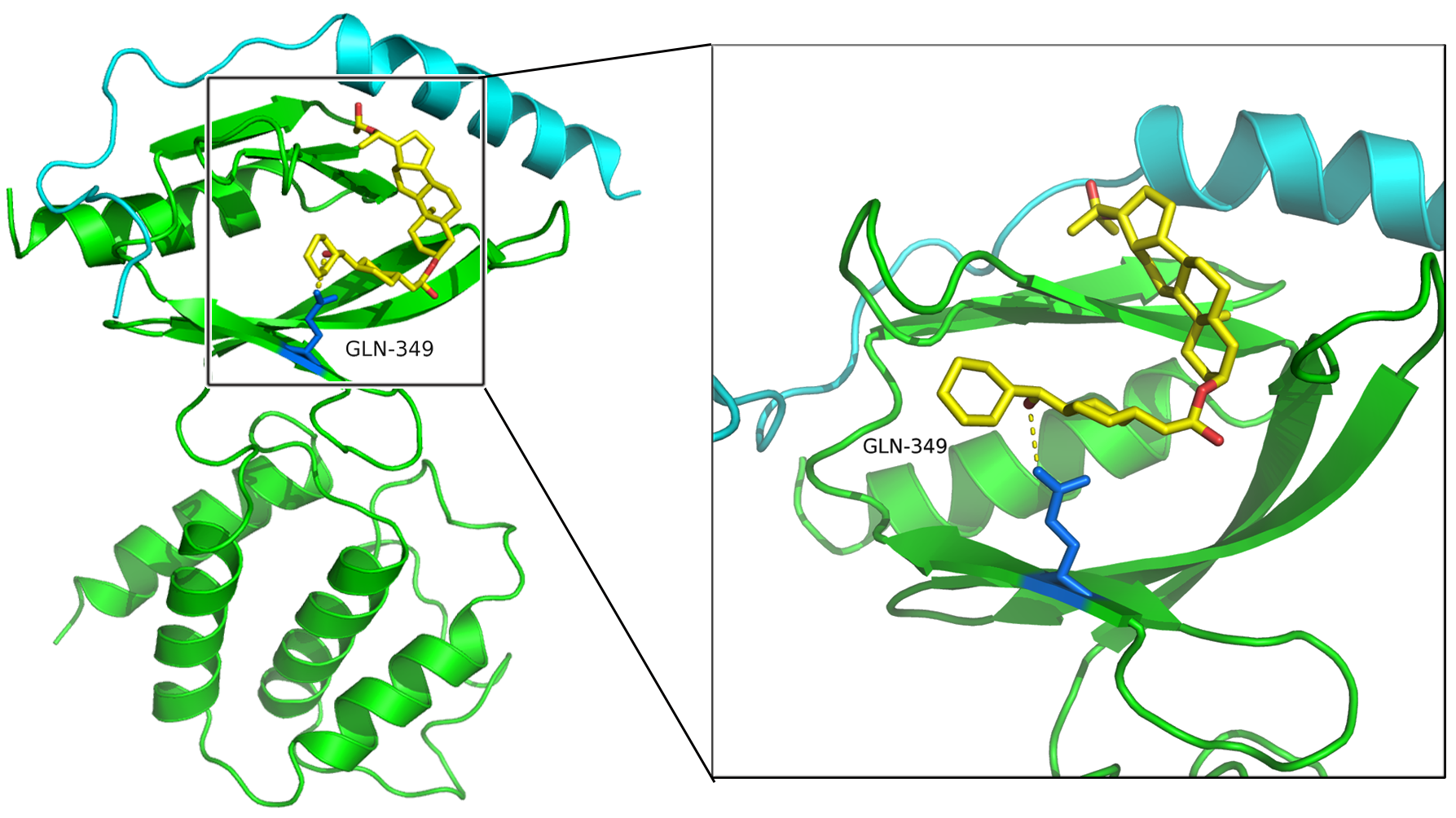

Supplement: Figure 5—source data 1. [file elife-68481-fig5-data1.zip › Figure 5-source data 1/5ABC source data/cartoon.tif]

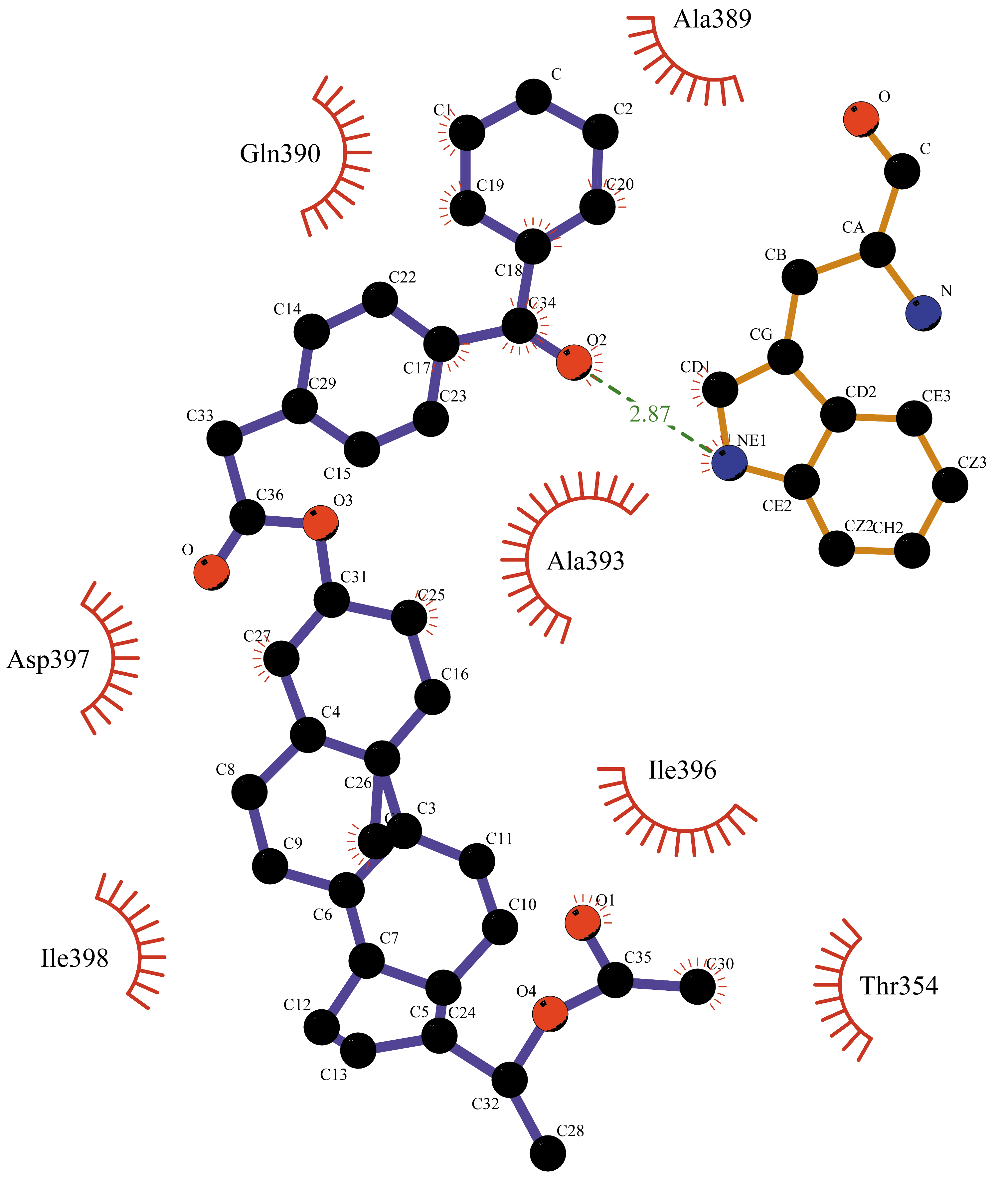

Supplement: Figure 5—source data 1. [file elife-68481-fig5-data1.zip › Figure 5-source data 1/5ABC source data/C67399.png]

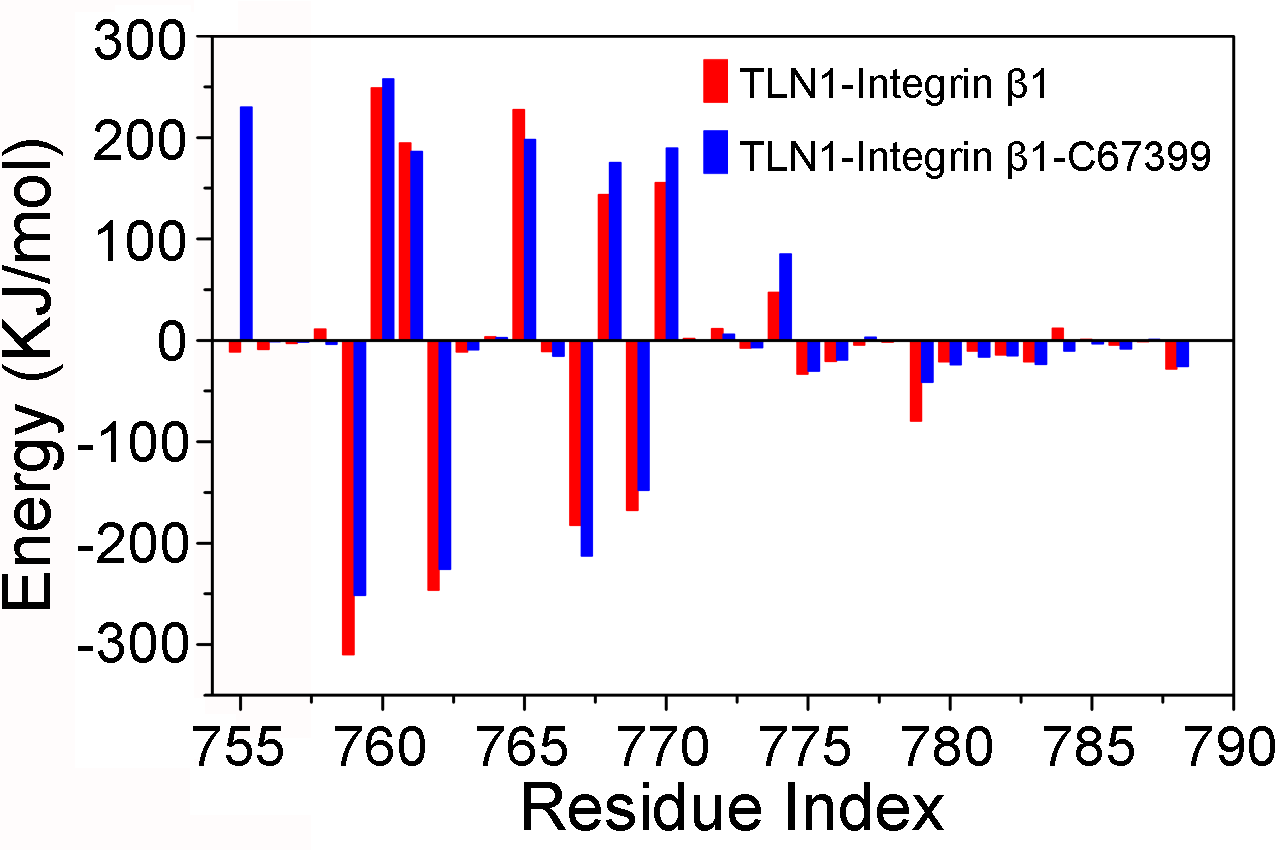

Supplement: Figure 5—source data 1. [file elife-68481-fig5-data1.zip › Figure 5-source data 1/5D source data/beta1-energyσêåΦoú1.tif]

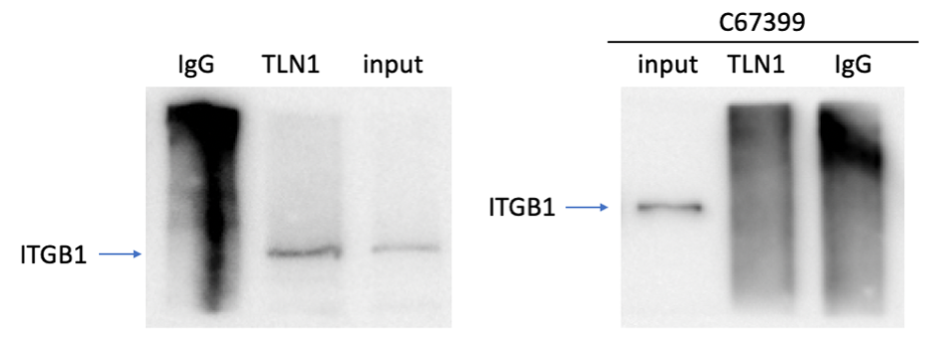

Supplement: Figure 5—source data 1. [file elife-68481-fig5-data1.zip › Figure 5-source data 1/5J source data/IP-6.png]

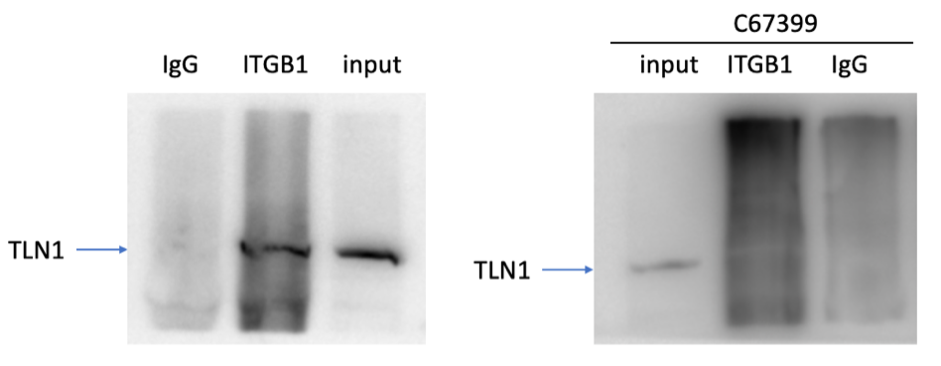

Supplement: Figure 5—source data 1. [file elife-68481-fig5-data1.zip › Figure 5-source data 1/5J source data/IP-5.png]

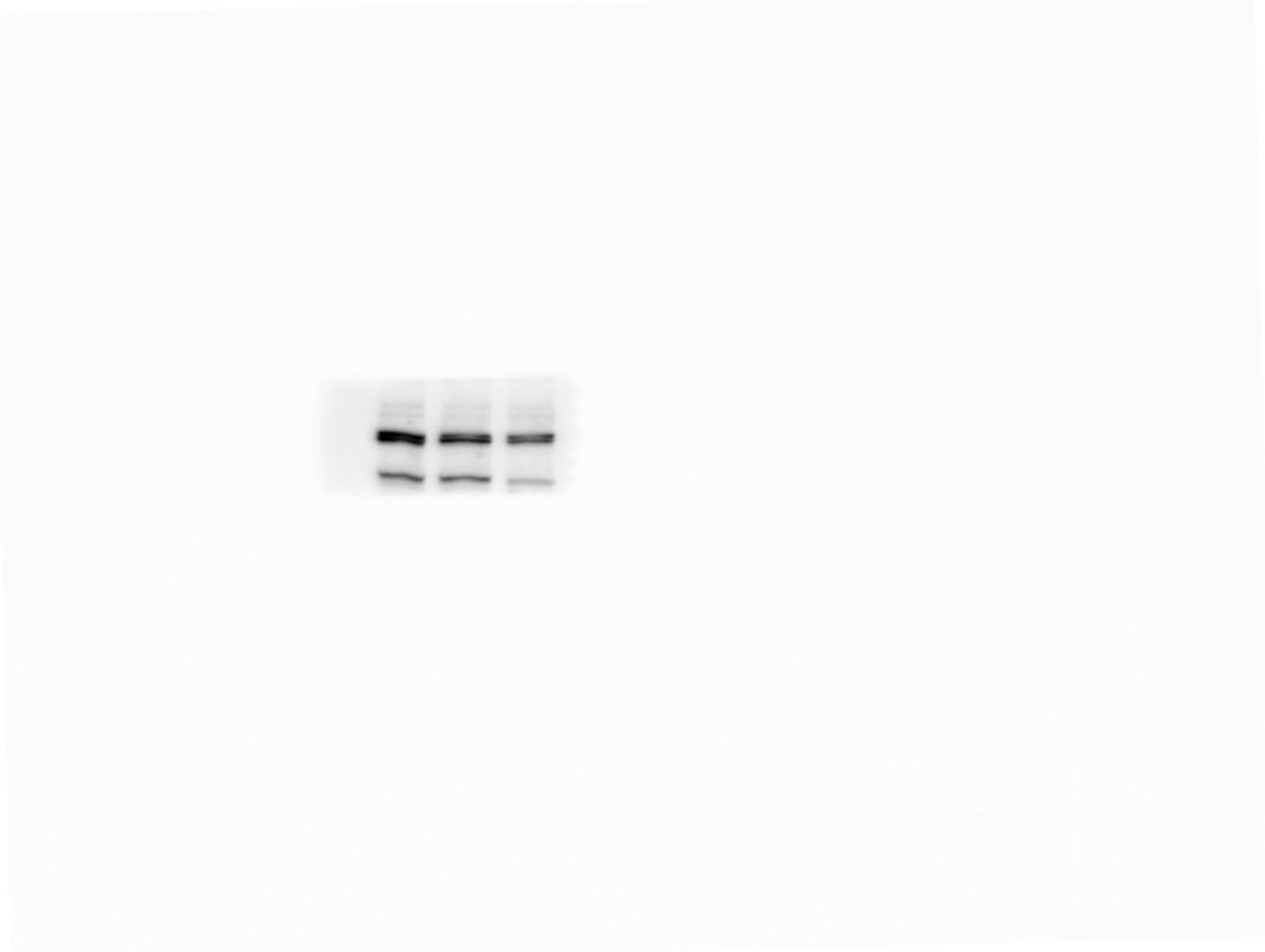

Supplement: Figure 5—source data 1. [file elife-68481-fig5-data1.zip › Figure 5-source data 1/5I source data/FAK.tif]

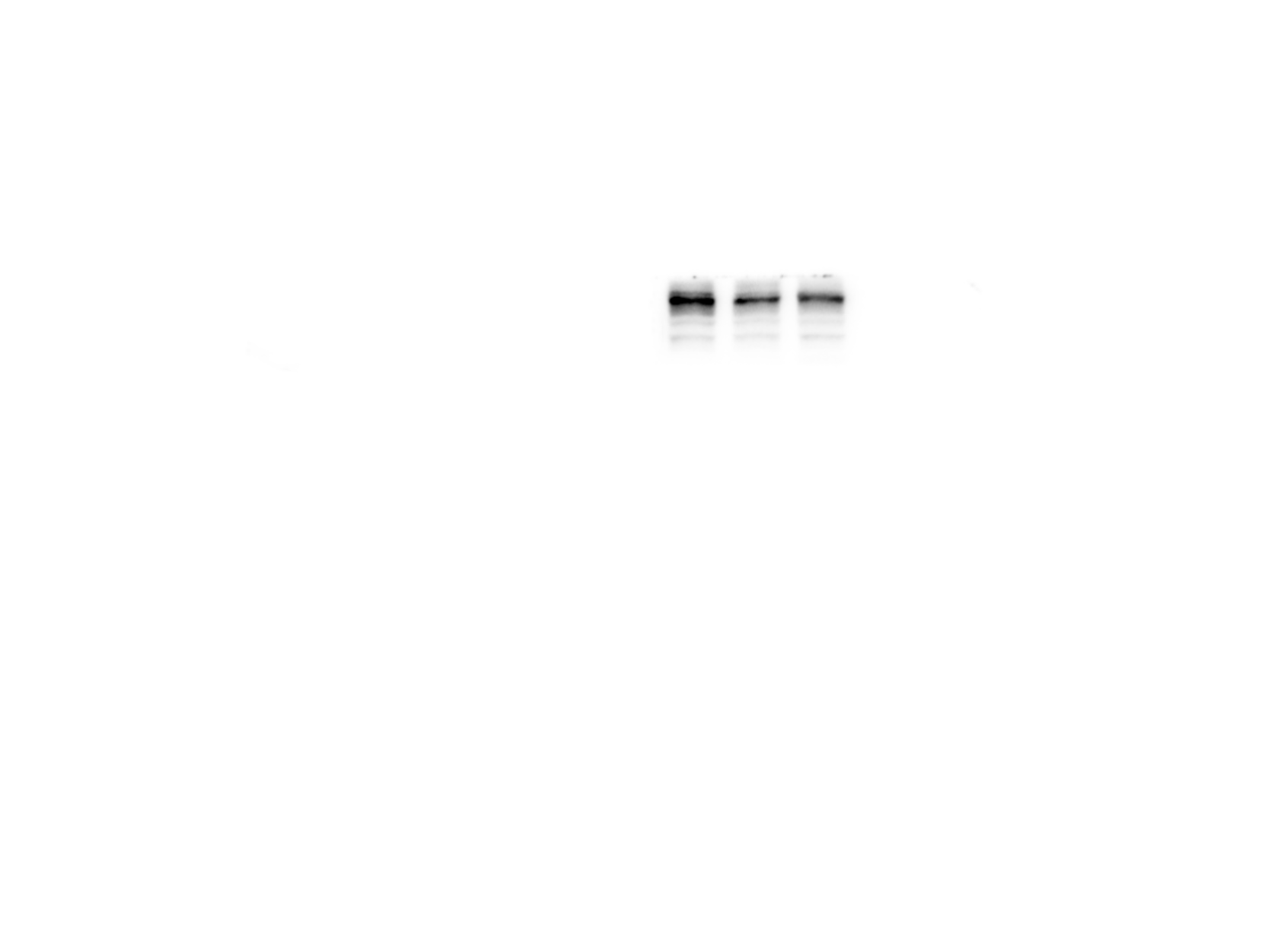

Supplement: Figure 5—source data 1. [file elife-68481-fig5-data1.zip › Figure 5-source data 1/5I source data/pFAK.tif]

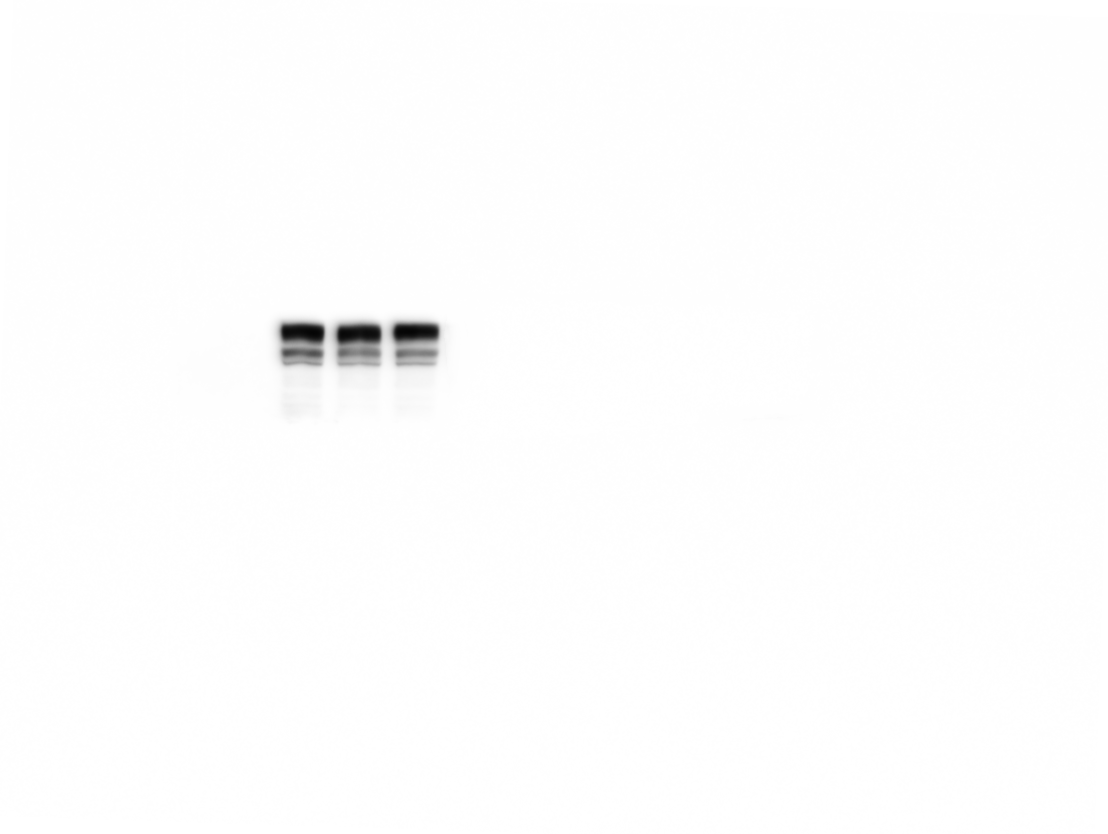

Supplement: Figure 5—source data 1. [file elife-68481-fig5-data1.zip › Figure 5-source data 1/5I source data/b3.tif]

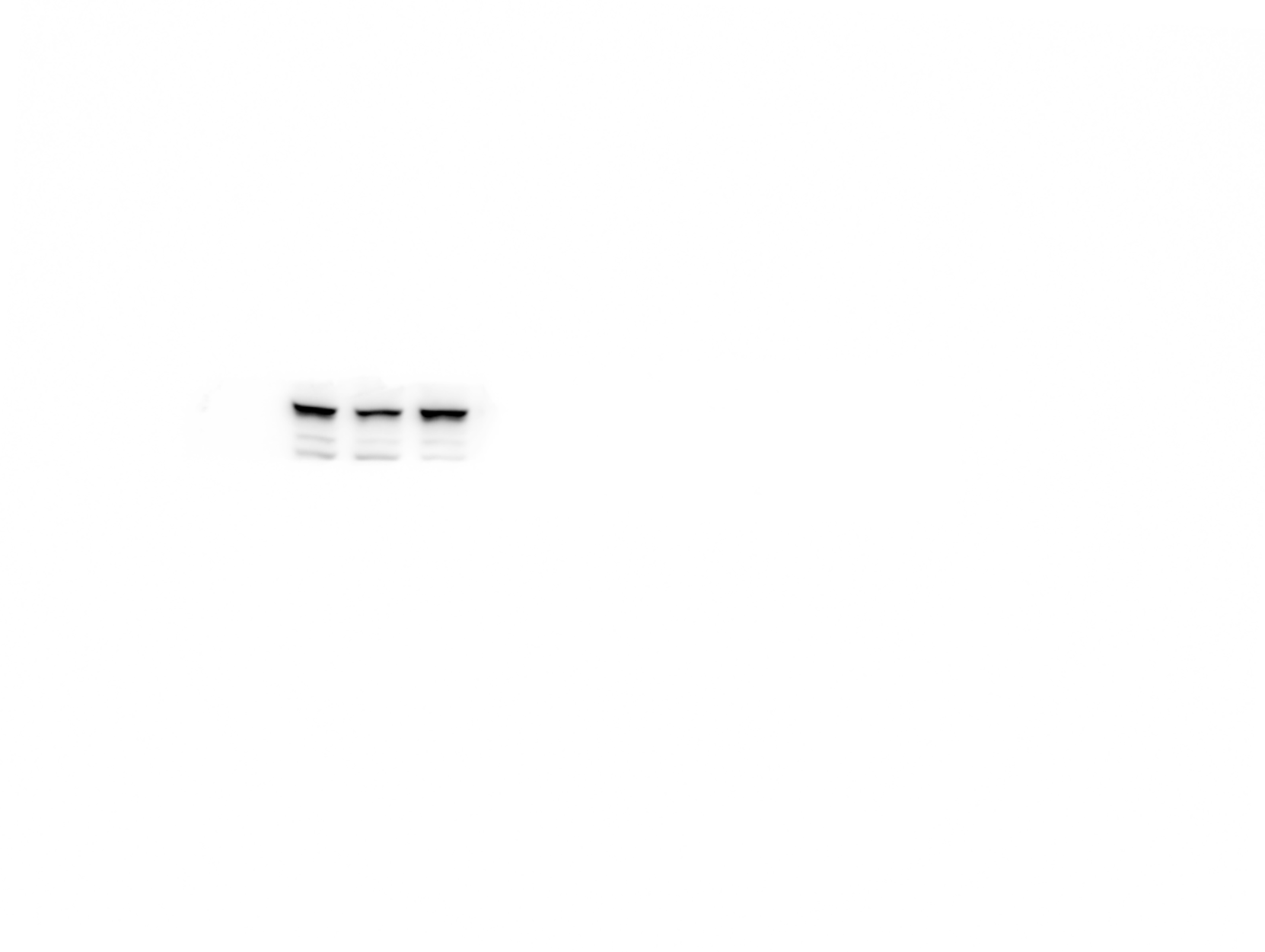

Supplement: Figure 5—source data 1. [file elife-68481-fig5-data1.zip › Figure 5-source data 1/5I source data/pAKT.tif]

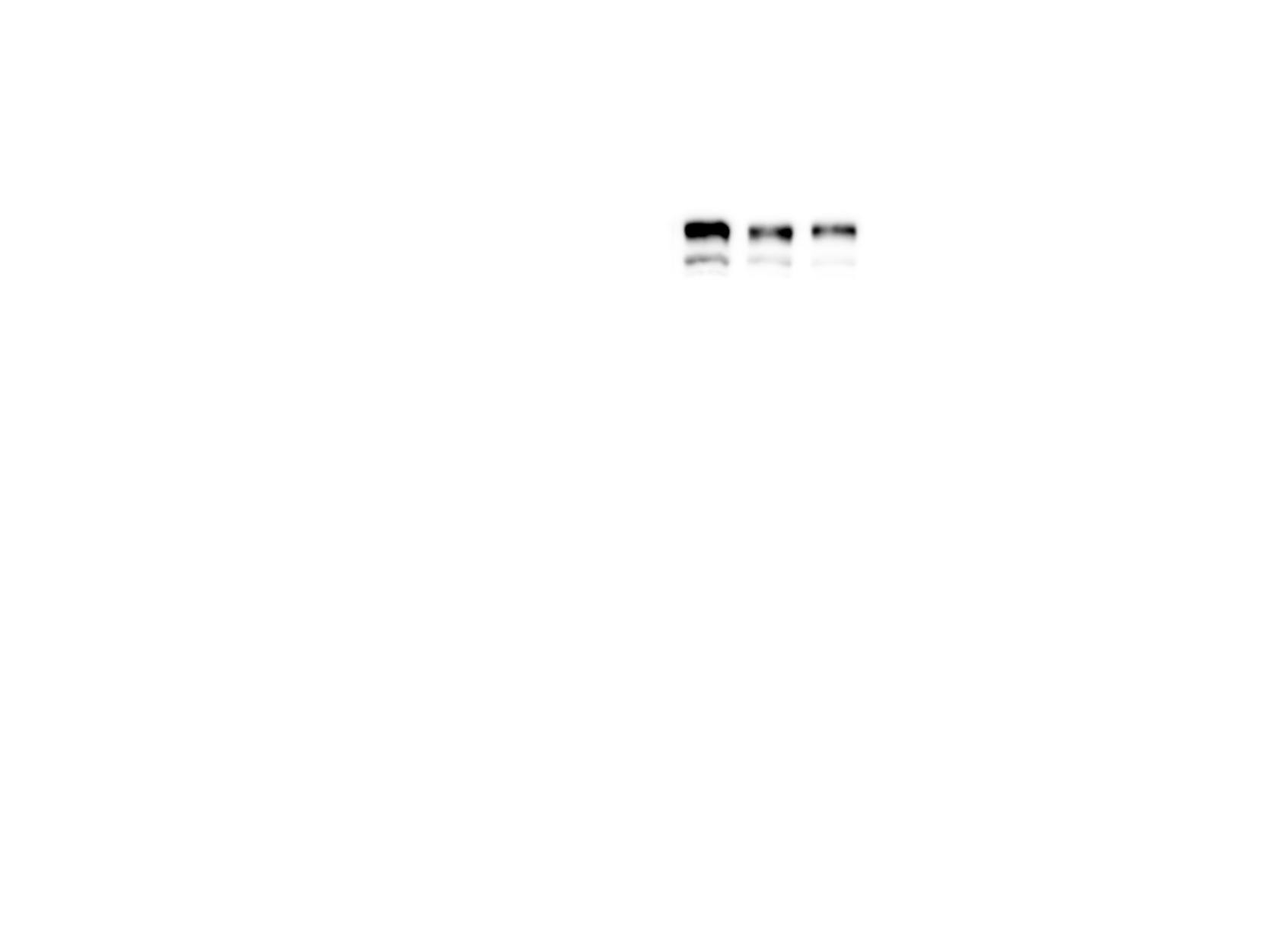

Supplement: Figure 5—source data 1. [file elife-68481-fig5-data1.zip › Figure 5-source data 1/5I source data/b1.tif]

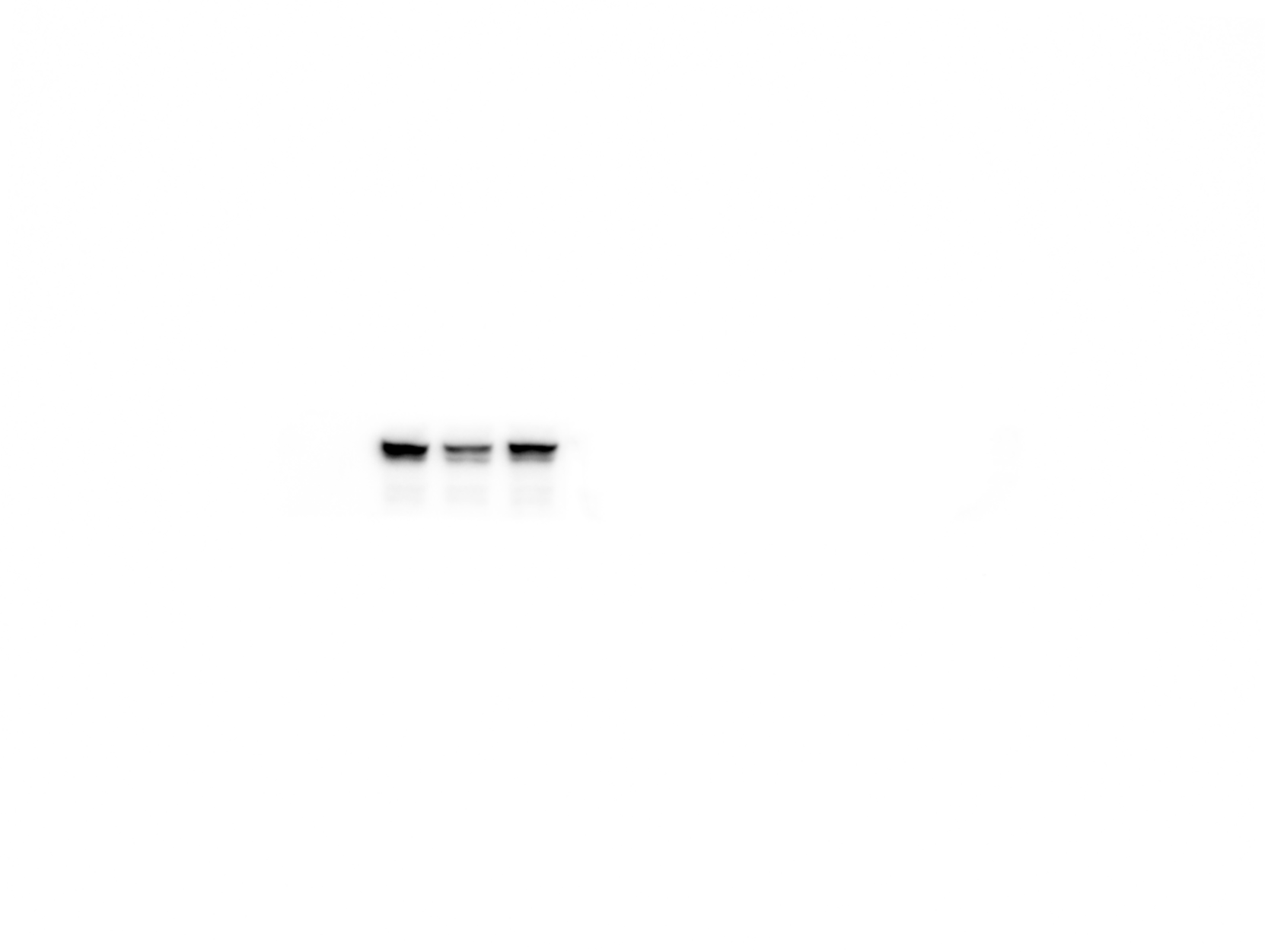

Supplement: Figure 5—source data 1. [file elife-68481-fig5-data1.zip › Figure 5-source data 1/5I source data/AKT.tif]

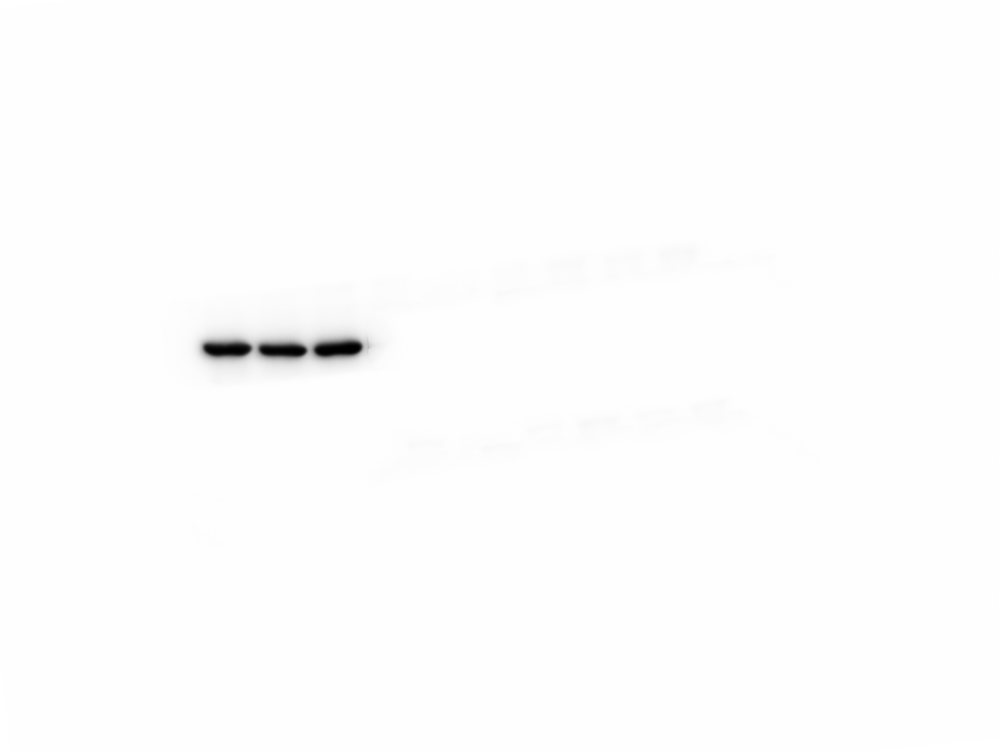

Supplement: Figure 5—source data 1. [file elife-68481-fig5-data1.zip › Figure 5-source data 1/5I source data/gapdh.tif]

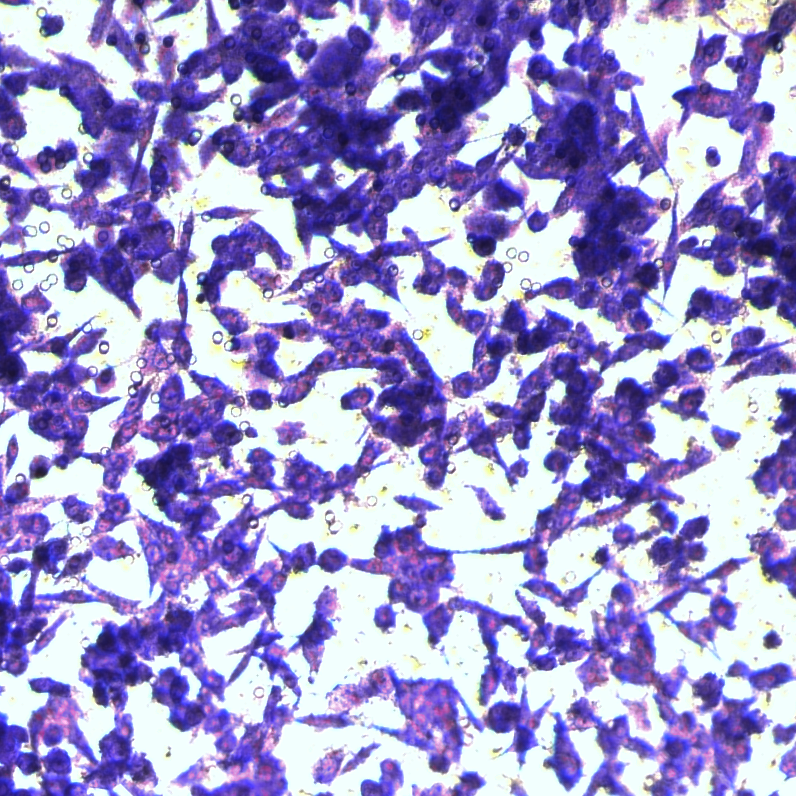

Supplement: Figure 5—source data 1. [file elife-68481-fig5-data1.zip › Figure 5-source data 1/5GH source data/transwell-C67399/231-DMSO.tif]

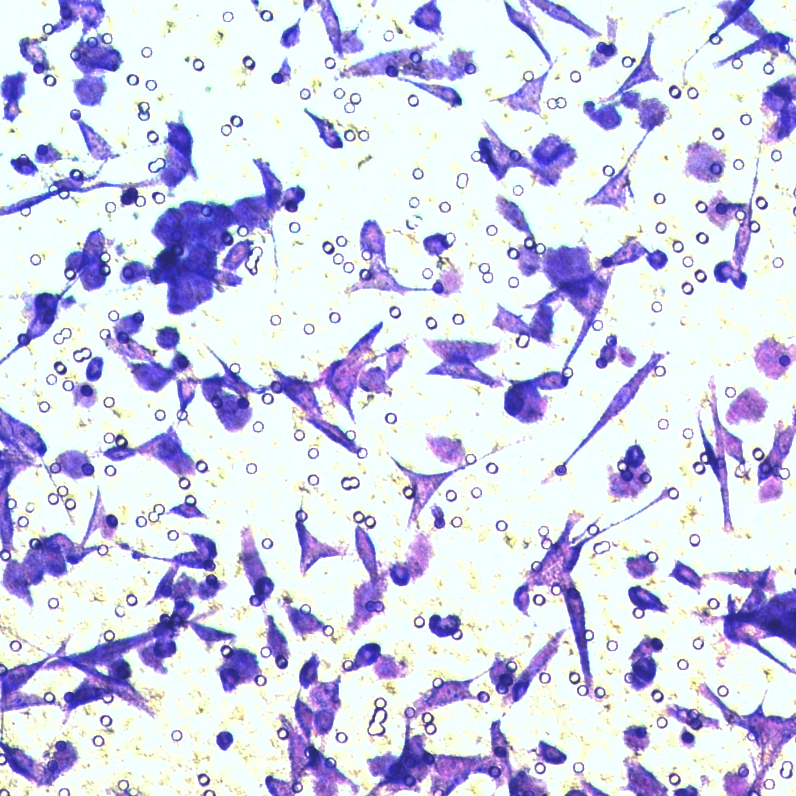

Supplement: Figure 5—source data 1. [file elife-68481-fig5-data1.zip › Figure 5-source data 1/5GH source data/transwell-C67399/231-C67399.tif]

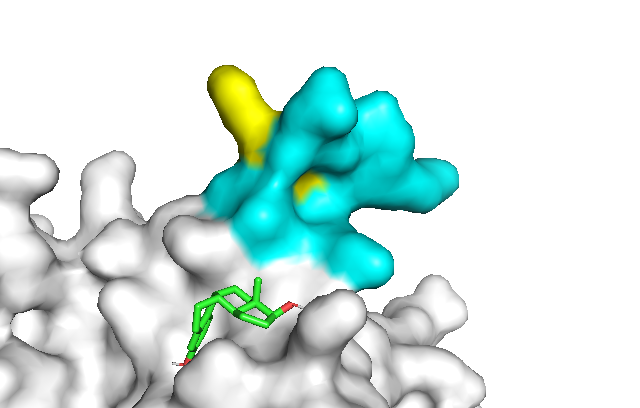

Supplement: Figure 5—figure supplement 1—source data 1. [file elife-68481-fig5-figsupp1-data1.zip › Figure 5-figure supplement 1 source data 1/S4B source data/8.png]

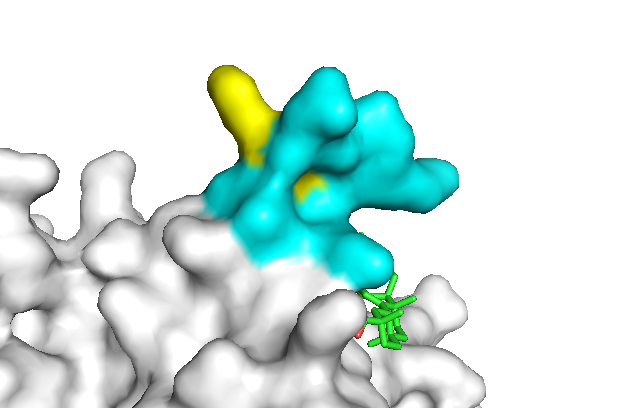

Supplement: Figure 5—figure supplement 1—source data 1. [file elife-68481-fig5-figsupp1-data1.zip › Figure 5-figure supplement 1 source data 1/S4B source data/4.png]

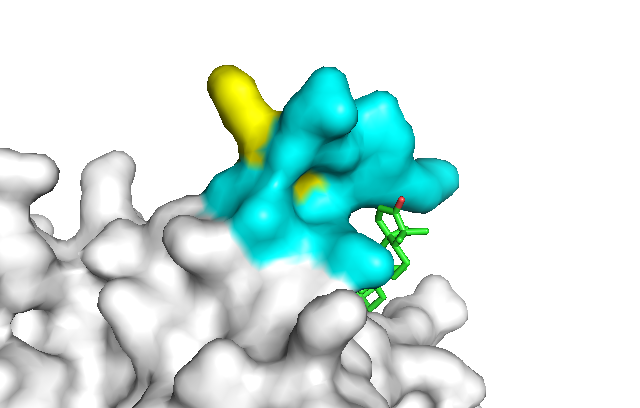

Supplement: Figure 5—figure supplement 1—source data 1. [file elife-68481-fig5-figsupp1-data1.zip › Figure 5-figure supplement 1 source data 1/S4B source data/5.png]

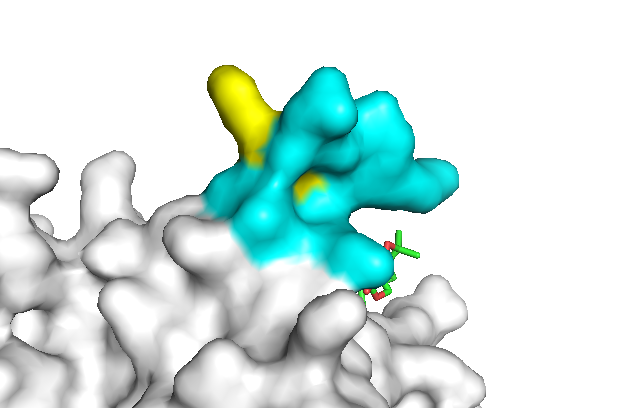

Supplement: Figure 5—figure supplement 1—source data 1. [file elife-68481-fig5-figsupp1-data1.zip › Figure 5-figure supplement 1 source data 1/S4B source data/7.png]

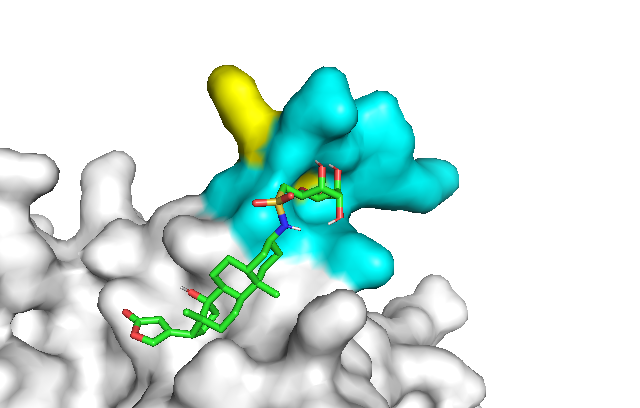

Supplement: Figure 5—figure supplement 1—source data 1. [file elife-68481-fig5-figsupp1-data1.zip › Figure 5-figure supplement 1 source data 1/S4B source data/6.png]

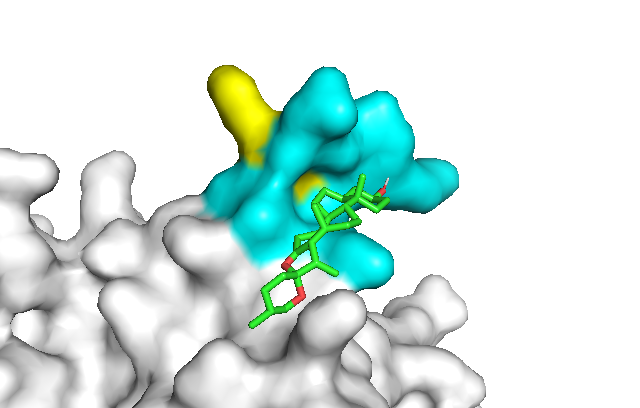

Supplement: Figure 5—figure supplement 1—source data 1. [file elife-68481-fig5-figsupp1-data1.zip › Figure 5-figure supplement 1 source data 1/S4B source data/2.png]

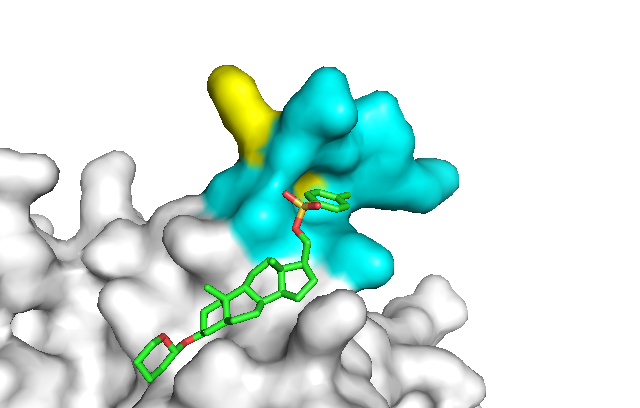

Supplement: Figure 5—figure supplement 1—source data 1. [file elife-68481-fig5-figsupp1-data1.zip › Figure 5-figure supplement 1 source data 1/S4B source data/3.png]

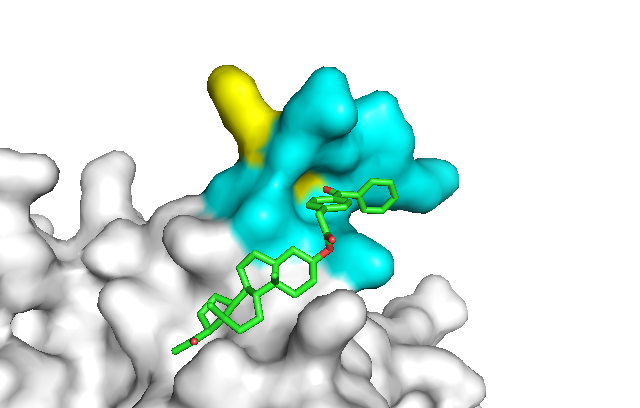

Supplement: Figure 5—figure supplement 1—source data 1. [file elife-68481-fig5-figsupp1-data1.zip › Figure 5-figure supplement 1 source data 1/S4B source data/1.png]

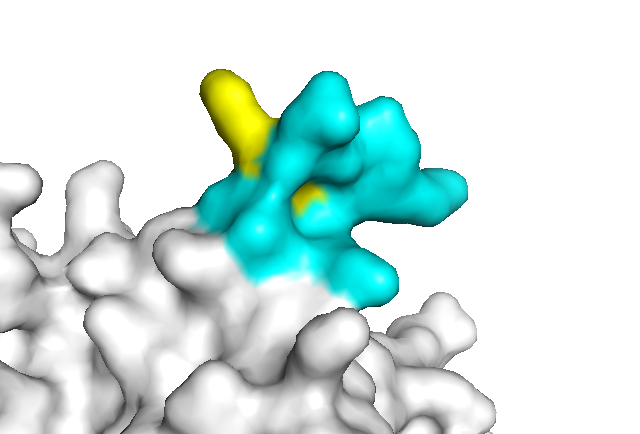

Supplement: Figure 5—figure supplement 1—source data 1. [file elife-68481-fig5-figsupp1-data1.zip › Figure 5-figure supplement 1 source data 1/S4B source data/0.png]

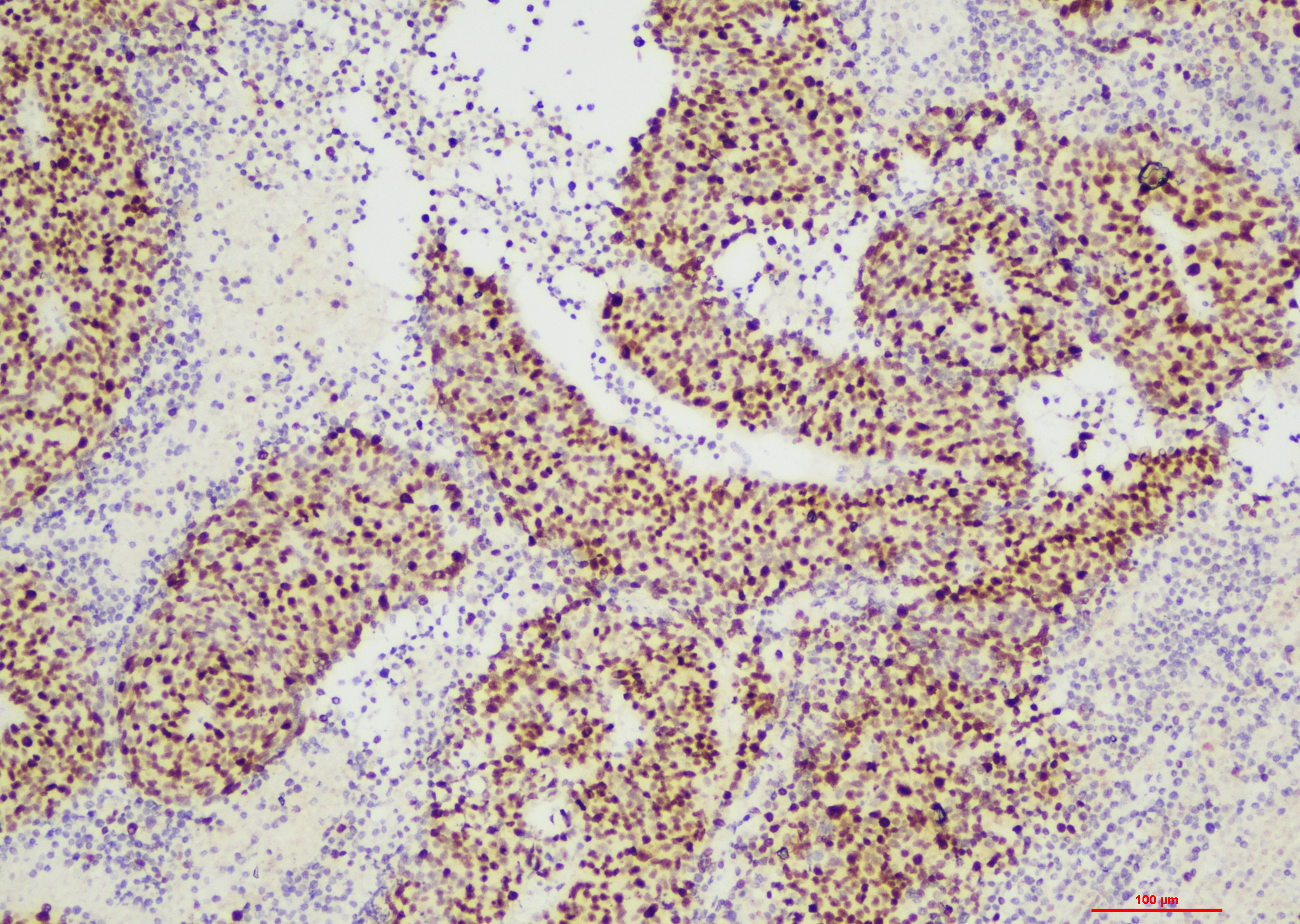

Supplement: Figure 6—source data 1. [file elife-68481-fig6-data1.zip › Figure 6-source data 1/6F source data/DMSO-ki67.tif]

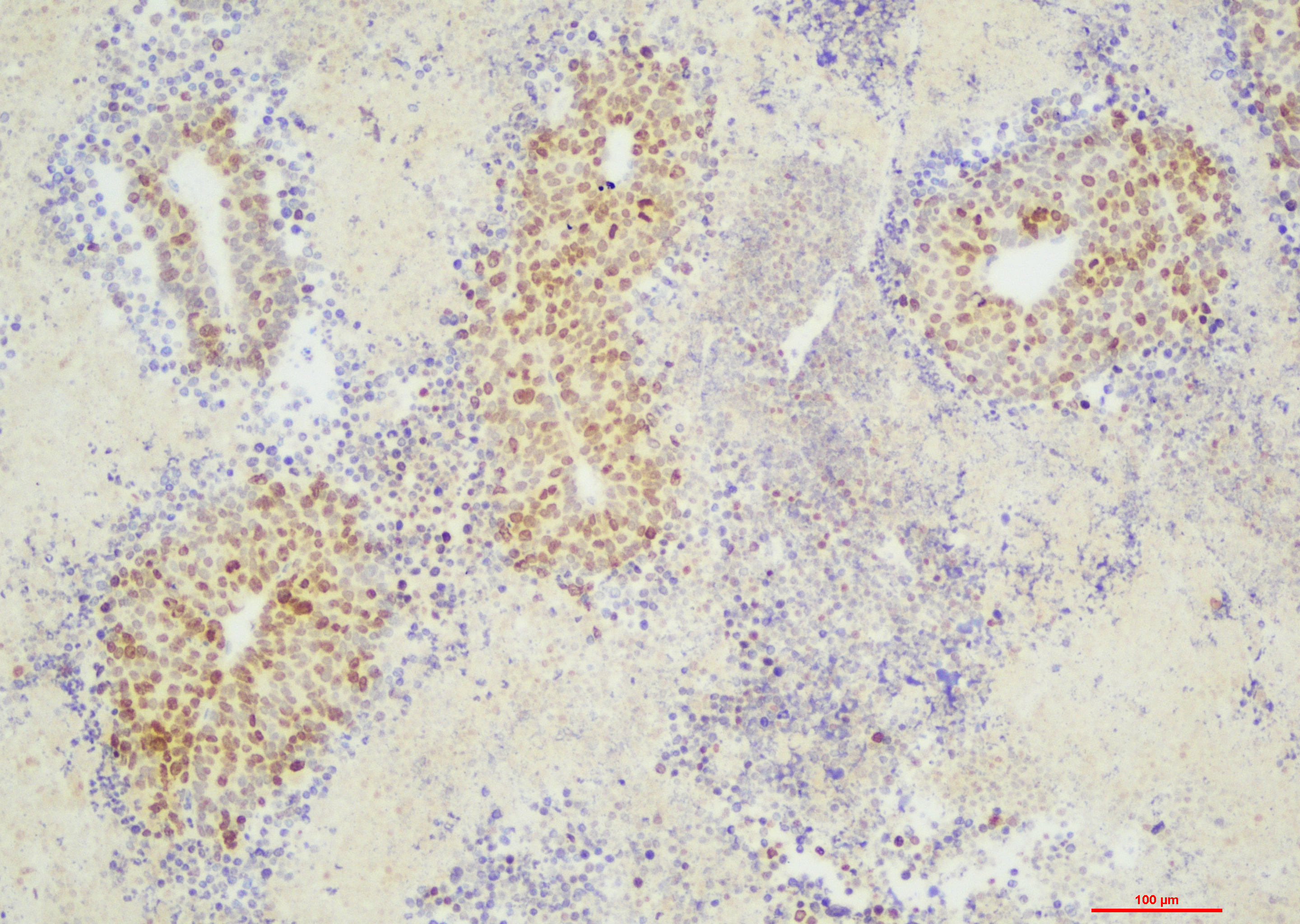

Supplement: Figure 6—source data 1. [file elife-68481-fig6-data1.zip › Figure 6-source data 1/6F source data/C67399-ki67.tif]

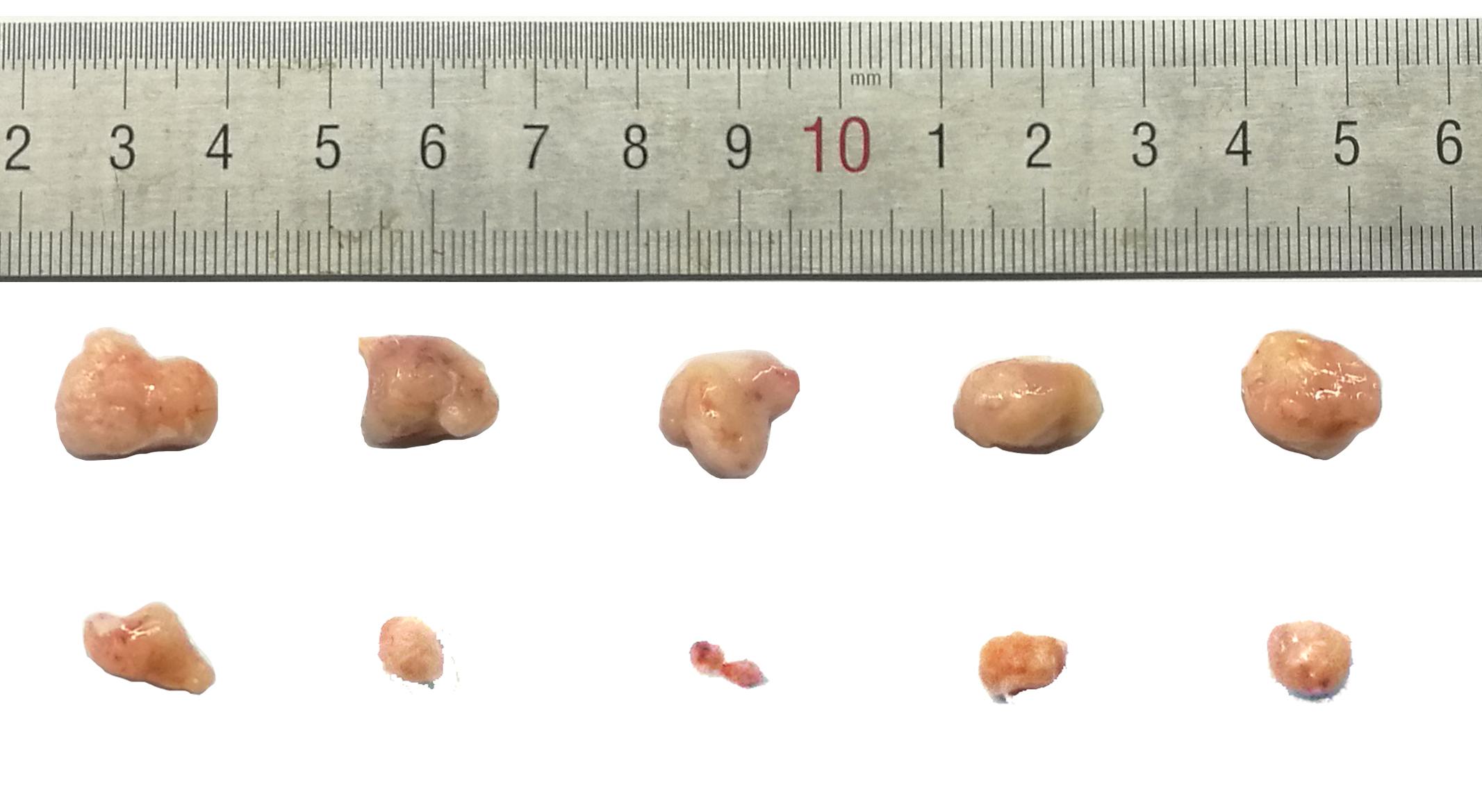

Supplement: Figure 6—source data 1. [file elife-68481-fig6-data1.zip › Figure 6-source data 1/6AB source data/xenograft tumor image.jpg]

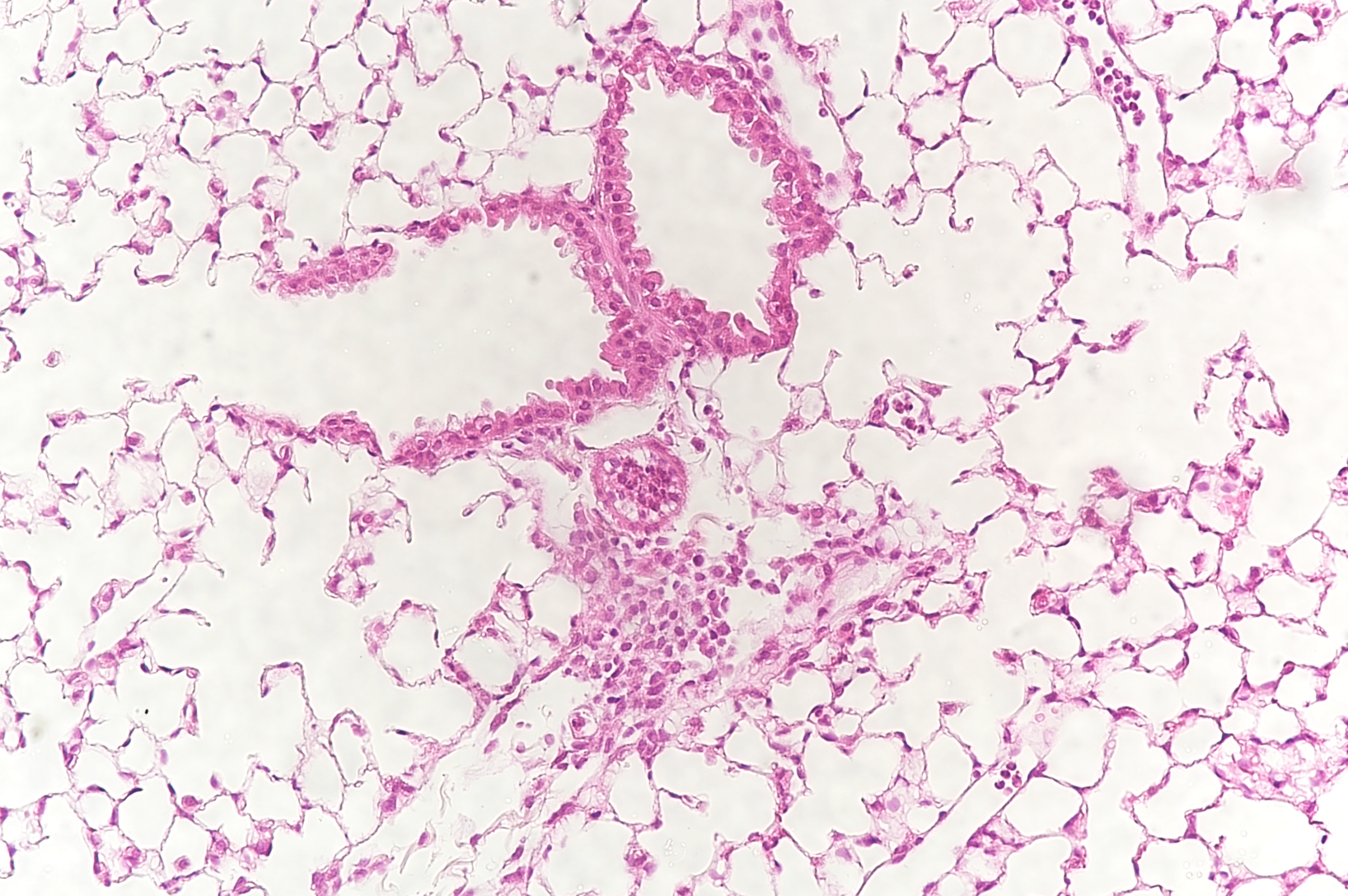

Supplement: Figure 6—source data 1. [file elife-68481-fig6-data1.zip › Figure 6-source data 1/6CDE source data/lung-20x-C67399.tif]
