# Supplementary material for: Binding blockade between TLN1 and integrin β1 represses triple-negative breast cancer
Source: eLife. 2022 Mar 14;11:e68481. doi: 10.7554/eLife.68481 (PMC8937232; doi:10.7554/eLife.68481)
Supplement: Figure 1—figure supplement 1—source data 1. [file elife-68481-fig1-figsupp1-data1.zip › Figure 1-figure supplement 1 source data 1/Figure 1-figure supplement 1 souce data 1.docx]

A-B

This part of the data is based on TIMER2.0 dataset on the TIMER website (<http://timer.cistrome.org/>).

C-D

This part of the data is based on TCGA dataset analysis on the UALCAN website (http://ualcan.path.uab.edu/).
