# Supplementary figures and images for: Binding blockade between TLN1 and integrin β1 represses triple-negative breast cancer (part 2 of 2)
Source: eLife. 2022 Mar 14;11:e68481. doi: 10.7554/eLife.68481 (PMC8937232; doi:10.7554/eLife.68481)

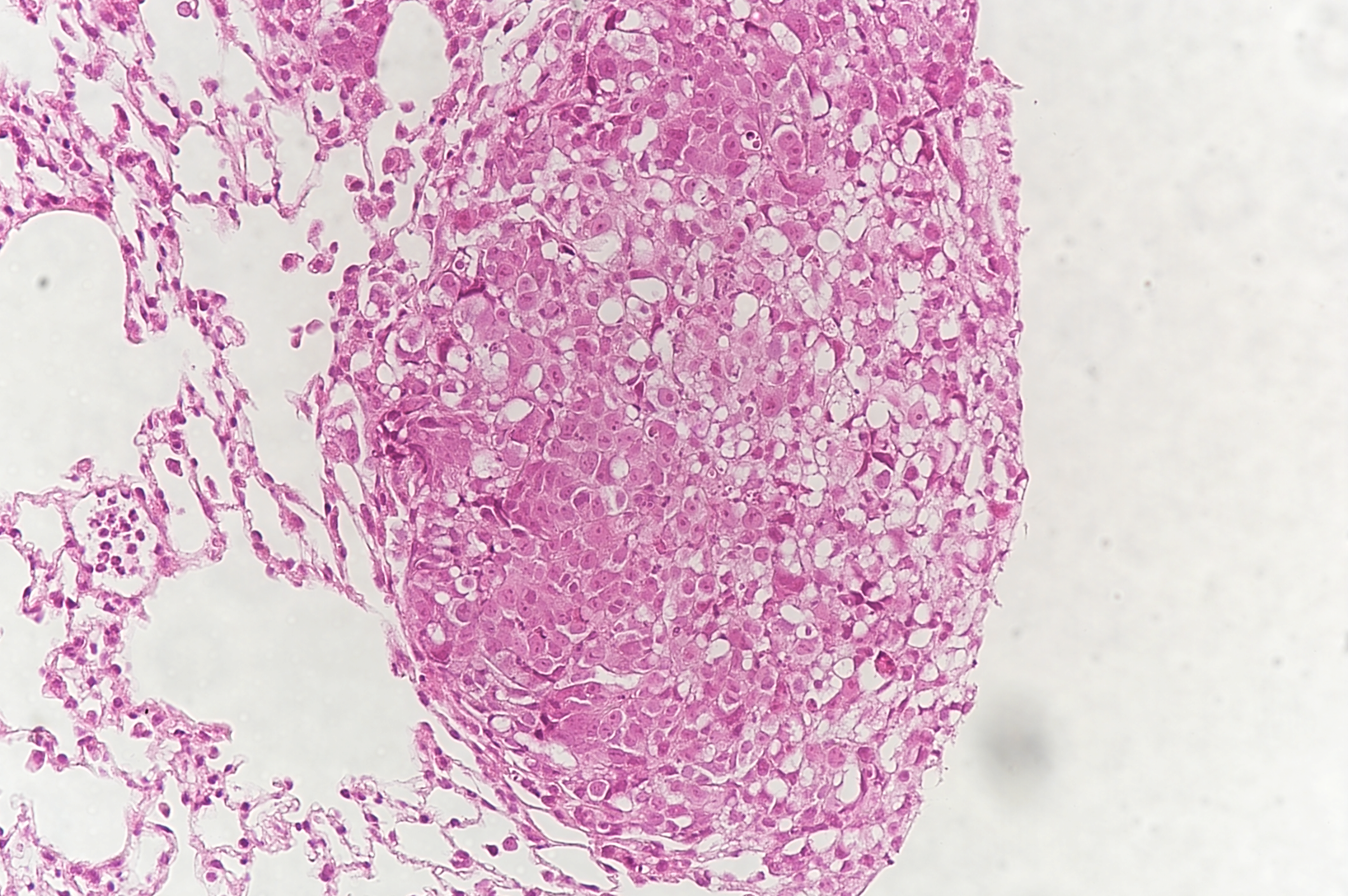

Supplement: Figure 6—source data 1. [file elife-68481-fig6-data1.zip › Figure 6-source data 1/6CDE source data/lung-20x-DMSO.tif]

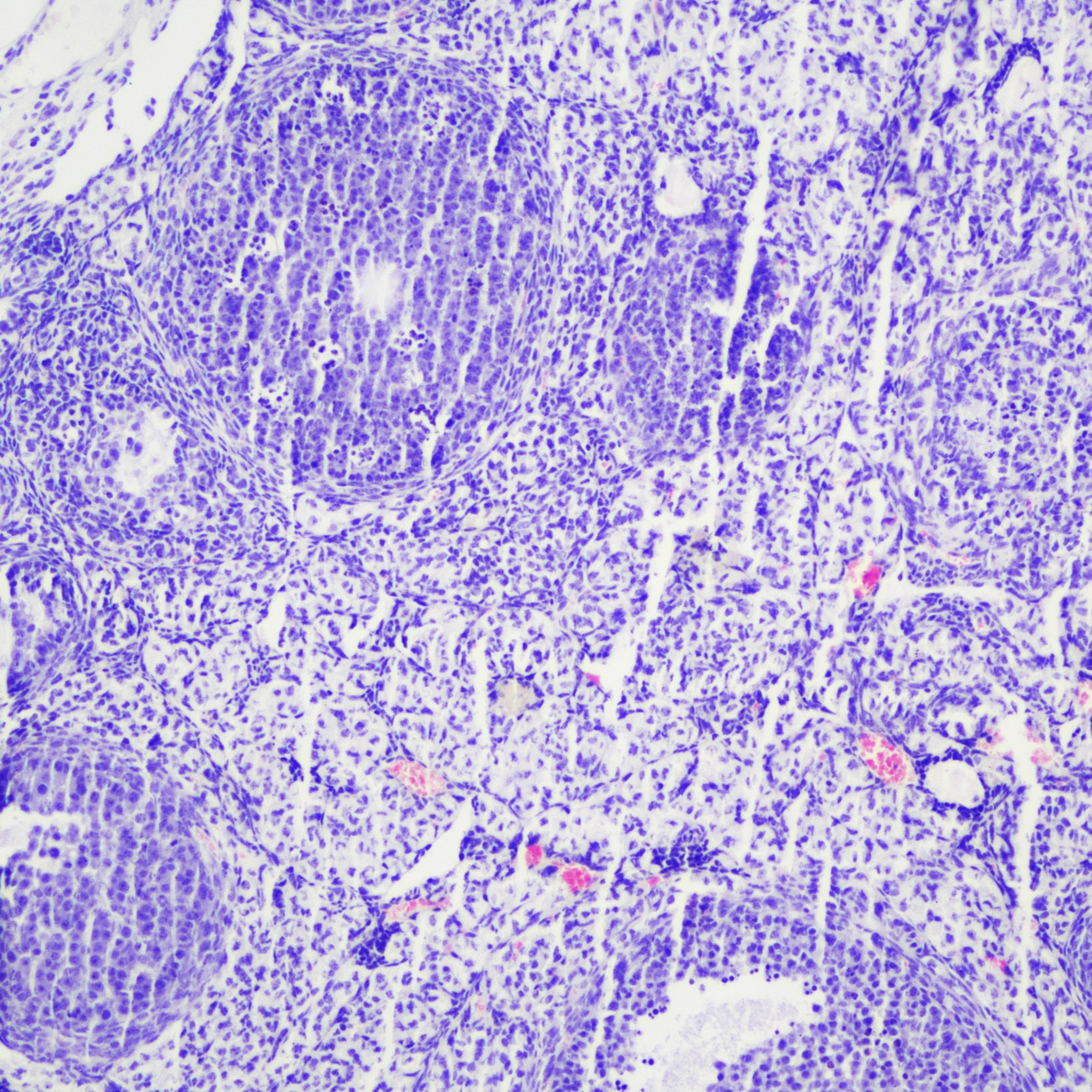

Supplement: Figure 6—figure supplement 1—source data 1. [file elife-68481-fig6-figsupp1-data1.zip › Figure 6-figure supplement 1 source data 1/S5A source data/overy.jpg]

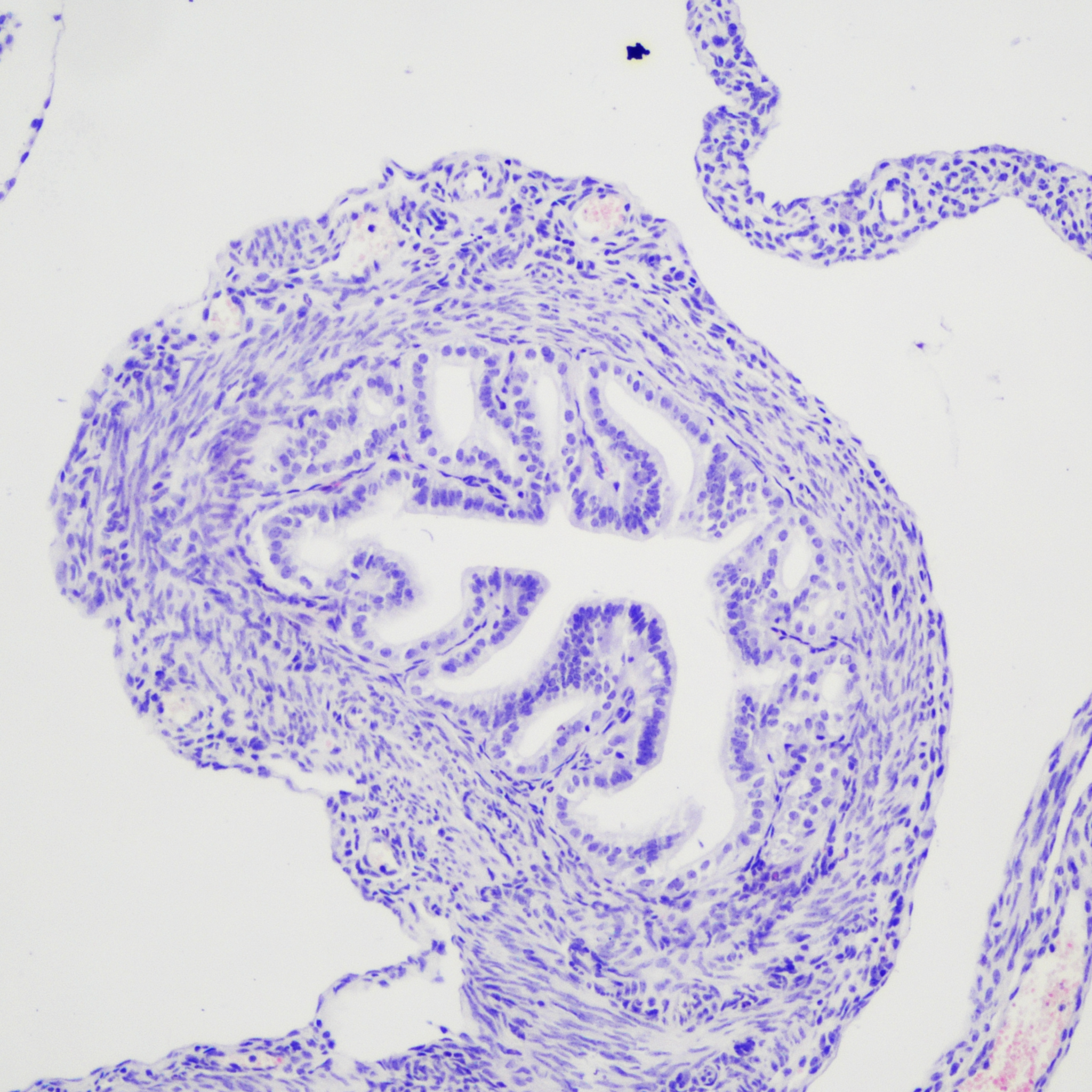

Supplement: Figure 6—figure supplement 1—source data 1. [file elife-68481-fig6-figsupp1-data1.zip › Figure 6-figure supplement 1 source data 1/S5A source data/oviduct.jpg]

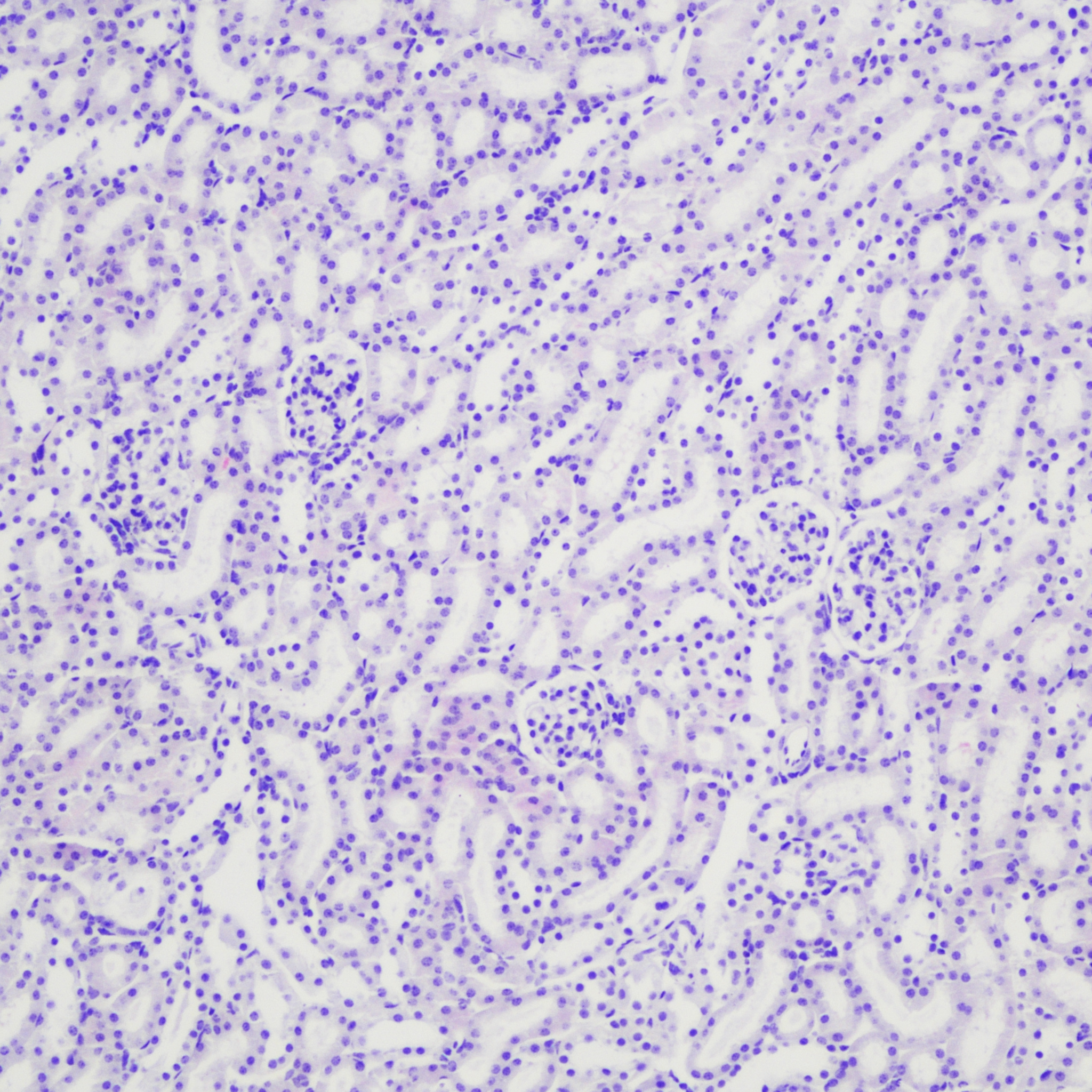

Supplement: Figure 6—figure supplement 1—source data 1. [file elife-68481-fig6-figsupp1-data1.zip › Figure 6-figure supplement 1 source data 1/S5A source data/kidney.jpg]

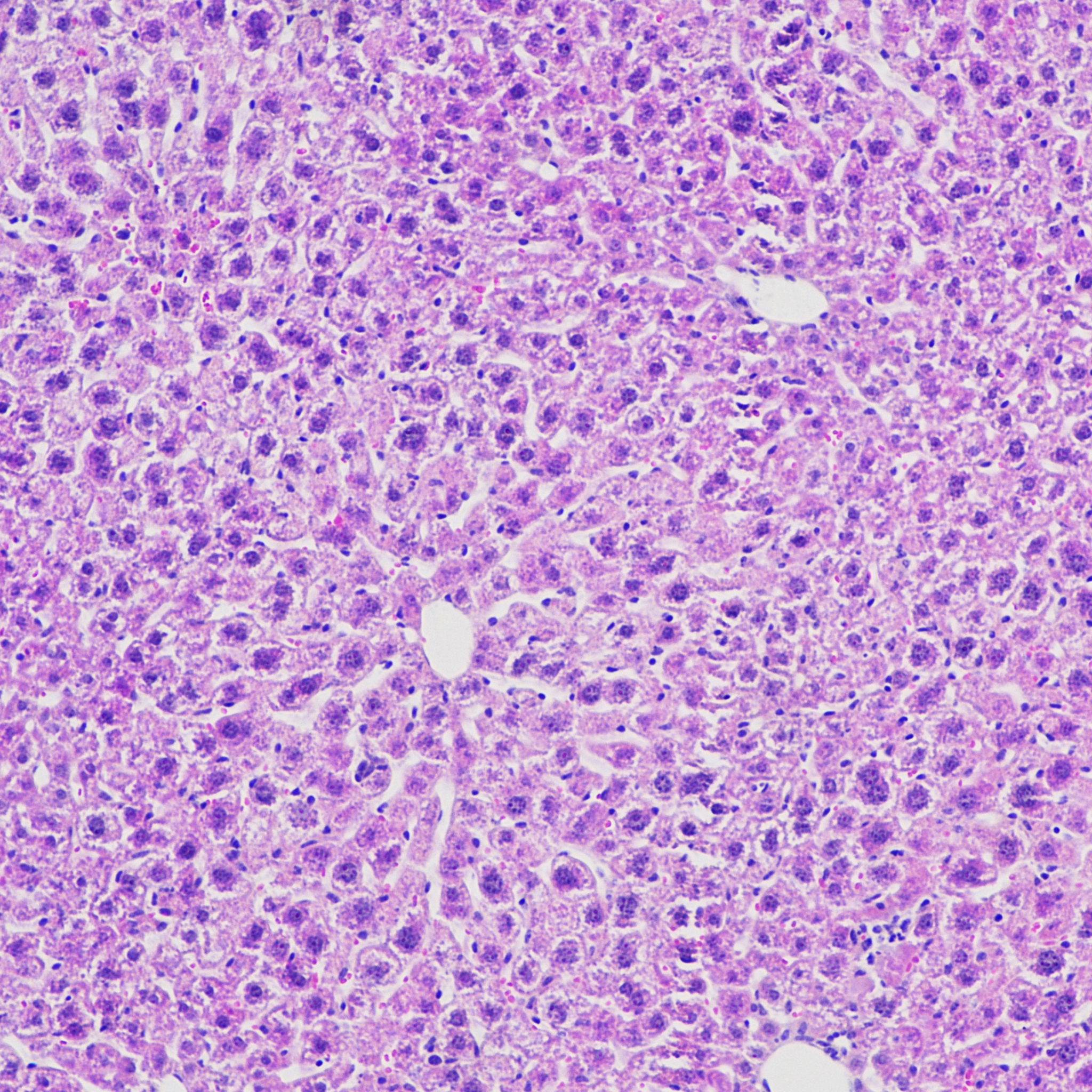

Supplement: Figure 6—figure supplement 1—source data 1. [file elife-68481-fig6-figsupp1-data1.zip › Figure 6-figure supplement 1 source data 1/S5A source data/liver.jpg]

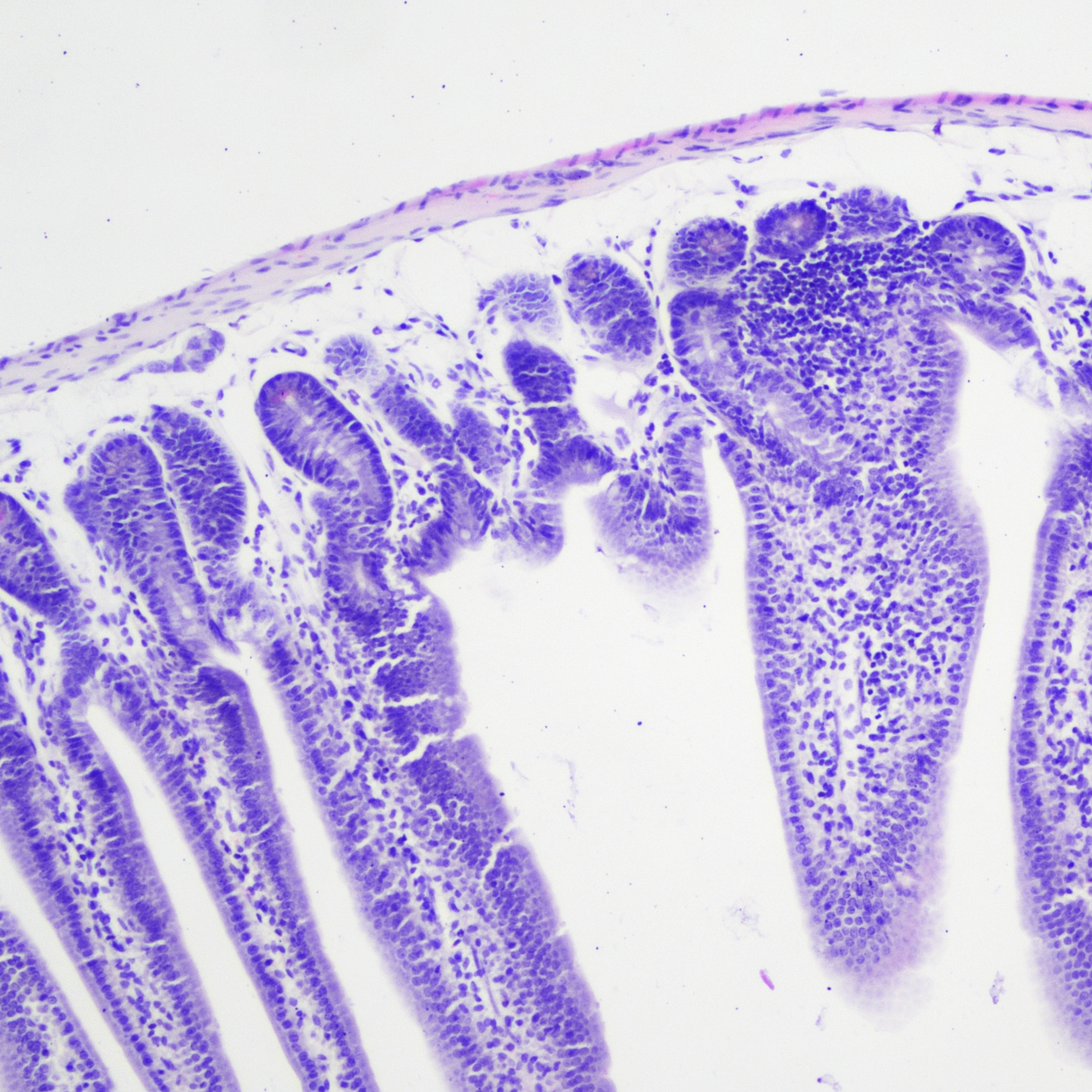

Supplement: Figure 6—figure supplement 1—source data 1. [file elife-68481-fig6-figsupp1-data1.zip › Figure 6-figure supplement 1 source data 1/S5A source data/colon.jpg]

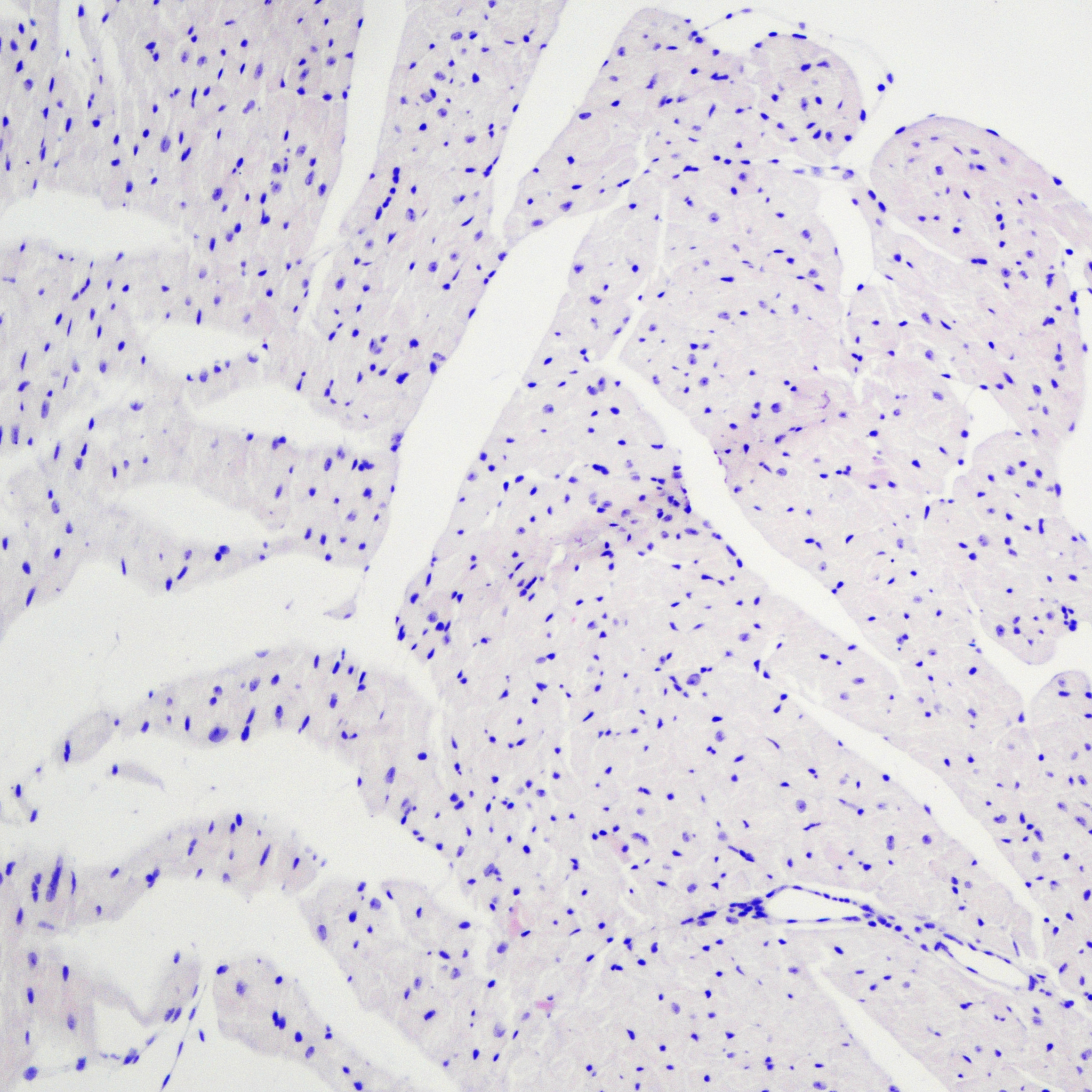

Supplement: Figure 6—figure supplement 1—source data 1. [file elife-68481-fig6-figsupp1-data1.zip › Figure 6-figure supplement 1 source data 1/S5A source data/heart.jpg]

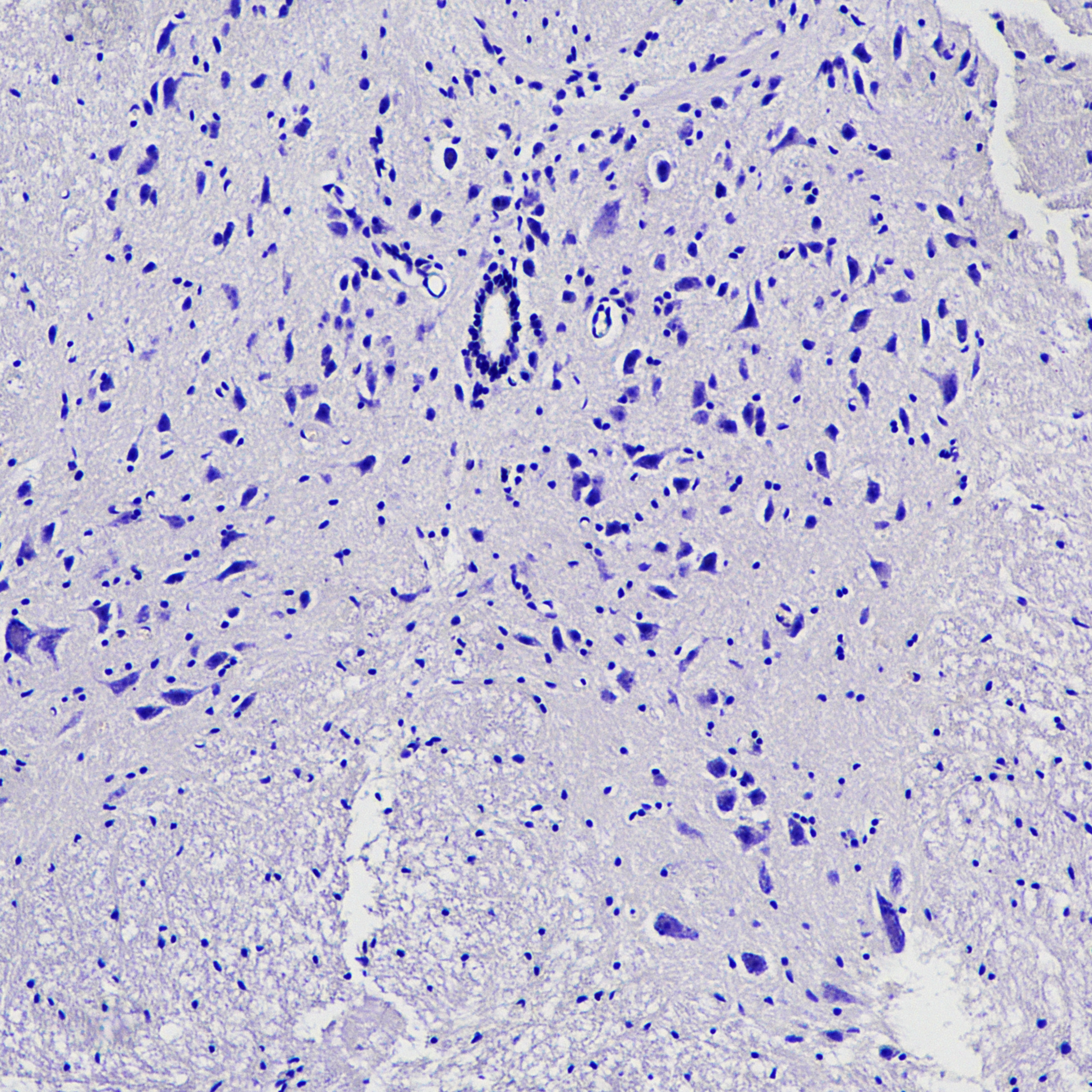

Supplement: Figure 6—figure supplement 1—source data 1. [file elife-68481-fig6-figsupp1-data1.zip › Figure 6-figure supplement 1 source data 1/S5A source data/marrow.jpg]

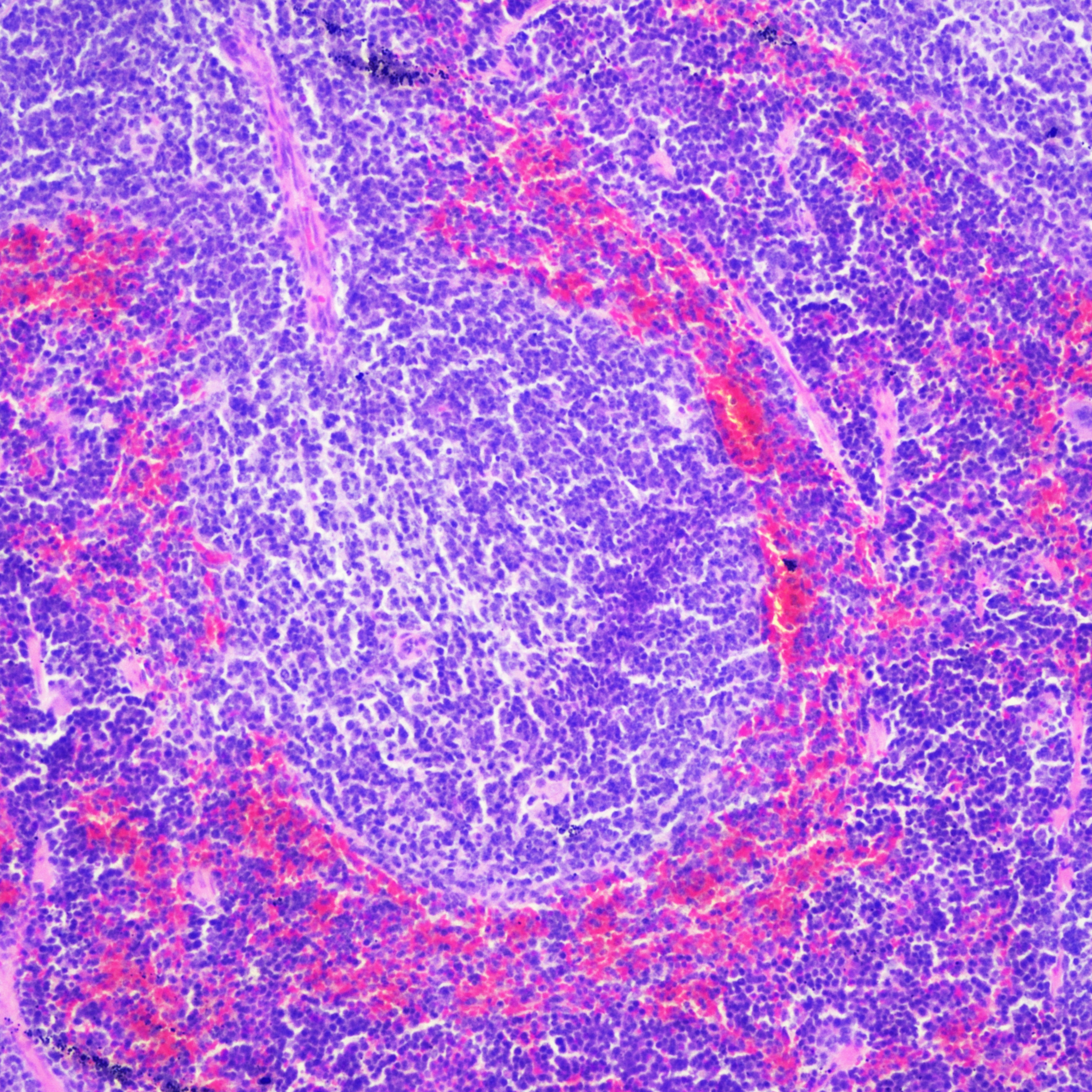

Supplement: Figure 6—figure supplement 1—source data 1. [file elife-68481-fig6-figsupp1-data1.zip › Figure 6-figure supplement 1 source data 1/S5A source data/speen.jpg]

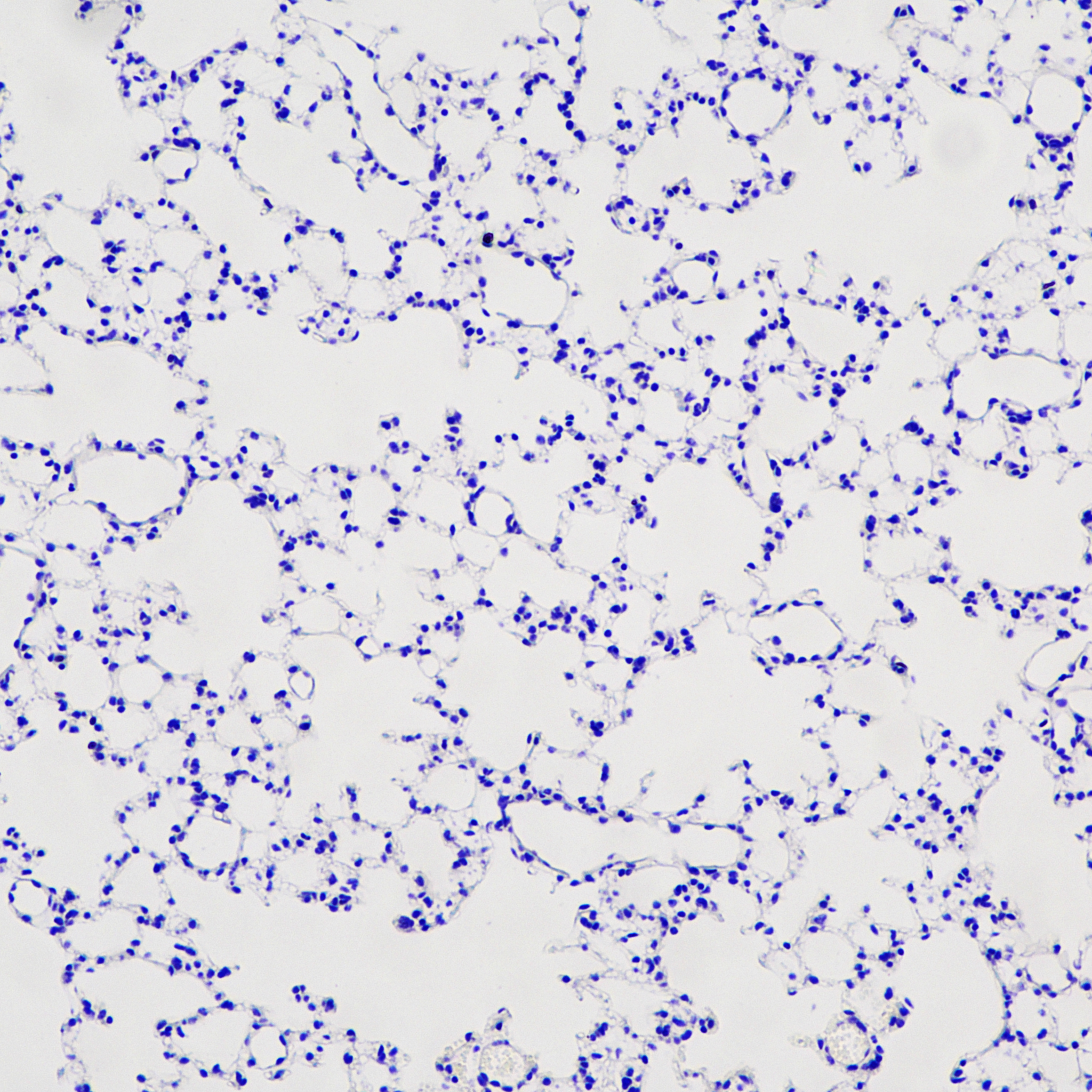

Supplement: Figure 6—figure supplement 1—source data 1. [file elife-68481-fig6-figsupp1-data1.zip › Figure 6-figure supplement 1 source data 1/S5A source data/lung.jpg]

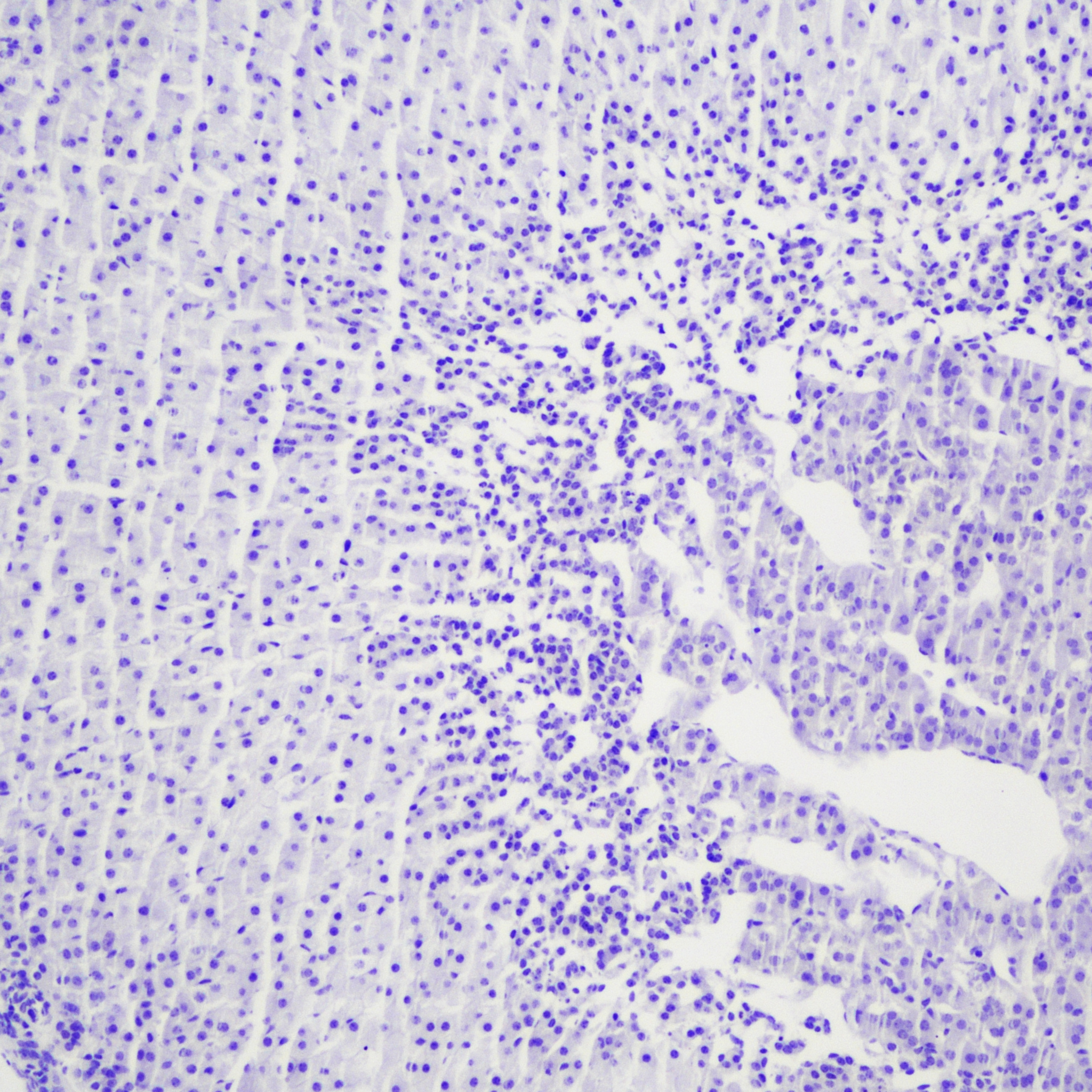

Supplement: Figure 6—figure supplement 1—source data 1. [file elife-68481-fig6-figsupp1-data1.zip › Figure 6-figure supplement 1 source data 1/S5A source data/adrenal gland.jpg]

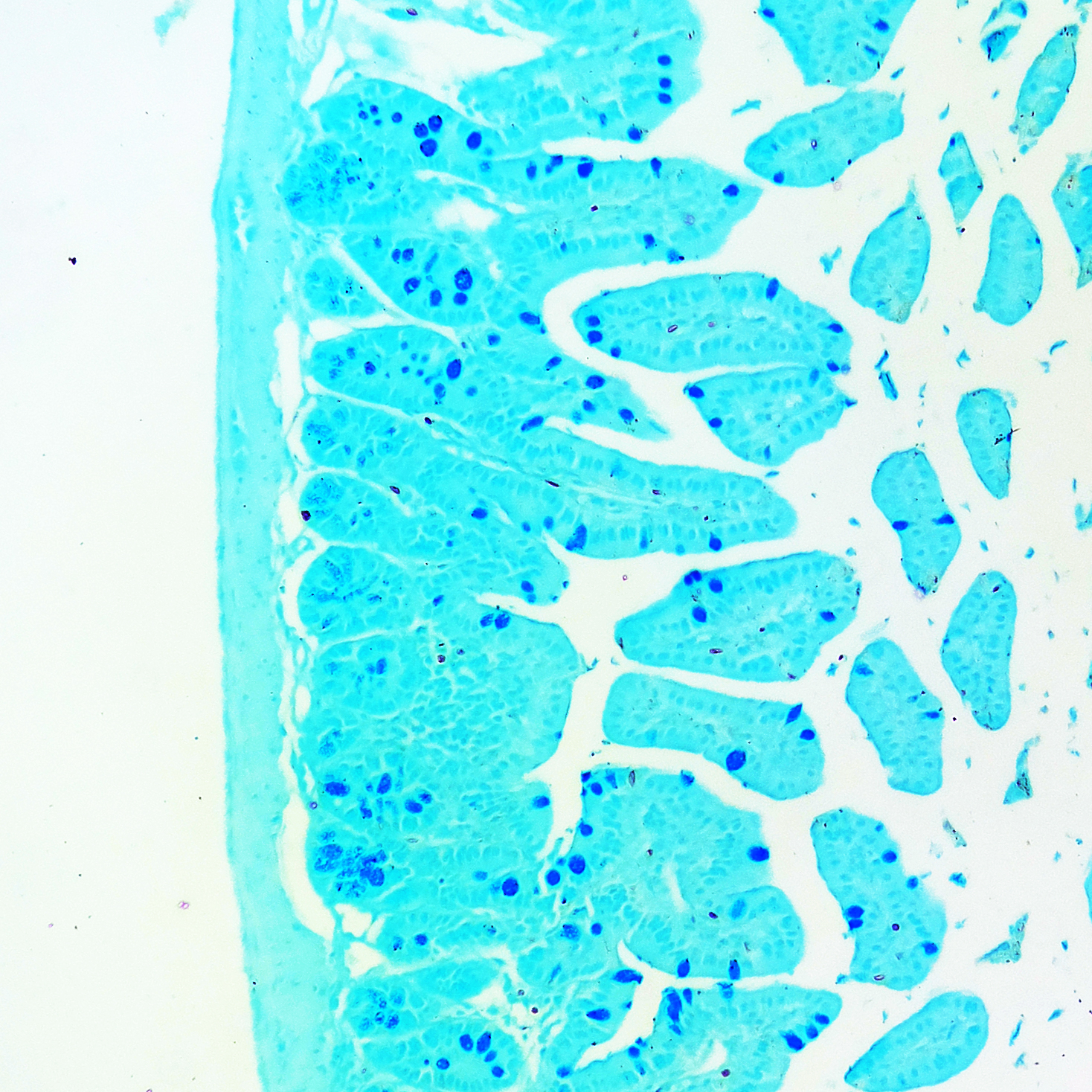

Supplement: Figure 6—figure supplement 1—source data 1. [file elife-68481-fig6-figsupp1-data1.zip › Figure 6-figure supplement 1 source data 1/S5B source data/C67399.jpg]

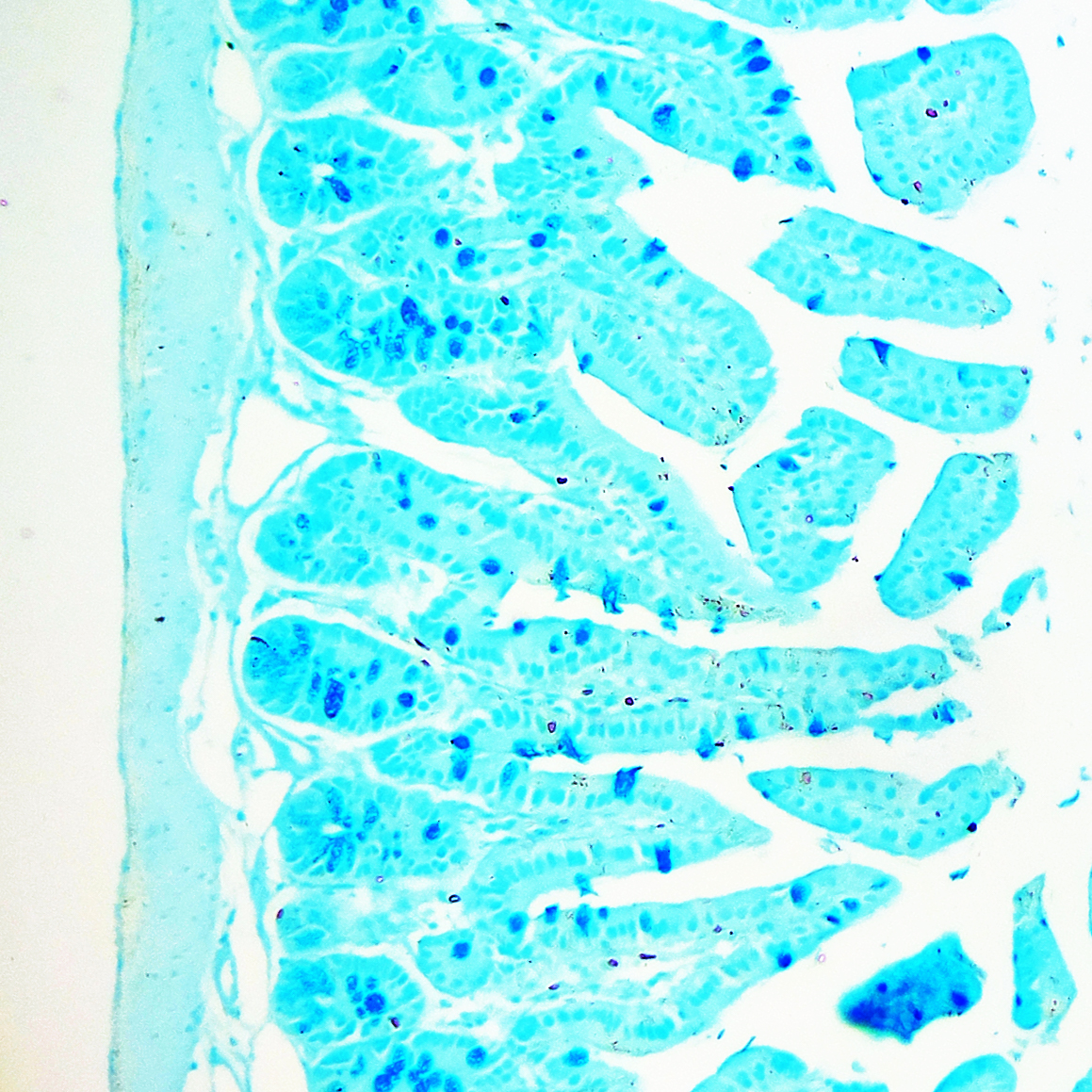

Supplement: Figure 6—figure supplement 1—source data 1. [file elife-68481-fig6-figsupp1-data1.zip › Figure 6-figure supplement 1 source data 1/S5B source data/DMSO.jpg]
